# Controls on natural hydrogen generation during serpentinization of mantle rocks

**DOI:** 10.1038/s41467-026-73920-5

**Published:** 2026-06-11

**Authors:** Rodolfo Christiansen, Mohamed Sobh, Christian Ostertag-Henning, Guido Gianni, Nicolas Saspiturry, Sebastien Chevrot, Victoria Langenheim, Javier García-Pintado, Gerald Gabriel

**Affiliations:** 1 LIAG Institute for Applied Geophysics, Hannover, Germany; 2 Federal Institute for Geosciences and Natural Resources (BGR), Hannover, Germany; 3 Institute of Geophysics, Czech Academy of Sciences, Prague, Czechia; 4 Géosciences Montpellier, Université de Montpellier, CNRS, Montpellier, France; 5 Géosciences Environnement Toulouse (GET), UMR 5563, Observatoire Midi-Pyrénées, CNRS, Université de Toulouse, Toulouse, France; 6 United States Geological Survey (USGS), Moffett Field, California, USA; 7 MARUM - Center for Marine Environmental Sciences, University of Bremen, Bremen, Germany; 8 Institute of Earth System Sciences, Leibniz University Hannover, Hannover, Germany; 9 Mantle8, Sassenage, France

**Keywords:** Geochemistry, Geophysics, Mineralogy

## Abstract

Mantle rocks undergoing serpentinization can generate significant amounts of natural hydrogen, yet the rates and controlling processes remain poorly understood. Here, we constrain the possible hydrogen generation rates in two distinct mantle rock types, the fertile lherzolites of the Western Pyrenees and the depleted harzburgites of Northern California, to relatively low rates of ~0.1 to ~0.5 tonnes H₂ yr⁻¹ km⁻³ of reactive rock. When integrated over the full reactive volumes, this corresponds to total production rates of ~300 to ~600 tonnes H₂ yr⁻¹. By combining three-dimensional geophysical inversion with numerical modelling of fluid-rock processes, we show that hydrogen generation rates are mainly limited by H₂ saturation in the fluid and reaction kinetics. Under these constraints, hydrogen generation in mantle-derived serpentinization systems proceeds slowly, making rapid large-scale replenishment unlikely and suggesting that large, economically relevant accumulations, would require timescales of thousands to tens of thousands of years to develop.

## Abstract (web summary)

Natural hydrogen generation during mantle rock serpentinization is slow, limited by fluid saturation and reaction kinetics, suggesting large accumulations require millennia to develop.

## Introduction

Serpentinization is an abiotic reaction involving the hydration of ultramafic rocks, in which Fe(II) in primary minerals is oxidised to Fe(III) in secondary phases, reducing hydrogen atoms in water molecules to molecular hydrogen (H_2_). This process is one of the primary natural mechanisms for hydrogen generation^1–5^. The generation rate of molecular hydrogen (hereafter referred to as hydrogen or H_2_) and the rate of serpentinization are highly dependent on temperature, with maximum rates between 200 °C and 300 °C^2,6^. This mineral replacement reaction also affects the petrophysical properties of the rocks by decreasing density and seismic velocities, associated most often with increasing magnetic susceptibility. These contrasts allow serpentinites to be distinguished from fresh mantle rocks based on their geophysical characteristics, such as gravity, magnetic, and seismic properties^7–9^.

In recent years, natural hydrogen has emerged as a potential low-carbon energy resource^10^. However, growing evidence suggests that hydrogen generation associated with mantle rocks serpentinization proceeds over long timescales, with limited generation rates, even in the most favourable conditions, such as serpentinization of deeply buried olivine-rich rocks^11,12^. Major obstacles to quantifying natural hydrogen generation include the inaccessibility of deep crustal environments, a limited understanding of water availability, and uncertainties in reaction kinetics under low water-to-rock ratios (W/R), defined as the fluid-to-rock mass ratio. Serpentinization is nevertheless associated with some of the largest known natural hydrogen occurrences worldwide, with high hydrogen concentrations measured in emanating gases and associated flow-rate estimates of several hundred tonnes per year^13,14^. In contrast, model-based estimates remain highly uncertain. For example, in the Western Pyrenees, reported values range from several hundred thousand^15^ to several million tonnes of hydrogen produced per year^16^. These high estimates largely reflect approaches that quantify cumulative hydrogen generation potential, often assuming efficient and sustained fluid-rock interaction and neglecting explicit constraints related to fluid flows, reaction kinetics, and hydrogen saturation in the fluids. As a result, such values are not directly comparable to observed hydrogen flow rates in similar geological settings.

Here, we present a methodological framework for quantifying natural H₂ generation during serpentinization that explicitly constrains geological parameters within well-defined petro-physico-chemical boundary conditions. Unlike previous approaches that estimate cumulative generation potential from rock volumes alone, this framework combines fluid-rock thermodynamics with explicit kinetic and saturation limits, representing a fundamental shift from potential-based to process-resolved hydrogen generation estimates. Hydrogen generation is evaluated using a sequential, quasi-dynamic modelling framework in which thermodynamic equilibrium is assumed locally for each fluid-rock interaction event, and sustained production rates emerge as the serpentinization front advances under explicit kinetic limitations. Fluid transport is parameterised through fracture-controlled flow and diffusive exchange, while feedbacks from fluid saturation and lithology-dependent alteration are explicitly resolved, together with rock-specific petrophysical properties including mineral-bound water uptake, pressure-dependent hydrogen solubility, and porosity-controlled reaction-front propagation. We apply this model to two different geological areas, the Western Pyrenees and Northern California, selected for their good data availability and contrasting mantle rock compositions. The first case involves relatively fertile mantle rocks rich in olivine and orthopyroxene^17,18^, whereas the second is dominated by less reactive rocks that represent residues of high degrees of partial melting^19^. By integrating geological, geophysical, and physico-chemical constraints, we provide first-order estimates of hydrogen production rates and show that, at large scales, mantle-derived natural hydrogen behaves as a slowly replenished geological resource.

## Results

### Western Pyrenees

**Fig. 1:**
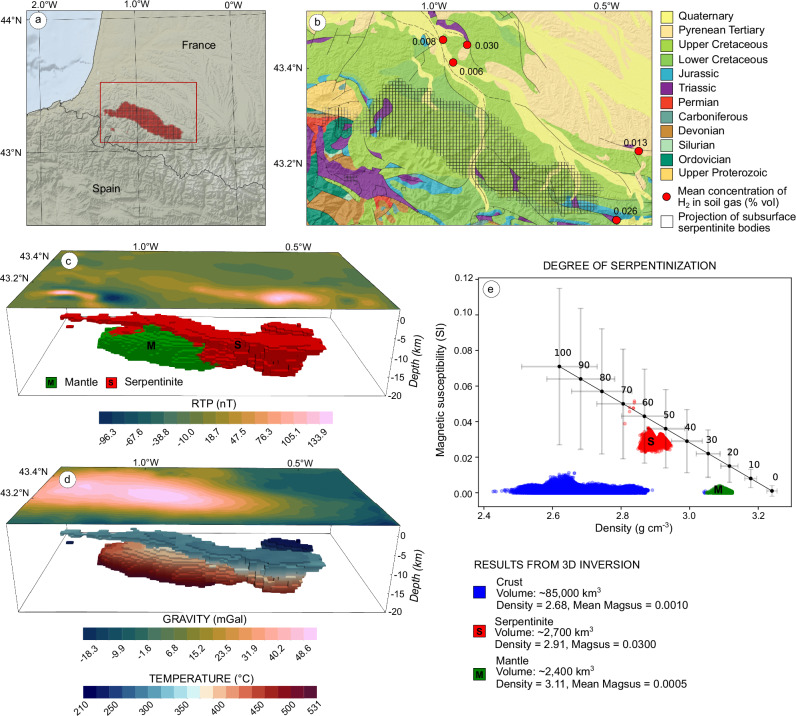
Results of source rock volumes for the Western Pyrenees. **a** Index map of study area (red box). The red polygons show modelled serpentinite extent projected onto the surface. **b** Age of geologic units in the study area (modified from ref. ^68^). Polygons show modelled serpentinite blocks projected onto the surface. Red circles represent H_2_ measurements (%) in soil gases (modified from ref. ^23^). **c** Reduced to the pole magnetic anomalies (RTP) for the area (modified from ref. ^69^ and ref. ^26^) with 3D blocks showing rock classification based on joint inversion of gravity and magnetic data. **d** Residual Bouguer gravity anomalies for the area (modified from ref. ^26^) with 3D blocks showing temperature distribution. **e** Results of the Gaussian Mixture Model (GMM) indicating the degree of serpentinization based on density and magnetic susceptibility results. Values are projected to the closest serpentinization point in the line. Crustal rocks correspond to an averaged petrophysical representation of the heterogeneous continental crustal assemblage included in the model (e.g., granitoids, gneisses, schists, locally mafic to ultramafic rocks, and metasedimentary units).

The Western Pyrenees (Fig. 1a, b) consist of a tectonic wedge, where mantle-derived rocks, mainly spinel lherzolites^17,18^, were exhumed during middle Cretaceous rifting^20,21^, and tectonically uplifted to their current shallow position (~ 10 km) as a result of Eocene-Oligocene convergence, which ended about 25 Ma ago^22–25^. Since then, tectonic conditions have remained relatively stable, which provides a prolonged window that kept the exhumed mantle rocks within optimum pressure-temperature conditions for serpentinization^15,16,26^. This process is further supported by the occurrence of microseismicity around a high-velocity mantle body, which is interpreted as evidence of ongoing hydration^27,28^. Evaporites and claystones within the overlying sedimentary sequence may act as a trap for the hydrogen being produced^29^. Soil gas measurements (Fig. 1b) confirm the presence of hydrogen seepage in the vicinity of major thrust faults, indicating upward migration toward the surface^23^. Gravity and magnetic anomalies align with a body of variably serpentinized lithospheric mantle at an unusually shallow depth^26^.

**Fig. 2:**
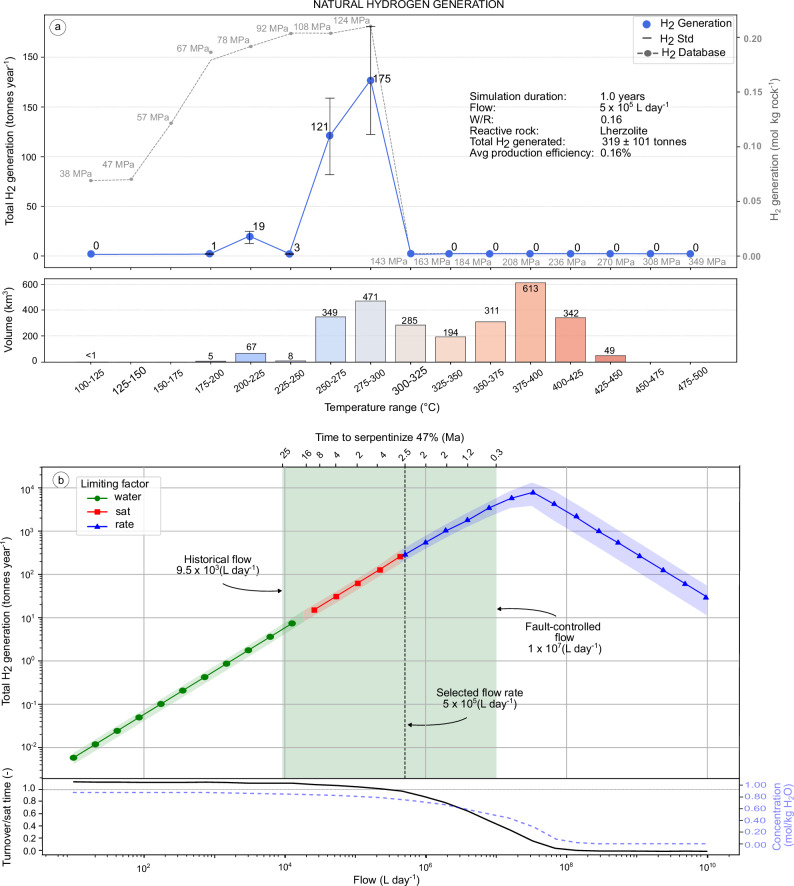
Overview of H_2_(aq) generation estimates under contrasting water availability scenarios for the Western Pyrenees. **a** Results of natural hydrogen generation and ± 1 standard deviation of the values (blue dots in the upper rectangle and black error lines), for a flow of 5 × 10^5 ^L day^−1^, showing the corresponding hydrogen yield per kilogram of rock (in grey) and the volumes of partially serpentinized rocks (lower rectangle, fresh mantle not included). Numbers next to the blue points indicate the H_2_ production in tonnes year^−1^ and numbers next to the grey points represent pressure values in MPa. For visualisation purposes, hydrogen production values were rounded to the nearest whole number. Production efficiency relative to unconstrained generation conditions (refer to Supplementary Table 3). W/R denotes the water-to-rock mass ratio. **b** Total annual hydrogen production plotted against daily water flow. Marker and colour indicate the dominant limiting factor, and the shaded envelope represents ± 1 standard deviation (Note: standard deviations are shown on a logarithmic scale, visually distorting the uncertainty envelope). Green dot for water limitation for production below 10 tonnes year^-1^, red squares for saturation of H_2_ in the fluid and blue triangles for kinetics (e.g., serpentinization front velocity). The shaded area highlights the plausible flow range, spanning from historically inferred values to those controlled by fault zone permeability. The dashed line at 5 × 10⁵ L day^−1^ marks the selected intermediate scenario. The lower panel shows the ratio between pore turnover time and saturation time (black line): values greater than 1 indicate that H₂ saturation is reached before pore-water renewal, whereas values lower than 1 indicate faster pore-water renewal. A ratio close to 1 marks conditions where both processes occur on comparable timescales. The panel also reports the average dissolved H₂ concentration (blue dashed line). The reported concentration is computed using only the water that effectively enters the reactive pore system (diffusive + fracture inflow), not the total imposed flow.

Geophysical inversion of residual isostatic Bouguer and residual reduced-to-the-pole (RTP) magnetic anomalies (Fig. 1c, d), constrained by seismic velocity models^28^, and combined with thermal modelling (Fig. 1d), indicates the presence of ~ 2700 km³ of ultramafic material serpentinized over ~ 37–57% of its volume (Fig. 1e). Of this volume, ~ 630 km³ fall within the temperature range where hydrogen generation occurs according to our thermodynamic calculations, whereas ~ 2100 km³ occur at temperatures higher than the modelled hydrogen production window (refer to H2 production database in Methods for details). These rocks are spatially associated with a second group of ~ 2400 km³ of mantle rocks characterised by a minimal serpentinization of ~ 10% (green unit in Fig. 1c and Fig. 1e, with volume limited by the 20 km depth extent of the model). Maximum H₂ production, as a function of pressure and temperature, was computed for these rocks at a fixed water-to-rock ratio of 0.16 using thermodynamic calculations for a generic lherzolite in equilibrium with pure water (Fig. 2a).

The range of water input (1 × 10^4^ to 1 × 10^7^ L day⁻¹; shaded area in Fig. 2b) was constrained on one side by the maximum transport capacity of a 50 km long fault zone, selected to match the lateral extent of the ultramafic unit, and on the other side by the flow required to serpentinize 47% of the mantle rock volume over 25 Ma, assuming time-averaged fluxes (refer to Deep water flow capacity estimation in Methods for more information). Within this range, an intermediate flow rate of 5 × 10⁵ L day⁻¹ was selected as a representative scenario. Across the full range of possible flow rates, annual hydrogen production as dissolved H₂ is estimated to span ~ 0.01–9.5 tonnes H₂ yr⁻¹ km⁻³ of reactive rock, corresponding to ~ 10–8500 tonnes H₂ yr⁻¹ when integrated over the entire reactive rock volume. For the selected intermediate scenario, production reaches ~ 0.51 ± 0.16 tonnes H₂ yr⁻¹ km⁻³ of reactive rock, equivalent to 308 ± 88 tonnes H₂ yr⁻¹ (Fig. 2a, b and Supplementary Table 5).

Additional simulations (Fig. 2b and Supplementary Table 7) were performed to assess sensitivity to water supply by varying inflow rates (10¹–10^10 ^L day⁻¹; Fig. 2b). At low inflows (< 10⁴ L day⁻¹), limited water renewal leads to large pore turnover and saturation times (> 10⁴–10⁵ days), resulting in negligible hydrogen production (< 10 tonnes H₂ yr⁻¹) constrained by water availability. At intermediate flows (~ 10⁴–10⁶ L day⁻¹), pore turnover times decrease while saturation times become shorter than turnover times, causing dissolved H₂ to reach near-solubility before pore water is renewed and suppressing further hydrogen generation due to limited product removal. At higher inflows (> 10⁶ L day⁻¹), pore turnover becomes very short, and hydrogen generation initially increases as larger fluid volumes pass through the reactive zone, reaching a broad maximum of ~ 8500 tonnes H₂ yr⁻¹ when turnover and saturation timescales become comparable (refer to Supplementary Table 7). At the highest flow rates, hydrogen generation decreases as fluids are advected through the system before substantial reaction progress can occur, with pore-water turnover rates exceeding 100% per day (i.e., the effective pore-water volume is renewed more than once per day; Fig. 2b).

### Northern California

**Fig. 3:**
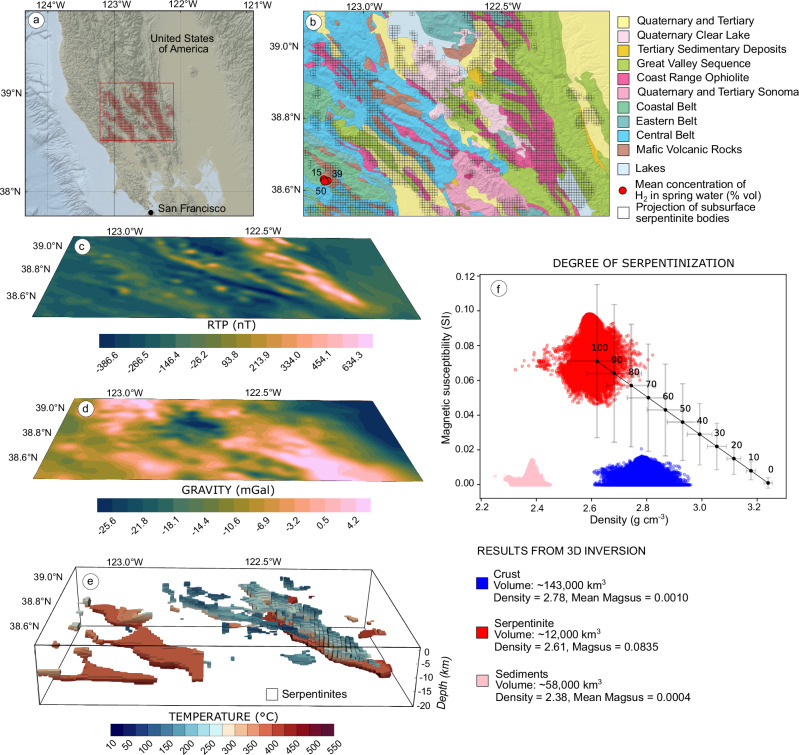
Results of source rock volumes for Northern California. **a** Index map of the study area (red box). The Red polygons show modelled serpentinite extent projected onto the surface. **b** Geologic units in the study area (modified from ref. ^36^). Polygons show modelled serpentinite blocks projected onto the surface. Red circles represent H_2_ measurements (%) in spring water (modified from ref. ^38^). **c** Reduced to the pole magnetic anomalies (RTP) for the area (modified from ref. ^36^). **d** Residual gravity anomalies for the area (modified from ref. ^36^). **e** 3D blocks showing temperature distribution for the serpentinites. **f** Results of the Gaussian Mixture Model (GMM), indicating the degree of serpentinization based on density and magnetic susceptibility results. Values are projected to the closest serpentinization point in the line. Crustal rocks and sediments represent averaged petrophysical descriptions of the heterogeneous crustal assemblage and the sedimentary cover included in the model (e.g., metasedimentary and metavolcanic rocks, greywackes, cherts, serpentinites and locally mafic rocks; unconsolidated to weakly lithified basin-fill and forearc sedimentary deposits).

The north-western part of the United States of America, north of San Francisco (Fig. 3a, b), hosts extensive, shallow harzburgite-rich ophiolite complexes^19^. These include the Coast Range ophiolite, which likely originated in a supra-subduction zone setting and was emplaced onto continental crust during Late Jurassic tectonic events^30,31^. Their present crustal position reflects not only Jurassic emplacement but also major Cretaceous tectonic imbrication and uplift since approximately 90 Ma^32^. The presence of coarse, unfoliated bastite (orthopyroxene pseudomorphs) in some areas, however, indicates that strong tectonic overprinting or deep burial did not occur pervasively throughout the complex^33^. Great Valley forearc data indicate that Jurassic-Cretaceous syn-sedimentary extension, followed by Cenozoic folding, uplift, and strike-slip faulting linked to the shift from subduction to a transform boundary, reshaped these structures^34,35^.

**Fig. 4:**
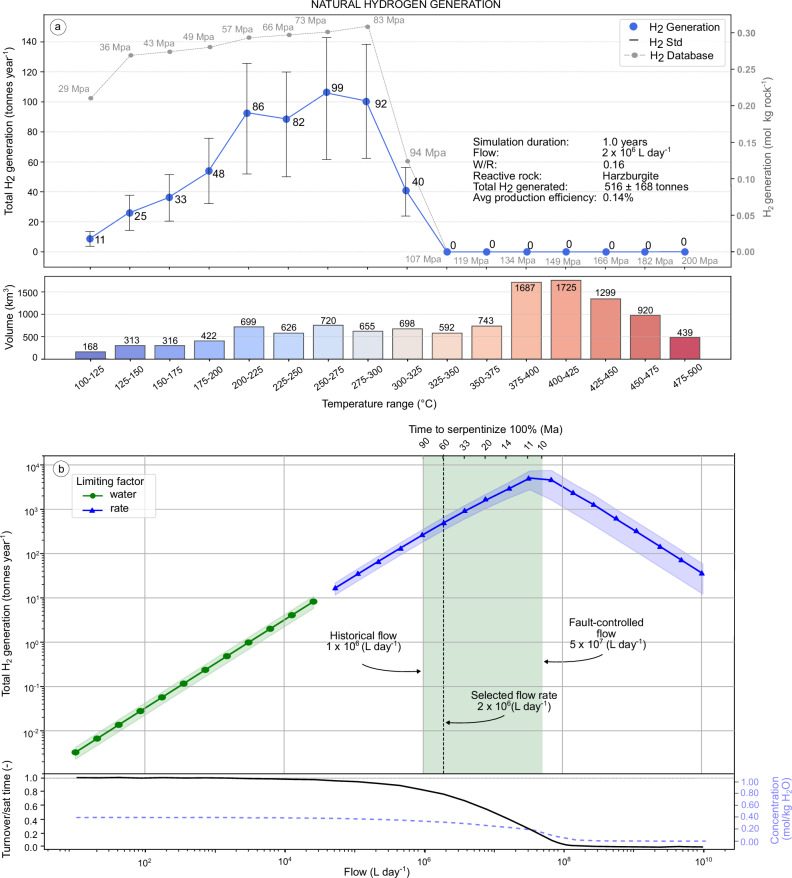
Overview of H_2_(aq) generation estimates under contrasting water availability scenarios for Northern California. **a** Results of natural hydrogen generation and ± 1 standard deviation of the values (blue dots in the upper rectangle and black error lines), for a flow of 2 × 106 L day^−1^, showing the corresponding hydrogen yield per kilogram of rock (in grey) and the volumes of partially serpentinized rocks (lower rectangle). Numbers next to the blue points indicate the H_2_ production in tonnes year^−1^ and numbers next to the grey points represent pressure values in MPa. For visualisation purposes, hydrogen production values were rounded to the nearest whole number. Production efficiency relative to unconstrained generation conditions (refer to Supplementary Table 4). W/R denotes the water-to-rock mass ratio. **b** Total annual hydrogen production plotted against daily water flow. Marker and colour indicate the dominant limiting factor, and the shaded envelope represents ± 1 standard deviation (Note: standard deviations are shown on a logarithmic scale, visually distorting the uncertainty envelope). Green dots for water limitation for production below 10 tonnes year^−1^ and blue triangles for kinetics (e.g., serpentinization front velocity). The shaded area highlights the plausible flow range, spanning from historically inferred values to those controlled by fault zone permeability. The dashed line at 2 × 106 L day^_1^ marks the selected intermediate scenario. The lower panel shows the ratio between pore turnover time and saturation time (black line): values greater than 1 indicate that H₂ saturation is reached before pore-water renewal, whereas values lower than 1 indicate faster pore-water renewal. A ratio close to 1 marks conditions where both processes occur on comparable timescales. The panel also reports the average dissolved H₂ concentration (blue dashed line). The reported concentration is computed using only the water that effectively enters the reactive pore system (diffusive + fracture inflow), not the total imposed flow.

Currently, these complexes are at depths where temperatures and active fault systems are favourable for serpentinization, with faults providing permeable pathways for fluids to hydrate the ultramafic rocks^19^. Large-amplitude positive magnetic anomalies (Fig. 3c) align with the serpentinite bodies mapped in the region, sometimes acting as a proxy to infer zones of aseismic slip^36^. In contrast, gravity highs (Fig. 3d) reflect the presence of relatively dense crustal units beneath thinner sedimentary cover. Surface measurements showing elevated hydrogen concentrations (Fig. 3b) and thus ongoing serpentinization processes, suggest active fluid migration from depth^37,38^. Geophysical inversion of residual isostatic Bouguer and residual reduced to the pole magnetic anomalies, guided by the lithospheric geometry derived from P-wave tomographic models^39^, and integrated with thermal modelling (Fig. 3c, d), reveals the presence of ~ 12,000 km³ of highly serpentinized rocks. Of this volume, ~ 4500 km³ fall within the temperature range predicted by our thermodynamic framework to sustain hydrogen generation (refer to H2 production database in Methods for details). In addition, ~ 7500 km³ of ultramafic rocks lie outside this hydrogen-producing temperature window. Results also suggest that the degree of serpentinization of the mantle rocks in Northern California is very high, with average values approaching complete serpentinization (Fig. 3f). Maximum H₂ production as a function of pressure and temperature was computed for these rocks at a fixed water-to-rock ratio of 0.16 using thermodynamic calculations for a generic harzburgite in equilibrium with pure water (Fig. 4a).

The expected range of fluid input (~ 10⁶ to ~ 5 × 10⁷ L day⁻¹; highlighted in Fig. 4b) combines constraints from two independent considerations. The upper bound reflects the estimated maximum water-delivery capacity of a 250 km long fault system, consistent with the mapped extent of the ultramafic units (refer to Deep water flow capacity estimation in Methods for more information). In contrast, the lower bound represents the minimum sustained, time-averaged fluid flux required to achieve near-complete serpentinization over a~90 Ma timescale, assuming continuous fluid circulation. An intermediate reference flow rate of 2 × 10⁶ L day⁻¹ was selected to represent present-day fluid circulation conditions. Across this plausible range of flow rates, annual hydrogen production as dissolved H₂ spans ~ 0.11–1.11 tonnes H₂ yr⁻¹ km⁻³ of reactive rock, corresponding to ~ 500–5000 tonnes H₂ yr⁻¹, when integrated over the entire reactive volume. For the intermediate flow scenario, production is estimated at ~ 0.11 ± 0.03 tonnes H₂ yr⁻¹ km⁻³, equivalent to 515 ± 168 tonnes H₂ yr⁻¹ (Fig. 4a, b and Supplementary Table 6).

Sensitivity to water supply was tested by varying inflow rates (10¹–10^9^ L day⁻¹; Fig. 4b and Supplementary Table 8) at fixed water-to-rock ratio (0.16). At very low inflow rates (< 10⁴ L day⁻¹), fluid renewal within the reactive volume is negligible, resulting in long residence times and hydrogen production remaining below ~ 10 tonnes yr⁻¹. With increasing inflow, fluid circulation becomes progressively more efficient, allowing hydrogen to be transported away from the reaction sites and leading to an increase in production. At intermediate to high inflow rates (≳ 10⁵–10⁷ L day⁻¹), hydrogen generation is no longer limited by water availability but becomes controlled by reaction kinetics. Under these conditions, production reaches a broad maximum of ~ 5000 tonnes H₂ yr⁻¹, before declining at the highest flow rates, as rapid pore-water renewal limits fluid residence time and prevents substantial hydrogen generation.

### Model sensitivity

Univariate sensitivity analyses based on 5,000 Monte Carlo realisations per parameter for both the unconstrained and constrained hydrogen generation models (refer to Supplementary Tables 9 and 10) yield consistent sensitivity patterns. The unconstrained (no-saturation) model shows strong and nearly linear sensitivity to multiple parameters that control hydrogen generation. Variations in serpentinization front velocity ( ± 60% of baseline) produce changes of ~ 120% relative to base output, while intrinsic production rate ( ± 50%), effective reactive surface area ( ± 40%), reactive volume ( ± 20%), and serpentinization correction ( ± 20%) result in changes of ~ 100%, ~ 80%, ~ 40% and ~ 40%, respectively. In contrast, in the constrained model, only parameters directly controlling hydrogen saturation in the fluid influence the amount of hydrogen finally generated. Varying mean pressure and hydrogen solubility within ± 30% of their baseline values results in changes of ~ 57–58% relative to base output. All other parameters, including reactive volume (± 20%), serpentinization degree (± 20%), fracture spacing (± 50%), permeability (± 20%), intrinsic production rate (± 20%), block size (± 50%), and reactive rock mass (± 60%), produce negligible effects on total hydrogen output over their tested ranges.

## Discussion

**Fig. 5:**
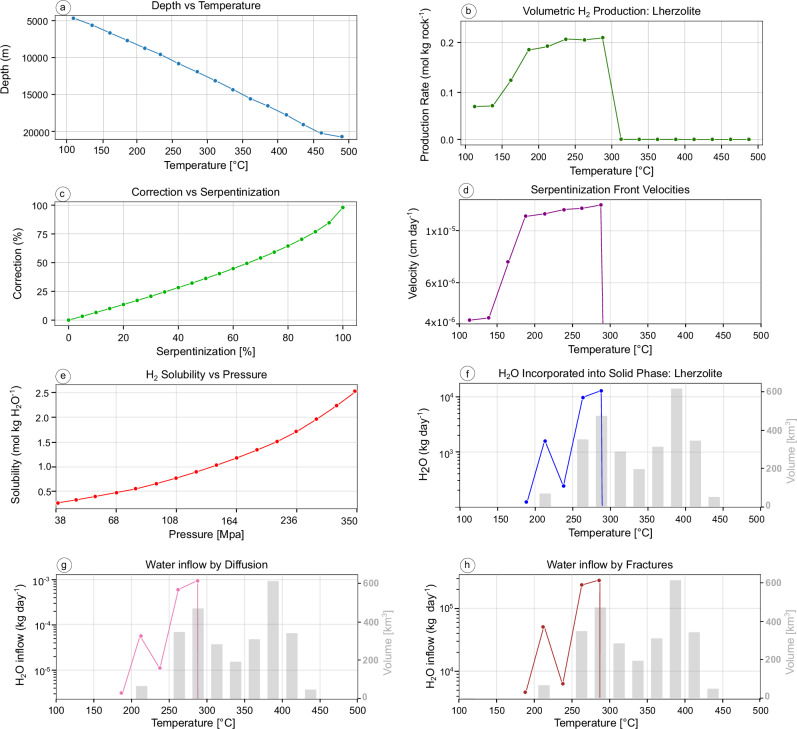
Thermodynamic and kinetic parameters of serpentinization modelled for the studied pressure and temperature (P-T) conditions in the Western Pyrenees. **a** Modelled temperature profile as a function of depth. **b** Volumetric hydrogen production rates (mol H₂ kg rock^−1^) estimated for each temperature interval based on thermodynamic modelling for lherzolite. **c** Empirical correction factors as a function of serpentinization degree. **d** Estimated serpentinization front velocities. Non-existent values reflect conditions where serpentinization is not active. **e** Solubility of molecular hydrogen in water. **f** Amount of water incorporated into the solid phase during serpentinization. Bars indicate the volumes of reactive rocks. **g** Modelled water inflow rates by molecular diffusion across the serpentinized shell. **h** Estimated water inflow rates across the serpentinized shell through fracture-controlled advection, based on fracture permeability models.

Our three-dimensional models of the Western Pyrenees (Fig. 1c, d) indicate that exhumed mantle rocks extend from depths of ~ 8 km to at least 20 km, corresponding to the lower limit of our model. These ultramafic rocks can be subdivided into a deeper domain characterised by a low degree of serpentinization (~ 10%), and a shallower domain that exhibits substantially higher alteration levels (~ 37–57%). This subdivision is largely consistent with the temperature window over which our thermodynamic calculations predict hydrogen-producing serpentinization (Fig. 5b), and aligns with previous interpretations based on gravity, magnetic, and P-wave velocity models^26,28^, as well as petrophysical modelling^16^. In this area, we estimated a natural hydrogen generation, under the most likely fluid flow conditions inferred for the study area, on the order of a few hundred tonnes of H₂ per year, corresponding to a normalised production rate of ~ 0.51 ± 0.16 tonnes H₂ yr⁻¹ km⁻³ of reactive rock. Under these settings, dissolved H₂ accumulates to near-saturation levels before pore-water renewal occurs, progressively reducing the thermodynamic driving force for further hydrogen generation. In contrast to our findings, previous studies have estimated a theoretical hydrogen production potential ranging from several hundred thousand^15^ to several millions of tonnes per year^16^. Because these estimates are based solely on the volume of reactive rocks and do not account for water availability or fluid saturation, they are only comparable to the results obtained with our unconstrained model (refer to Supplementary Tables 3 and 4).

For Northern California, our estimations also indicate representative long-term hydrogen generation on the order of a few hundred tonnes of H₂ per year, as evaluated under flow conditions defined within the explored parameter space. When normalised to the volume of reactive rock, this corresponds to a production rate of ~ 0.11 ± 0.03 tonnes H₂ yr⁻¹ km⁻³ of reactive rock. In contrast to the Western Pyrenees, hydrogen generation in this system is primarily limited by reaction kinetics, which reflects the near-complete serpentinization of the mantle peridotites. Seismic velocity models^39^, together with surface observations of serpentinites and two-dimensional magnetic modelling^36^, consistently indicate widespread and advanced alteration of the ultramafic rocks. As a result, the volume of remaining reactive material is comparatively limited, but extensive fracturing maintains a large effective pore space, allowing a high fluid circulation that prevents rapid saturation of the reacting fluids with dissolved hydrogen. Therefore, generation is controlled mainly by the rate at which new reactive surfaces and porosity can be generated, with fluid saturation playing only a secondary role.

Interpretation of these results requires consideration of several first-order simplifications inherent to the modelling approach. Fluid-rock reactions are represented as a sequence of local thermodynamic equilibrium steps, such that short-lived kinetic limitations and fine-scale spatial heterogeneities are not explicitly resolved. Hydrogen production is therefore estimated as a quasi-dynamic, time-averaged rate rather than as instantaneous or peak fluxes. Moreover, thermodynamic calculations consider only H₂ generation associated with magnetite formation and assume pure water as the reacting fluid, although experimental studies indicate that increased salinity and reduced water activity can significantly slow serpentinization reaction rates^4^. In addition, fluids are assumed to be uniformly distributed throughout the reactive rock volume, whereas natural systems exhibit highly heterogeneous, fracture-oriented fluid access. These assumptions have counteracting effects on predicted hydrogen generation: pure-water conditions and homogeneous fluid access systematically bias hydrogen production toward higher values, while limiting H₂ generation to magnetite-forming reactions alone biases estimates toward lower total yields. Consequently, the derived production rates should be interpreted as first-order magnitudes rather than precise in-situ values. Nevertheless, our average production estimates of ~ 0.31 ± 0.08 tonnes H₂ yr⁻¹ km⁻³ of reactive rock fall within the range of natural hydrogen fluxes inferred from other crustal settings, including the Bulqizë ophiolite (Albania) and the Zambales ophiolite (Philippines), which correspond to normalised values of ~ 0.2 and ~ 1.4 tonnes H₂ yr⁻¹ km⁻³, respectively^13,14,40^. Although seep fluxes are not direct proxies for generation rates, this agreement suggests that the model captures the correct order of magnitude of natural hydrogen generation systems.

The present scenario likely does not apply to the earliest stages of serpentinization. While water may have been available, the initially low degree of fracturing in fresh ultramafic rocks would have limited fluid access and reactive surface area, and this would have resulted in weak fluid-rock interaction and low hydrogen production. As serpentinization progresses, together with tectonic deformation and reaction-induced cracking, fracture density and permeability increase, creating additional fluid pathways and exposing progressively larger reactive surfaces to fluids. This is consistent with laboratory evidence, which indicates that fracture-controlled flow is the dominant mechanism for fluid delivery in ultramafic settings^41^. This structural and chemical evolution likely led to a gradual acceleration of serpentinization and associated hydrogen generation. However, maintaining such conditions continuously would imply complete hydration of the ultramafic volume well within the time elapsed since mantle exhumation. The modelled serpentinization rates should therefore be interpreted as effective rates representative of periods with sustained fluid access and enhanced fracturing^42^. In natural systems, fluid supply and permeability are expected to be intermittent and spatially localised, so elevated reaction rates are likely to occur only during short-lived, fracture-controlled fluid-infiltration events, separated by longer periods of limited or no reaction.

In our modelling framework, hydrogen is generated and transported exclusively in the aqueous phase, and the formation, migration and trapping of a free gas phase are not explicitly simulated. Partial exsolution may occur during upward migration, as pressure decreases and hydrogen solubility is reduced, but considerable accumulations are expected only where favourable structural conditions exist, such as effective traps and low-permeability seals. Higher fluid fluxes do not necessarily result in larger accumulations, as increased dilution in the aqueous phase can delay gas-phase separation and long-term retention. In line with recent play-based assessments of natural hydrogen systems^43^, hydrogen prospectivity reflects the efficiency of migration, phase separation, and trapping rather than generation rates alone. The production levels estimated here fall below thresholds typically required for large industrial offtake and are therefore compatible with localised applications unless exceptional trapping conditions permit gas-phase accumulation. This conclusion does not rule out other geologic sources or processes that may operate over shorter timescales and could sustain higher production rates. Integrating hydrogen generation models with reactive transport and migration simulations would provide a more quantitative basis to evaluate the potential for phase separation and long-term retention, thereby better constraining the viability of subsurface hydrogen accumulations.

## Methods

### Subsurface modelling and petrophysical constraints

Initial three-dimensional models of the primary subsurface rock units (e.g., mantle rocks, serpentinized rocks, basement rocks) are constructed based on 2D sections. These initial models are not intended to represent detailed geological structures and serve only as initial guides to help the inversion converge toward geologically plausible solutions. Petrophysical data are parametrised from seismic velocity models (refer to Supplementary Tables 1 and 2). Observed gravity and magnetic data are jointly inverted for density and magnetic susceptibility using a Petrophysically Guided Inversion (PGI) framework implemented in SimPEG^44,45^ in which petrophysical information is incorporated through Gaussian mixture models linking lithological classes to admissible ranges of physical properties. For both study areas, density and magnetic susceptibility are constrained within physically plausible bounds, while initial models and background values are used only to initialise the inversion.

The inversion is formulated as a regularised optimisation problem in which density and magnetic susceptibility are the control variables, and the objective function minimises the misfit between observed and predicted gravity and magnetic anomalies while enforcing petrophysical consistency and spatial smoothness. Regularisation assumes laterally and vertically coherent property distributions and penalises strong gradients in the three spatial directions through first- and second-order smoothness terms. The trade-off between data misfit and regularisation is controlled by an iterative cooling schedule that progressively relaxes regularisation during the inversion. Physical bounds are imposed on the recovered properties depending on lithology. Resulting cells consistent with serpentinized rocks are identified based on their joint density-susceptibility values within the PGI framework (Figs. 1e and 3f), and the volumes of these units are subsequently quantified. A brief description of the inversion parameters used for each case study is provided in Supplementary Tables 1 and 2, and the full set of parameters used in the numerical experiments can be found in the configuration files made available in the Data Availability section.

### Thermal modelling

**Fig. 6:**
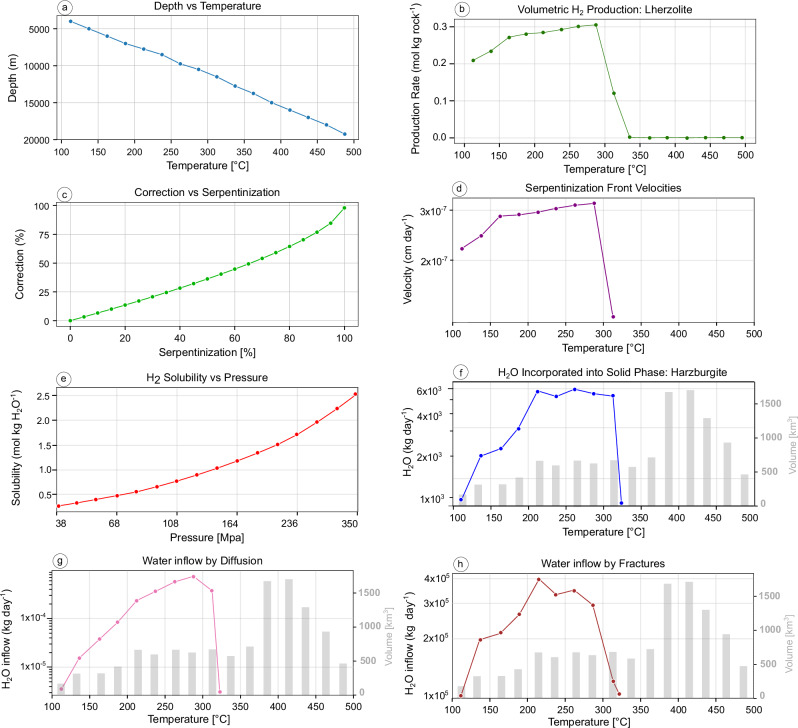
Thermodynamic and kinetic parameters of serpentinization modelled for the studied pressure and temperature (P-T) conditions in Northern California. **a** Modelled temperature profile as a function of depth. **b** Volumetric hydrogen production rates (mol H₂ kg rock^−1^) estimated for each temperature interval based on thermodynamic modelling for harzburgite. **c** Empirical correction factors as a function of serpentinization degree. **d** Estimated serpentinization front velocities. Non-existent values reflect conditions where serpentinization is not active. **e** Solubility of molecular hydrogen in water. **f** Amount of water incorporated into the solid phase during serpentinization. Bars indicate the volumes or reactive rocks. **g** Modelled water inflow rates by molecular diffusion across the serpentinized shell. h) Estimated water inflow rates across the serpentinized shell through fracture-controlled advection, based on fracture permeability models.

Subsurface temperature distributions (Figs. 5a and 6a) are calculated by solving the steady-state heat conduction equation using the finite volume method, as implemented in the FiPy package^46^. At each depth interval, thermal properties are simulated using piecewise-linear functions that represent the natural variability of the thermal conductivity with depth. A fixed temperature at the surface (i.e., Dirichlet boundary condition), and a background heat flow at the base (i.e., Neumann boundary condition) are imposed (refer to Supplementary Tables 1 and 2). The previously identified volumes of partially serpentinized rocks are populated with the temperature distribution. Although ultramafic rocks are generally considered capable of generating hydrogen between 100 and 500 °C, only a narrower temperature interval is relevant here, as H₂ production is restricted to thermodynamically stable magnetite-forming reactions (refer to H_2_ production database).

### H_2_ production database

Thermodynamic equilibrium calculations for hydrogen production were compiled for two representative ultramafic lithologies reacting with pure water: lherzolite (Al₂O₃ 4.46, CaO 2.65, FeO 7.86, MgO 40.13, SiO₂ 44.75 wt%) and harzburgite (Al₂O₃ 3.13, CaO 1.25, FeO 8.69, MgO 43.53, SiO₂ 43.33 wt%). Potential H₂ production reaches around 250 mmol kg rock^−1^ for lherzolite and 300 mmol kg rock^−1^ for harzburgite (Figs. 5b and 6b). These thermodynamic equilibrium results are taken directly from ref. ^47^ and were used as input constraints in this study. The original calculations were performed using Gibbs free energy minimisation with the Theriak-Domino software^48^. In these, the thermodynamic databases JUN92d.bs^49^ and TC-DS633^50,51^ were evaluated. Both resulted in similar H2 production rates at equilibrium for the evaluated bulk compositions, and the calculations from JUN92d.bs were finally applied. However, none of these databases included recent solid solution models for Fe(III)-bearing serpentines^52^, such that hydrogen generation is attributed only to Fe(III) iron allocated to magnetite formation. The water-to-rock ratio (W/R) considered in the database spans 0.0–0.17, beyond which no further metamorphic transformation occurs and hydrogen production remains unchanged.

### Serpentinization framework

A correction was applied to account for the loss in reactive surface due to the progressive volume replacement of peridotite by serpentinite (Figs. 5c and 6c), assuming serpentinization progresses inward from the periphery of each block and accounting for unaltered olivine cores. As a result, the hydrogen generation potential of each block is reduced compared to an entirely unaltered peridotite volume, since only the remaining unaltered fraction is available for ongoing serpentinization reactions. The volumetric serpentinization degree is estimated by comparing the average density and magnetic susceptibility weighted by the proportions of altered and unaltered material to reference datasets^7–9,53,54^. To conservatively account for isolated or kinetically-inhibited volumes resistant to complete alteration at the core^55,56^, a residual value of 2% is adopted in this study.

### Fracture network and serpentinization front velocities

Reactive rock blocks with sub-metre to metre-scale dimensions are defined based on fracture spacing observed in outcrops^57–61^. To estimate the progression velocities of the serpentinization front across temperature ranges (Figs. 5d and 6d), we adopt a scaling approach based on hydrogen production rates. A reference temperature-pressure condition with a known front velocity (e.g., ref. ^42^) is first selected. The serpentinization front propagation velocity at temperature T and pressure P, is then estimated by scaling the reference velocity according to the relative hydrogen production rate

1 $${v}_{f}\left(T,P\right)={v}_{{ref}{{\rm{\cdot }}}}\frac{{R}_{H2}\left(T,P\right)}{{R}_{H2}^{{ref}}\left({T}_{r},{P}_{r}\right)}$$

where ${v}_{{ref}}$ is a reference velocity, ${R}_{H2}\left(T,P\right)$ is the hydrogen production rate at those conditions, ${R}_{H2}^{{ref}}\left({T}_{r},{P}_{r}\right)\,$ is the rate at reference conditions. These representative reactive blocks are then replicated throughout the modelled volume according to their temperature range, allowing locally-derived front velocities to be volumetrically integrated across the full three-dimensional reactive domain.

### Estimation of H₂ solubility

Hydrogen solubility in pure water (Figs. 5e, 6e) was evaluated using the Krichevsky-Kasarnovsky (KK) relation with fugacity corrections to account for non-ideal gas behaviour. Solubility, expressed as molality *m*(mol kg⁻¹ H₂O), is given by the equation

2 $$m\left(T,P\right)={m}_{0}(T)\left(\frac{\phi \left(T,P\right)P}{{P}_{0}}\right)\exp \left[-\frac{{V}_{\infty }(P-{P}_{0})}{{RT}}\right]$$

where T and P are temperature and pressure, ${P}_{0}=0.1$ MPa is the reference pressure, *R* is the universal gas constant, $\phi \left(T,P\right)$ is the fugacity coefficient of H₂, and ${V}_{\infty }$ is the partial molar volume of dissolved hydrogen at infinite dilution. A constant value ${V}_{\infty }$ = 1.7 × 10^−5^ m^3^ mol^-1^ was adopted, consistent with experimentally-constrained values reported in ref. ^62^. Fugacity coefficients were computed using the Peng-Robinson equation of state for pure H₂, with critical properties ${T}_{c}$ = 33.19 K, ${P}_{c}$ = 1.293 MPa. Rather than prescribing an explicit temperature-dependent reference solubility, the reference molality ${m}_{0}(T)$ was inferred directly from the experimental dataset of ref. ^62^. For each isotherm, ${m}_{0}(T)$ was obtained by rearranging the KK expression for all available experimental data points $(P,m)$, and a representative value was determined by averaging the inferred values at fixed temperature. The inferred values of ${m}_{0}(T)$ range between ~ 7 × 10^-4^ and 1 × 10^−3^ mol kg⁻¹ over the investigated temperature range.

### Hydrogen production estimation

Hydrogen generation is first estimated under unconstrained conditions, where neither kinetic limitations nor hydrogen solubility and fluid saturation are considered (refer to the Supplementary for a more detailed mathematical explanation). For each temperature and pressure condition, the theoretical hydrogen generation is calculated as

3 $${n}_{H2}^{{unconstrained}}\left(T,P\right)={\rho }_{{rock}}\cdot {Y}_{H2}^{{eq}}(T,P)\cdot {V}_{{front}}\left(T,\,P\right)$$

where ${\rho }_{{rock}}$ is the rock density and ${Y}_{H2}^{{eq}}$ is the equilibrium H₂ yield (i.e., the maximum amount of H₂ that can be produced per unit mass of rock) derived from thermodynamic equilibrium calculations and ${V}_{{front}}$ is the rate of advancement of the reactive volume. These values define the theoretical upper limit of the amount of hydrogen that can be produced, as determined by the intrinsic properties of the rock and the extent of serpentinization. The corresponding theoretical maximum production rate is obtained only when this equilibrium hydrogen yield is coupled to fluid flow, which controls renovation of the water and the rate at which reactive volumes are accessed.

In our models, serpentinization is associated with changes in porosity that locally enhance water inflow at the serpentinization front^63^. However, the net porosity evolution is uncertain, as fracture sealing by serpentine precipitation competes with fracture generation induced by volumetric expansion, and saturation effects progressively limit hydrogen release. A fixed daily water flow is imposed and distributed across temperature intervals in proportion to their reactive volume, ensuring preferential allocation to active zones (i.e., zones with higher fracture density and permeability). Complete mixing between incoming fluids and resident pore water is assumed, such that high inflow rates shorten fluid residence times in the pores and limit water-rock interaction. Temperature intervals that do not generate H₂ according to the thermodynamic calculations are treated as hydraulically closed and do not receive water, even if reactive rock is present. Within each interval, water is further partitioned between water that is incorporated into the rock, i.e., diffusive inflow and fracture-controlled flow (Figs. 5f–h, 6f–h). These contributions are governed by Fick’s law for diffusive transport and by Darcy-type (cubic-law) flow for fracture-controlled transport. Hydrogen solubility, expressed as molality *m* (mol kg⁻¹ H₂O), is given by Eq. (2) and is hereafter denoted ${S}_{\max }\left(T,P\right)$. The maximum amount of hydrogen that the available water can retain under in-situ pressure conditions is then

4 $${n}_{H2}^{\max }\left(T,P\right)={S}_{\max }\left(T,P\right)\cdot {M}_{H2O}\left(T,P\right)$$

where ${S}_{\max }\left(T,P\right)\,$ is the H_2_ solubility in water and ${M}_{H2O}$ includes both water filling newly formed porosity and inflow from diffusion and fractures. The model tracks the cumulative amount of hydrogen already dissolved in the water ${{n}}_{H2}^{{dissolved}}$. This is used to update the saturation ratio, which limits further production

5 $${S}_{H2}^{{eq}}\left({{\rm{T}}},{{\rm{P}}}\right)=\frac{{n}_{H2}^{{dissolved}}\left({{\rm{T}}},{{\rm{P}}}\right)}{{n}_{H2}^{\max }\left(T,P\right)}$$

As saturation increases, less hydrogen can be retained in the aqueous phase. The effective hydrogen generation is thus scaled by the fraction of unsaturated capacity

6 $${n}_{H2}^{{cell}}\left({{\rm{T}}},{{\rm{P}}}\right)={n}_{H2}^{{unconstrained}}\left(T,P\right)\cdot \left[1-{S}_{H2}^{{eq}}\left({{\rm{T}}},{{\rm{P}}}\right)\right]$$

**Fig. 7:**
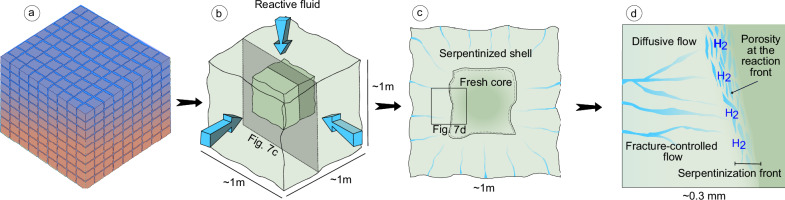
Conceptual representation of the serpentinization-front architecture and associated fluid transport mechanisms. **a** The model domain is discretised into cubic elements for integration with the coupled thermodynamic and kinetic framework. **b** The reactive fluid enters each ~ 1 m block through external boundaries (fracture system), simulating infiltration across fracture-controlled pathways and diffusive gradients. **c** Progressive alteration results in the formation of a serpentinized shell enclosing a progressively-shrinking fresh ultramafic core. **d** At the serpentinization front, hydrogen (H₂) is generated as unaltered minerals react with the infiltrating fluid. Access of the reactive fluid to the alteration front is governed by two transport processes: (i) diffusive flow across the low-permeability serpentinized shell, parameterised as a function of temperature and pressure, and (ii) fracture-controlled flow, controlled by fracture network permeability and pressure gradients. The evolving porosity at the reaction front regulates both fluid accessibility and reaction progression.

All the above calculations are carried out for a single representative reactive block within each temperature interval (Fig. 7). The resulting hydrogen production is then extrapolated to the full reactive domain by multiplying by the number of such blocks present within the corresponding temperature range. The total hydrogen production is finally obtained by summing these block-scaled contributions over all temperature intervals and over the simulation period (typically one year).

### Deep water flow capacity estimation

**Fig. 8:**
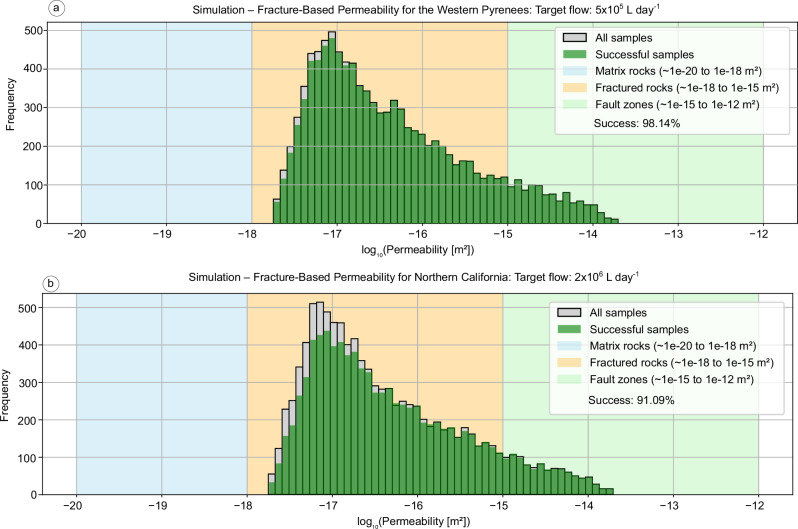
Permeability distributions and hydraulic success for water delivery into serpentinizing mantle rocks. **a** Western Pyrenees: simulations for a target deep water flux of 5 × 10⁵ L day⁻¹. **b** Northern California: simulations for a target deep water flux of 2 × 10⁶ L day⁻¹. The histograms show the full ensemble of sampled fracture permeabilities (grey) and the subset of realisations that meet or exceed the target deep water flux (green). Shaded background regions indicate approximate permeability ranges for matrix rocks (blue: ~ 10⁻²⁰–10⁻¹⁸ m²), fractured rocks (orange: ~ 10⁻¹⁸–10⁻¹⁵ m²), and fault zones (green: ~ 10⁻¹⁵–10⁻¹² m²). Simulations consider stochastic variability in fracture network geometry, depth, fluid pressure, viscosity, and hydraulic connectivity.

A Monte Carlo approach was used to test the deep-water flow potential under different fracture network configurations. Although the analysed faults have the capacity to transport the volumes of water used in our calculations, the potential source of this water has not been established. Flow is calculated as a function of permeability, fracture area, depth, pressure gradient, and fluid viscosity using Darcy’s law. Fracture network geometries were generated by sampling these key parameters across physically plausible ranges. Maps of ultramafic bodies at depth were used to extract fault segments, while damage zone thickness was randomly sampled within the 1–1000 m range to reflect the variability in fracture network widths^64^. In these zones, only a fraction of the fault length was assumed to be hydraulically active (5–30%), reflecting the strong along-strike heterogeneity of fault-zone architecture and permeability^65,66^. Fracture density was assumed to decrease with depth, consistent with increasing effective stress and reduced fracture connectivity at depth. The total fracture wall area available for flow was computed as a function of fracture count, fault length, and vertical extent. Effective permeability was sampled based on an empirical depth-dependent trend^67^. A hydraulic connection fraction of 10–25% was applied to simulate the partially connected nature of fracture networks within fault damage zones, consistent with structural models indicating heterogeneous and discontinuous fluid pathways^65,66^. This range was selected based on sensitivity testing. Pressure differentials driving flow were sampled between 60 and 140 MPa, representing lithostatic-hydrostatic contrasts at depths ranging from 1 to 12 km. Water viscosity was varied between 1 × 10⁻³ and 1 ×10⁻⁵ Pa·s, thus reflecting temperatures from near-surface to ~ 600 °C. For each realisation, permeability and all other variables were combined to estimate the resulting flow rates. Success was defined as achieving or exceeding a pre-defined target flux inL day^−1^, selected to represent a geologically plausible range. The simulations quantified success rates and identified flow rates sustainable in at least 70% of cases (Fig. 8). The geologically plausible range of deep-water fluxes considered in this study (green-shaded domains in Figs. 2 and 4) was explicitly tested using this Monte Carlo framework and was selected to correspond to flow conditions that can be sustained in at least 70% of the realisations.

### Limiting-factor classification

For each temperature interval, the limiting factor of H₂ generation is diagnosed *a posteriori* from the simulated response. Intervals with zero H₂ production are classified as inactive, whereas non-zero but negligible production (< 10 tonnes year^−1^) is treated as water-limited, reflecting insufficient fluid supply. For productive cases, dissolved H₂ accumulation relative to solubility is quantified by comparing two diagnostic timescales: the pore turnover time (${\tau }_{{turn}}$), defined as the time required to renew the pore-water volume, and the saturation time (${\tau }_{{sat}}$), defined as the characteristic time required for dissolved H₂ to accumulate to near-solubility concentrations. Hydrogen generation is classified as rate-limited when ${\tau }_{{sat}} > \,{\tau }_{{turn}}\,$ and as solubility-limited when ${\tau }_{{sat}} < \,{\tau }_{{turn}}$.

### Uncertainty quantification and sensitivity analysis

Hydrogen production uncertainties were estimated in a Monte Carlo framework consistently for both unconstrained and constrained models. Scaling factors were applied to key parameters (such as pressure, hydrogen solubility, reaction rate, serpentinization-front velocity, reactive surface area and reactive volume) to represent model uncertainty. Each factor was sampled over a prescribed range about its reference value, representing natural variability for the physical, thermodynamic and geometric properties. The resulting ensembles were used to compute mean values and dispersion metrics (e.g., standard deviation) of total hydrogen generation under each modelling scenario. To further isolate the contribution of individual parameters to overall uncertainty, a univariate sensitivity analysis was performed. Each parameter was varied across the prescribed range while all other parameters were kept at reference values. At each sampling point, multiple Monte Carlo realisations were carried out to capture stochastic variability. A baseline simulation, with all scaling factors set to unity, was used as a reference state for normalisation.

## Supplementary information

Transparent Peer Review file

Supplementary infromation

## Data Availability

All data supporting the findings of this study are available via Zenodo at 10.5281/zenodo.18733249.

## Code availability

The PoNHy (Potential for Natural Hydrogen) source code used in this study is archived on Zenodo at 10.5281/zenodo.18733249. The actively developed version, including the latest updates and improvements, is available on GitHub at https://github.com/RodolfoChristiansen/PoNHy.
