# Supplementary material for: Controls on natural hydrogen generation during serpentinization of mantle rocks
Source: Nat Commun. 2026 Jun 11;17:5211. doi: 10.1038/s41467-026-73920-5 (PMC13260452; doi:10.1038/s41467-026-73920-5)
Supplement: Supplementary file 2 — Supplementary infromation [file 41467_2026_73920_MOESM2_ESM.pdf]

Controls on natural hydrogen generation during serpentinization of mantle rocks

Rodolfo Christiansen[1,9]*, Mohamed Sobh[1], Christian Ostertag-Henning[2], Guido Gianni[3], Nicolas Saspiturry[4], Sebastien Chevrot[5], Victoria Langenheim[6], Javier García-Pintado[7], Gerald Gabriel[1,8]

[1] LIAG Institute for Applied Geophysics, 30655 Hannover, Germany.
[2] Federal Institute for Geosciences and Natural Resources (BGR), 30655 Hannover, Germany.
[3] Institute of Geophysics, Czech Academy of Sciences, 14100 Prague, Czechia.
[4] Géosciences Montpellier, Université de Montpellier, CNRS, 34095 Montpellier, France.
[5] Géosciences Environnement Toulouse (GET), UMR 5563, Observatoire Midi-Pyrénées, CNRS, Université de Toulouse, 31400 Toulouse, France.
[6] United States Geological Survey (USGS), Moffett Field, California 94035, USA.
[7] MARUM - Center for Marine Environmental Sciences, University of Bremen, 28359 Bremen, Germany.
[8] Institute of Earth System Sciences, Leibniz University Hannover, 30167 Hannover, Germany.
[9] Present address: Mantle8, 38360 Sassenage, France

*Corresponding author email: r.christiansen@mantle8.com

Mathematical and Physical Description of the Methodology

1. Temperature distribution. The model solves the steady-state heat conduction equation in one dimension

$$\frac{d}{dz}\left[-\lambda(z)\frac{dT}{dz}\right] = 0 \tag{1.1}$$

where $\lambda(z)$ is the depth-dependent thermal conductivity, T is temperature, and z is depth below the surface. The computational domain is discretized into uniform grid cells with a specified vertical spacing. Boundary conditions are a fixed temperature at the surface, and a constant heat flux at the bottom of the domain. The thermal conductivity profile $\lambda(z)$ is defined as a piecewise linear function with depth (z), segmented into multiple depth intervals, each characterized by distinct conductivity values representing the main geological layers. Within each interval, conductivity varies linearly (interpolated) according to

$$\lambda(z) = \lambda i + (\lambda_{i+1} - \lambda_i) \cdot \frac{(z - z_i)}{(z_{i+1} - z_i)} \tag{1.2}$$

where λ_i and λ_{i+1} are the conductivity values at depths z_i and z_{i+1}, respectively. The resulting 1D temperature profile is then extended laterally and interpolated onto the 3D inversion mesh, yielding a spatial temperature field $T(\vec{x})$ defined at each cell of the model.

2. Source rock volume. The code performs a PGI inversion[1,2] that minimizes an objective function of the form

$$\Phi(\vec{m}) = \Phi_d(\vec{m}) + \beta \cdot \Phi_{petro}(\vec{m}) + \alpha \cdot \Phi_{reg}(\vec{m}) \tag{2.1}$$

where $\Phi_d(\vec{m})$ represents the data misfit between observed and predicted gravity and magnetic data, $\Phi_{petro}(\vec{m})$ is a petrophysical penalty based on a Gaussian Mixture Model (GMM) built from density (ρ) and magnetic susceptibility (χ), $\Phi_{reg}(\vec{m})$ is a regularization term promoting model smoothness or closeness to prior information, β controls the influence of the petrophysical term in the inversion, and α regulates the model regularization. This inversion produces a model $\vec{m}$ that includes the density $\rho(\vec{x})$ and magnetic susceptibility $\chi(\vec{x})$ values for all mesh voxels (smallest cube of space with defined properties). After inversion, for each voxel at spatial position $\vec{x}$, the recovered values of density $\rho(\vec{x})$ and magnetic susceptibility $\chi(\vec{x})$ are evaluated. Voxels whose properties fall within the predefined range (or cluster) associated with serpentinite are flagged using a binary mask of the form

$$M_{serp}(\vec{x}) = \begin{cases} 1, & if\ \rho(\vec{x})\ and\ \chi(\vec{x})\ are\ consistent\ with\ serpentinite \\ 0, & otherwise \end{cases} \tag{2.2}$$

Each identified serpentinite voxel is assigned a volume $V_{voxel} = \Delta x \cdot \Delta y \cdot \Delta z$, corresponding to the discretization dimensions of the mesh. Summing the volumes of all voxels where $M_{serp}(\vec{x}) = 1$ yields the total volume of serpentinite. After that, the selected volumes are filtered by temperature using a mask that defines the temperature range considered for hydrogen generation

$$M_T(\vec{x}) = \begin{cases} 1, & \text{if } 100°\text{C} \leq \text{T}(\vec{x}) \leq 500°\text{C} \\ 0, & otherwise \end{cases} \tag{2.3}$$

The final filtered serpentinite volume corresponds to voxels where both $M_{serp}(\vec{x}) = 1$ and $M_T(\vec{x}) = 1$. Subsequently, this temperature-filtered serpentinite volume is further subdivided into discrete temperature intervals T (e.g., 100-125°C, 125-150°C, etc.). For each interval, the

corresponding volume of serpentinite is denoted as $V_{serp}(T)$. Each original model voxel is internally subdivided into smaller cells, on which all future calculations are performed, determined by the fracture spacing parameters *($dist_x$, $dist_y$, $dist_z$)*. Thus, one reactive volume before serpentinization correction is

$$V_{cell}^{init} = dist_x \cdot dist_y \cdot dist_z \tag{2.4}$$

3. Serpentinization correction. The correction factor C_{serp} accounts for the reduction in reactive volume caused by prior serpentinization. In our model, we assume that the serpentinization front progressively transforms fresh peridotite into serpentinite as it advances, fully consuming the reactive components in each transformed volume (e.g. Refs.[3–5]), although even fully altered rock may retain a small unreacted fraction of about 2%[6,7]. The relationship between volumetric serpentinization degree and petrophysical properties is derived from reference datasets[8–12], for unaltered peridotite ($\rho_{initial}$= 3.24 g/cm^3, $\chi_{initial}$= 0.0011 SI) and for fully serpentinized rock (ρ_{serp}= 2.62 g/cm^3, χ_{serp}= 0.07 SI). For any given degree of serpentinization, the expected density and susceptibility are computed via weighted averages

$$\rho_{avg} = \rho_{serp} \cdot f + \rho_{initial} \cdot (1 - f) \tag{3.1}$$

$$\chi_{avg} = \chi_{serp} \cdot f + \chi_{initial} \cdot (1 - f) \tag{3.2}$$

where f is the serpentinization degree expressed as a fraction. These expected values are compared with the mode of the inversion-derived density and susceptibility distributions (computed using kernel density estimation) to infer the average degree of serpentinization. To apply a correction due to the partial serpentinization of the reactive volume, we use the following expression

$$C_{serp}(f) = 0.98 \cdot \left[1 - (1 - f)^{\frac{2}{3}}\right] \tag{3.3}$$

This equation reflects the fraction of rock volume that has been geometrically consumed by an isotropic serpentinization front, assuming uniform advance in all directions. The correction is applied to the volume of each cell giving the reactive volume V_{cell} as

$$V_{cell} = V_{cell}^{init} \cdot C_{serp}(f) \tag{3.4}$$

where V_{cell}^{init} is the total serpentinite volume within each cell (from eq. 2.4) and $C_{serp}(f)$ scales the result based on the degree of prior alteration.

4. Serpentinization front velocity. The propagation velocity of the serpentinization front, $v_f(T,P)$, is estimated by scaling a reference velocity based on the relative hydrogen generation rates across temperature intervals (i.e., the front advances more rapidly in regions where the generation of hydrogen is higher). A reference temperature-pressure bin, typically associated with experimentally constrained conditions, is selected along with its corresponding serpentinization front velocity v_{ref}. For each temperature-pressure interval, the front velocity is calculated as

$$v_f(T,P) = v_{ref}(T_{ref}, P_{ref}) \cdot \left[\frac{R_{H2}(T,P)}{R_{H2}^{ref}(T_{ref},P_{ref})}\right] \tag{4.1}$$

where v_{ref} is a reference velocity, R_{H2} is the hydrogen generation rate, and R_{H2}^{ref} is the hydrogen generation rate under reference conditions.

5. Pressure gradients. Pressure was estimated using a depth-dependent gradient transitioning from hydrostatic to lithostatic conditions. For shallow depths (≤ 3,000 m), pressure is purely hydrostatic. Between 3,000 m and 20,000 m, we applied piecewise linear interpolations that progressively increase the pressure from slightly above hydrostatic (1.05×) to a fraction of the lithostatic gradient (up to 0.75×). For depths > 20,000 m, pressure follows a reduced lithostatic gradient

$$P(z) = \begin{cases} \rho_{water} \cdot g \cdot z, & z \leq 3000\ m \\ Linear\ transition, & 3000 < z \leq 20000\ m \\ 0.75 \cdot \rho_{rock} \cdot g \cdot z, & z > 20000\ m \end{cases} \tag{5.1}$$

6. Deep water flow capacity. Flow through the fractured medium is computed using Darcy's law, where the volumetric flow rate Q is defined as

$$Q = \frac{k \cdot A_f \cdot \Delta P}{\mu \cdot L} \tag{6.1}$$

where k is the permeability, A_f is the connected fracture surface area, ΔP is the pressure differential driving the flow, μ is the dynamic viscosity of the water, and L is the characteristic flow path

length. Permeability k follows an empirical trend based on depth-dependent fracture scaling[13], expressed as distributions reflecting natural variability in subsurface properties

$$log_{10}k = -3.2\ log_{10}(z) - 14 + \ \varepsilon \tag{6.2}$$

where z is the depth and ε is a random perturbation drawn from a uniform distribution, representing natural scatter in permeability observations. The fracture surface area A_f for each sample is computed from the number of through-going fractures across the damage zone

$$A_f = n_f \cdot L_{frac} \cdot z \tag{6.3}$$

where n_f is the number of fractures (itself dependent on fracture density and damage zone width), L_{frac} is the effective fractured length of the fault (a fraction of the total fault length), and z is the depth. A connection fraction f_c is applied to simulate not-hydraulically connected segments, yielding the effective surface area

$$A_{f_{eff}} = A_f \cdot f_c \tag{6.4}$$

Each simulation computes the expected flow Q, which is then compared against the design target. The simulation outputs the proportion of configurations for which the system can sustain or exceed the target flow.

The target water flow is allocated among temperature intervals using a weighted distribution, in which Q_i is the assigned flow for temperature interval i defined as

$$Q_i = Q_{tot} \cdot \frac{\left(\frac{V_i}{V_{tot}}\right) \cdot D_i \cdot \ P_i}{\Sigma_j\left(\frac{V_j}{V_{tot}}\right) \cdot D_j \cdot P_j} \tag{6.5}$$

where Q_{tot} is the total target water flow, V_i is the volume associated with interval i, V_{tot} is the total volume across all intervals, D_i is the depth penalty factor for interval i, penalizing shallower (cooler) intervals, and P_i is the normalized hydrogen generation factor for interval i, relative to the maximum productive interval.

7. Estimation of H_2 solubility. Hydrogen solubility in pure water is expressed as molality $m\ (T, P)$, defined as the number of moles of dissolved molecular hydrogen per kilogram of water.

Solubility is calculated using the Krichevsky-Kasarnovsky (KK) relation explicitly accounting for gas non-ideality and pressure effects in solution

$$m(T,P) = m_0(T) \cdot \left(\frac{\phi(T,P) \cdot P}{P_0}\right) \cdot \exp\left[-\frac{V_\infty .(P-P_0)}{R \cdot T}\right] \tag{7.1}$$

where T and P are temperature and pressure, $P_0 = 0.1$ Mpa is the reference pressure, R is the universal gas constant, $\phi(T,P)$ is the fugacity coefficient of H_2, and V_∞ is the partial molar volume of dissolved hydrogen at infinite dilution, and $m_0(T)$ is the reference solubility at P_0. Fugacity coefficients were computed using the Peng-Robinson equation of state for pure hydrogen

$$ln\, \phi(T,P) = Z_g - 1 - ln(Z_g - B) - \frac{A}{2\sqrt{2}B} ln\left(\frac{Z_g + (1+\sqrt{2})B}{Z_g + (1-\sqrt{2})B}\right) \tag{7.2}$$

where Z_g is the vapour-phase compressibility factor. The Peng–Robinson dimensionless parameters A and B are defined as

$$A(T,P) = \frac{a(T)P}{R^2T^2} \qquad B(T,P) = \frac{bP}{RT} \tag{7.3}$$

with

$$a(T) = 0.45724\, \frac{R^2T_c^2}{P_c}\, \alpha(T) \qquad b = 0.07780 \frac{RT_c}{P_c} \tag{7.4}$$

The temperature dependence is given by

$$\alpha(T) = \left[1 + \kappa\left(1 - \sqrt{\frac{T}{T_c}}\right)\right]^2, \qquad \kappa = 0.37464 + 1.54226\omega - 0.26992\omega^2 \tag{7.5}$$

with critical properties for hydrogen $T_c = 33.19$ K, $P_c = 1.293 \times 10^6$ Pa, $\omega = -0.216$.

The reference solubility $m_0(T)$ and the partial molar volume at infinite dilution V_∞ were inferred from a compilation of H_2-H_2O experimental measurements covering a broad range of temperatures and pressures[14]. Reported hydrogen mole fractions x_{H2} were converted to molality assuming 1 kg of water (n_{H2O} = 55.51 mol) according to

$$m = 55.51\, \frac{x_{H2}}{1 - x_{H2}} \tag{7.6}$$

Apparent $m_0(T)$ were obtained by rearranging Eq. (7.1), using fugacity coefficients computed with the Peng–Robinson equation of state. The parameter V_∞ was estimated by minimizing the residual pressure dependence of $m_0(T)$ at fixed temperature, thereby removing spurious pressure dependence of the reference solubility. Final $m_0(T)$ values were obtained by aggregating measurements at each temperature, using the median to reduce sensitivity to experimental scatter. The resulting $m_0(T)$ function was interpolated and used in the forward solubility calculations.

8. Hydrogen generation without constrains. All quantities are computed at the individual cell scale and subsequently aggregated over the full reactive volume. For each cell in the model domain, the temporal evolution of hydrogen generation is estimated by computing the rate at which the serpentinization front advances without limitations (i.e., generation is determined solely by the volume of serpentinized rock and its intrinsic capacity to generate H_2). The rate of change of the volume of serpentinized rock V_{front} is given by

$$\frac{dV_{front}(t,T,P)}{dt} = A_{front} \cdot v_f(T,P) \tag{8.1}$$

where A_{front} is effective surface area of the serpentinization front for one cell and v_f is the front propagation velocity. Assuming isotropic geometry for the cells, the fixed reactive surface area is estimated as

$$A_{front} \propto [V_{cell}]^{\frac{2}{3}} \tag{8.2}$$

where V_{cell} is the available reactive volume already reduced according to the degree of prior serpentinization. Assuming full reactivity of the reacting volume, hydrogen generation for one cell n_{H2}^{unc} at time *t* is computed as

$$\frac{dn_{H2}^{unc}(t,T,P)}{dt} = \rho_{rock} \cdot R_{H2}(T,P) \cdot \frac{dV_{front}(t,T,P)}{dt} \tag{8.3}$$

where serpentinized peridotite cells contribute to hydrogen generation at the local thermodynamic yield R_{H2} for the specific scenarios (k_v). To estimate the total hydrogen generation, the individual contributions from all reactive cells within the effective serpentinized volume V_{serp} across all temperature intervals are summed and integrated over time

$$n_{H2}^{total\_unc}(t,T,P) = \sum_T \sum_{cells \in V_{serp}(T)} \int_0^t \frac{dn_{H2}^{unc}(t,T,P)}{dt} dt \quad (8.4)$$

9. Hydrogen generation estimation with constrains. Hydrogen generation is affected by several limiting processes, including fluid saturation, water delivery, and porosity evolution. Time dependence arises exclusively from the progressive advance of the serpentinization front. External water inflow and thermodynamic properties are treated as quasi-steady over the timescale of individual simulation steps. As the serpentinization front progresses, it generates additional porosity that facilitates water infiltration. This newly formed porosity, however, is progressively reduced as secondary minerals precipitate and fill the pore space. The total water input at one cell (M_{H2O}) at each time step reflects both externally supplied inflow and the internal accommodation required to fill newly generated porosity, as no voids are permitted in the solid framework under mass conservation principles.

$$M_{H2O}(t,T,P) = \phi_p \cdot \rho_{H2O}(T,P) \cdot \frac{dV_{front}(t,T,P)}{dt} + J_{in}(T,P) \quad (9.1)$$

where ϕ_p is the porosity produced by the reaction, V_{front} represents the advance of the reactive volume, ρ_{H2O} is the density of the water and J_{in} is the water input rate from diffusion and fracture-controlled flow determined by Eq. (9.6). Diffusive inflow across the outer shell of the cell up to the reactive nucleus J_{diff} is governed by Fick's law and depends on the temperature-dependent effective diffusivity

$$J_{diff}(T,P) = D_{eff}(T) \cdot \frac{A_{ext}}{\delta} \cdot \rho_{H2O}(T,P) \quad (9.2)$$

where D_{eff} is the effective diffusivity, δ is the shell thickness (distance to the unreacted core), A_{ext} the external face area and ρ_{H2O} is the density of water. Fracture-driven inflow is estimated via Darcy's law, assuming flow through internal fractures spaced by s within the reactive shell. The number of fractures in the shell is

$$n_{fract} = \max\left(1, \frac{\delta}{s}\right) \quad (9.3)$$

Each fracture spans the external face A_{ext}, and the total fracture surface area is approximated as

$$A_{\text{fract}} = n_{fract} \cdot A_{ext} \cdot 6 \quad (9.4)$$

The fracture-driven mass inflow is then

$$J_{fract}(T,P) = \frac{k \cdot A_{\mathrm{fract}}}{\mu(T) \cdot \delta} \cdot \Delta P \cdot \rho_{H2O}(T,P) \tag{9.5}$$

where k is the fracture permeability, μ is the temperature-dependent dynamic viscosity of water, ΔP is the pressure differential across the shell and δ is the shell thickness (distance to the unreacted core). The shell refers to the partially reacted volume surrounding the unreacted core within each discretized cell. The total external inflow is the sum of diffusive and fracture contributions

$$J_{in}(T,P) = J_{diff}(T,P) + J_{fract}(T,P) \tag{9.6}$$

The ability of the water to retain hydrogen is limited by solubility under in-situ conditions. The maximum number of moles of hydrogen that can be dissolved in the available water within a cell is

$$n_{H2}^{max}(t,T,P) = m(T,P) \cdot M_{H2O}(t,T,P) \tag{9.7}$$

where M_{H2O} is the mass of water available in the reactive zone. To avoid saturation, the actual hydrogen generation must not exceed this capacity. Hydrogen saturation is defined as the ratio between the amount of dissolved hydrogen and the maximum amount that can be retained by the available water,

$$S_{H2}(t,T,P) = \frac{n_{H2}^{unc}(\mathrm{t,T,P})}{n_{H2}^{max}(t,T,P)} \tag{9.8}$$

The saturation level of hydrogen ($S_{H2}(t + \Delta t)$) in the infiltrating water is updated at each time step to account for the accumulation of dissolved H_2

$$S_{H2}(t + \Delta t, T, P) = \min\left(1.0,\ S_{H2}(t,T,P) + \frac{n_{H2}^{unc}(\mathrm{t,T,P})}{n_{H2}^{max}(t,T,P)}\right) \tag{9.9}$$

where $S_{H2}(t)$ is the hydrogen saturation from the previous step, M_{H2O} is the mass of water available in the reactive zone at time t. Once saturation approaches unity, the chemical potential for further dissolution vanishes, halting further generation unless water is renewed. The actual hydrogen generation for a cell n_{H2}^{cell} is computed by scaling the unconstrained yield by the available capacity of the aqueous phase to retain additional H_2

$$\frac{dn_{H2}^{cell}(\mathrm{t,T,P})}{dt} = n_{H2}^{unc}(t,T,P) \cdot [1 - S_{H2}(t,T,P)] \tag{9.10}$$

To obtain the total hydrogen generation under constrained conditions n_{H2}^{total}, the generation rate for each reactive cell must be computed individually and then aggregated over the full serpentinized volume. This is done by summing the constrained generation over all contributing cells in V_{serp} for each temperature-pressure range (T, P) and integrating over time

$$n_{H2}^{total}(T,P) = \sum_{T,P} \sum_{cells \in V_{serp}(T,P)} \int_0^t \frac{dn_{H2}^{cell}(t,T,P)}{dt} dt \tag{9.11}$$

10. Uncertainty estimation (Monte Carlo framework). Uncertainties in hydrogen generation are quantified using a Monte Carlo (MC) framework in which uncertainties in physical, geometrical, hydraulic, and geochemical parameters are propagated through the model. For each Monte Carlo realization j, a vector of uncertain parameters

$$\theta^{(j)} = \left\{\theta_1^{(j)}, \theta_2^{(j)}, \dots, \theta_n^{(j)}\right\} \tag{10.1}$$

is sampled within physically plausible bounds and used to evaluate hydrogen generation independently for each temperature interval T. For each temperature interval T and realization j, the amount of hydrogen produced is computed as

$$n_{H2}^{(j)}(T,P) = \min\left[\left(\prod_{k=1}^{n} f_k^{(j)}\right) \cdot n_{H2}^{ref}(T,P)\,,\, n_{H2}^{sat,(j)}(T,P)\right] \tag{10.2}$$

where the first term represents kinetically controlled hydrogen generation in the temperature interval T, scaled by multiplicative uncertainty factors accounting for serpentinization front velocity, volumetric generation rate, effective reactive volume, and serpentinization efficiency. The second term represents the effective upper bound imposed by water availability and hydrogen solubility at the pressure and temperature conditions associated with interval T. This formulation naturally reduces to the unconstrained (theoretical) case when saturation limits are not active. Parameter sampling is performed using quasi-random low-discrepancy Sobol sequences to improve convergence of ensemble statistics relative to purely random sampling. A fixed global random seed is used to ensure reproducibility of all Monte Carlo ensembles.

The total amount of hydrogen generated in realization j is obtained by summation over all temperature intervals

$$n_{H2}^{total,(j)}(T,P) = \sum_i n_{H2}^{(j)}(T,P) \quad (10.3)$$

Uncertainty bounds reported in this study correspond to the ensemble mean and standard deviation of n_{H2}^{total} and its temperature-resolved contributions.

11. Univariate sensitivity analysis. A univariate sensitivity analysis is performed to identify the first-order controls on hydrogen generation and to support the interpretation of the Monte Carlo results. In this analysis, hydrogen generation is evaluated by varying one model parameter at a time while all others are fixed at their reference values. For a given temperature-pressure interval (T, P), hydrogen generation is expressed as

$$n_{H2}(T,P;\ \tau) = \min\left[\tau \cdot n_{H2}^{ref}(T,P), n_{H2}^{sat}(T,P)\right] \quad (11.1)$$

where τ is a dimensionless scaling factor applied to the selected parameter (e.g., serpentinization front velocity, effective reactive volume, fracture-controlled water delivery, or hydrogen solubility), while all other parameters are kept at their nominal values. The reference term $n_{H2}^{ref}(T,P)$ corresponds to hydrogen generation evaluated using the baseline model configuration. The saturation term explicitly accounts for the thermodynamic dependence of hydrogen solubility on temperature and pressure. The analysis is performed independently for each (T, P) interval and aggregated to obtain the total hydrogen generation,

$$n_{H2,tot}(\tau) = \sum_{T,P} n_{H2}(T,P;\ \tau)$$

sensitivity is quantified by the variation of $n_{H2,tot}(\tau)$ relative to its reference

$$n_{H2,tot}^{ref} \equiv n_{H2,tot}(\tau = 1)$$

allowing identification of parameters exerting dominant versus secondary control on hydrogen generation across the modeled pressure-temperature range.

Parameters used in the calculations

Supplementary Table 1: Parameters used in the calculations in the Western Pyrenees

Parameter	Value/Range	Reference/Comments
**Initial Model**		
Seismic Velocity Model	Vp ≈ 5.0 to 8.0 km s^{-1}	[15]
Geological Units	3 Units: Serpentinites, Mantle and Crust	[12,16,17]
**Temperature Calculations**		
Surface Temperature	10 °C	[18]
Background Heat Flow	0.070 Wm^{-2}	[19]
Thermal Conductivity and Depth Intervals	1.1-4.0 W m^{-1} K^{-1} across multiple depth intervals	[20,21]
Number of Simulations	50	-
Temperature Mesh Size (1D)	50 m	-
**Mesh data**		
Mesh Expansion	10% (rectangular)	To avoid edge effects
Mesh Cell Dimensions	750 m × 750 m × 500 m	Maximum voxel size
Model Depth	Up to 20000 m	Maximum depth
**Petrophysical data and inversion constrains**		
Crust	Dens: 2.60 ± 0.07 g cm^{-3}; Mag Sus: 0.001 ± 0.003 SI	[12,16,17]
Serpentinite	Dens: 2.85 ± 0.04 g cm^{-3}; Mag Sus:0.030 ± 0.005 SI	[12,16,17]
Mantle	Dens: 3.05 ± 0.04 g cm^{-3}; Mag Sus:0.0001 ± 0.003 SI	[12,16,17]
Inversion Parameter Bounds	Dens: 2.2-3.5 g cm^{-3}; Mag Sus: 0-0.1 SI	Limits consistent with the petrophysical properties of the units present in the area
**Source rocks**		
Fracture Spacing	1.00 m in x, y, and z	[5,22,23]
Porosity at reaction front	8%	[24]
Fracture spacing in serpentinites	0.05 m	Present study
Permeability of internal fractures	1.0E-20 m^2	[25]
Rock Type	Lherzolite	[26]
Water-to-rock ratio	0.16	Present study
**External water flow simulation**		
Design Flow Target	5.0E+05 L day^{-1}	Present study
Fault Length	50 km	Present study
Damage Zone Thickness	1 - 1000 m	[27]
Fractured Length Fraction	5% - 30%	[28,29]
Depth Range	1000 - 12000 m	Present study
Fracture Density	~1/100 to 1/5 fractures per meter, depth-dependent	Present study
Pore Pressure Excess (ΔP)	60 - 100 MPa	Present study
Dynamic Viscosity (μ)	1.0E-03 – 1.0E-05 Pa s	Present study

Connected Fraction of Fractures	10% - 25%	[28,29]
Permeability	$\log_{10} k = -3.2 \cdot \log_{10}(z/1000) - 14 \pm 0.3$	[13]
**Other parameters**		
Reference front velocity	1.27E-05 cm day^{-1}	[30]
Reference temperature	300-325°C	[30]
Standard deviation	7.68E-06 cm day^{-1}	[30]

Supplementary Table 2: Parameters used in the calculations in Northern California

Parameter	Value/Range	Reference/Comments
**Initial Model**		
Seismic Velocity Model	Vp ≈ 4.6 to 7.4 km s^{-1}	[31]
Geological Units	3 Units: Crust, Serpentinites and Sediments	[32–34]
**Temperature Calculations**		
Surface Temperature	15 °C	[35]
Background Heat Flow	0.095 W m^{-2}	[19]
Thermal Conductivity and Depth Intervals	1.1-4.0 W m^{-1} K^{-1} across multiple depth intervals	[20,21]
Number of Simulations	50	Present study
Temperature Mesh Size (1D)	100 m	Present study
**Mesh data**		
Mesh Expansion	10% (Square)	To avoid edge effects
Mesh Cell Dimensions	1000 m × 1000 m × 500 m	Maximum voxel size
Model Depth	Up to 20000 m	Maximum depth
**Petrophysical data and inversion constrains**		
Crust	Dens: 2.80 ± 0.05 g cm^{-3}; Mag Sus: 0.001 ± 0.005 SI	[32,34]
Serpentinite	Dens: 2.63 ± 0.07 g cm^{-3}; Mag Sus: 0.07 ± 0.007 SI	[32,34]
Sediments	Dens: 2.40 ± 0.05 g cm^{-3}; Mag Sus: 0.001 ± 0.005 SI	[32,34]
Inversion Parameter Bounds	Dens: 1.7-3.5 g cm^{-3}; 0- Mag Sus: 0.15 SI	Limits consistent with the petrophysical properties of the units present in the area
**Source rocks**		
Fracture Spacing	1.00 m in x, y, and z	[5,22,23]
Porosity at reaction front	8%	[24]
Fracture spacing in serpentinites	0.05 m	Present study
Permeability of internal fractures	1.0E-20 m^2	[25]
Rock Type	Harzburgite	[36]
Water-to-rock ratio	0.20	Present study
**External water flow simulation**		
Design Flow Target	2.0E+06 L day^{-1}	Present study
Fault Length	250 km	Present study
Damage Zone Thickness	1 - 1000 m	[27]

Fractured Length Fraction	5% - 30%	[28,29]
Depth Range	1000 - 12000 m	Present study
Fracture Density	~1/100 to 1/5 fractures per meter, depth-dependent	Present study
Pore Pressure Excess (ΔP)	60 - 100 MPa	Present study
Dynamic Viscosity (μ)	1.0E-03 – 1.0E-05 Pa s	Present study
Connected Fraction of Fractures	10% - 25%	[28,29]
Permeability	$\log_{10} k = -3.2 \cdot \log_{10}(z/1000) - 14 \pm 0.3$	[13]
**Other parameters**		
Reference front velocity	1.27E-05 cm day^{-1}	[30]
Reference temperature	300-325°C	[30]
Standard deviation	7.68E-06 cm day^{-1}	[30]

Supplementary Table 3: H_2 generation for 1 year under unconstrained conditions for the Western Pyrenees

Temp [°C]	Pressure [MPa]	Solubility [mol kg^{-1}]	H2O front [kg day^{-1}]	H2O abs [kg day^{-1}]	Annual H_2 [tonnes yr^{-1}]	Std clip [tonnes yr^{-1}]	Sat ratio [-]
100_125	45.73	3.38E-01	-	-	-	-	-
125_150	56.55	4.17E-01	-	-	-	-	-
150_175	67.87	5.00E-01	-	-	-	-	-
175_200	80.25	5.90E-01	1.85E+05	9.12E+04	1004.20	549.77	24.55
200_225	97.27	7.13E-01	2.25E+06	1.12E+06	12660.44	6931.22	21.39
225_250	115.97	8.47E-01	2.94E+05	1.48E+05	1795.57	983.02	19.78
250_275	136.69	9.97E-01	1.21E+07	6.13E+06	74281.12	40666.74	17.09
275_300	158.94	1.16E+00	1.65E+07	8.50E+06	106435.19	58270.16	15.54
300_325	183.07	1.33E+00	-	-	-	-	-
325_350	209.23	1.52E+00	-	-	-	-	-
350_375	242.29	1.76E+00	-	-	-	-	-
375_400	283.0	2.04E+00	-	-	-	-	-
400_425	326.55	2.35E+00	-	-	-	-	-
425_450	375.68	2.70E+00	-	-	-	-	-
450_475	424.83	3.05E+00	-	-	-	-	-
475_500	437.8	3.15E+00	-	-	-	-	-
TOTAL	-	-	3.133E+07	1.599E+07	196,177	107,401	-

Descriptions: Temp, temperature interval; Pressure, average pressure; Solubility, estimated H_2 solubility; H2O front, water at the reaction front; H2O abs, water absorbed into the solid phase; Annual H_2, annual H_2 generation; Std clip, winsorized standard deviation of Annual H_2 (multiplicative uncertainty clipped to the 1st–99th percentiles); Sat ratio, ratio of H_2 generation to H_2 dissolution capacity. Note: we assume 1L of water equivalent to 1kg.

Supplementary Table 4: H_2 generation for 1 year under unconstrained conditions for Northern California

Temp [°C]	Pressure [MPa]	Solubility [mol kg^{-1}]	H2O front [kg day^{-1}]	H2O abs [kg day^{-1}]	Annual H_2 [tonnes yr^{-1}]	Std clip [tonnes yr^{-1}]	Sat ratio [-]
100_125	29.67	2.24E-01	1.46E+06	7.82E+05	8551	4681	75.96
125_150	36.19	2.73E-01	2.98E+06	1.60E+06	19617	10739	70.86
150_175	43.13	3.25E-01	3.46E+06	1.87E+06	27424	15014	72.11

175_200	49.94	3.76E-01	4.62E+06	2.54E+06	38169	20897	66.22
200_225	57.81	4.35E-01	7.64E+06	4.28E+06	65803	36025	61.07
225_250	66.03	4.96E-01	6.82E+06	3.89E+06	61231	33522	57.23
250_275	73.51	5.53E-01	7.86E+06	4.61E+06	75278	41213	57.01
275_300	83.02	6.24E-01	7.05E+06	4.24E+06	70245	38457	54.39
300_325	94.45	7.09E-01	2.94E+06	1.61E+06	12109	6629	17.38
325_350	107.10	8.04E-01	-	-	-	-	-
350_375	119.83	8.99E-01	-	-	-	-	-
375_400	134.64	1.01E+00	-	-	-	-	-
400_425	149.84	1.12E+00	-	-	-	-	-
425_450	166.04	1.24E+00	-	-	-	-	-
450_475	182.98	1.37E+00	-	-	-	-	-
475_500	200.44	1.50E+00	-	-	-	-	-
TOTAL	-	-	4.48E+07	2.56E+07	324,426	177,177	-

Descriptions: Temp, temperature interval; Pressure, average pressure; Solubility, estimated H_2 solubility; H2O front, water at the reaction front; H2O abs, water absorbed into the solid phase; Annual H_2, annual H_2 generation; Std clip, winsorized standard deviation of Annual H_2 (multiplicative uncertainty clipped to the 1st–99th percentiles); Sat ratio, ratio of H_2 generation to H_2 dissolution capacity. Note: we assume 1L of water equivalent to 1kg.

Supplementary Table 5: H_2 generation for 1 year under constrained conditions for the Western Pyrenees for a flow rate of 5×10^5 L day^{-1}.

Temp [°C]	Flow [kg d^{-1}]	Diff [kg d^{-1}]	Frac [kg d^{-1}]	H_2O abs [kg d^{-1}]	Sol [mol kg^{-1}]	Sat [mol kg^{-1}]	Eff [%]	H2 Tot [tonnes yr^{-1}]	Std [tonnes yr^{-1}]
100_125	-	-	-	-	-	-	-	-	-
125_150	-	-	-	-	-	-	-	-	-
150_175	-	-	-	-	-	-	-	-	-
175_200	3.93E+03	2.89E-06	4.47E+03	1.27E+02	0.589	0.480	0.20	1.39	0.44
200_225	4.42E+04	5.36E-05	5.03E+04	1.68E+03	0.712	0.583	0.22	18.95	6.04
225_250	5.36E+03	1.01E-05	6.10E+03	2.28E+02	0.846	0.697	0.23	2.75	0.87
250_275	2.00E+05	5.45E-04	2.27E+05	1.01E+04	0.996	0.825	0.24	121.24	38.50
275_300	2.47E+05	9.37E-04	2.81E+05	1.41E+04	1.156	0.965	0.25	175.34	55.58
300_325	-	-	-	-	-	-	-	-	-
325_350	-	-	-	-	-	-	-	-	-
350_375	-	-	-	-	-	-	-	-	-
375_400	-	-	-	-	-	-	-	-	-
400_425	-	-	-	-	-	-	-	-	-
425_450	-	-	-	-	-	-	-	-	-
450_475	-	-	-	-	-	-	-	-	-
475_500	-	-	-	-	-	-	-	-	-
TOTAL	4.97E+05	1.55E-03	5.69E+05	2.61E+04	-	-	-	319.7	101.4

Descriptions: Temp, temperature interval; Flow, maximum clean-water delivery per day; Diff, diffusive clean-water inflow; Frac, fracture-driven clean-water inflow; H2O abs, clean water absorbed by the rock; Sol, hydrogen solubility in water; Sat, dissolved H_2 per kilogram of delivered water; Eff, efficiency relative to the no-saturation case; H2 Tot, total hydrogen produced; Std, standard deviation of total hydrogen generation. Note: we assume 1L of water equivalent to 1kg.

Supplementary Table 6: H_2 generation for 1 year under constrained conditions for Northern California for a flow rate of 2×10^6 L day^{-1}.

Temp [°C]	Flow [kg d^{-1}]	Diff [kg d^{-1}]	Frac [kg d^{-1}]	H2O abs [kg d^{-1}]	Sol [mol kg^{-1}]	Sat [mol kg^{-1}]	Eff [%]	H2 Tot [tonnes yr^{-1}]	Std [tonnes yr^{-1}]
100_125	8.64E+04	3.64E-06	9.83E+04	964	0.224	0.164	0.17	10.5	3.46
125_150	1.67E+05	1.61E-05	1.89E+05	2.03E+03	0.273	0.201	0.17	24.7	8.15
150_175	1.84E+05	3.71E-05	2.09E+05	2.24E+03	0.325	0.241	0.16	32.6	10.8
175_200	2.31E+05	9.16E-05	2.63E+05	3.2E+03	0.376	0.281	0.17	47.8	15.7
200_225	3.58E+05	2.55E-04	4.07E+05	5.65E+03	0.434	0.328	0.18	86.3	28.3
225_250	2.97E+05	3.59E-04	3.38E+05	5.28E+03	0.496	0.377	0.19	82.5	27
250_275	3.18E+05	6.32E-04	3.62E+05	6.12E+03	0.552	0.423	0.18	99.1	32.4
275_300	2.60E+05	7.86E-04	2.96E+05	5.62E+03	0.624	0.483	0.19	92.5	30.1
300_325	9.77E+04	4.20E-04	1.11E+05	5.33E+03	0.709	0.554	0.47	39.9	12.9
325_350	-	-	-	-	-	-	-	-	-
350_375	-	-	-	-	-	-	-	-	-
375_400	-	-	-	-	-	-	-	-	-
400_425	-	-	-	-	-	-	-	-	-
425_450	-	-	-	-	-	-	-	-	-
450_475	-	-	-	-	-	-	-	-	-
475_500	-	-	-	-	-	-	-	-	-
TOTAL	1.99E+06	2.60E-03	2.27E+06	3.64E+04	-	-	-	516.9	168.8

Descriptions: Temp, temperature interval; Flow, maximum clean-water delivery per day; Diff, diffusive clean-water inflow; Frac, fracture-driven clean-water inflow; H2O abs, clean water absorbed by the rock; Sol, hydrogen solubility in water; Sat, dissolved H_2 per kilogram of delivered water; Eff, efficiency relative to the no-saturation case; H2 Tot, total hydrogen produced; Std, standard deviation of total hydrogen generation. Note: we assume 1L of water equivalent to 1kg. Note: we assume 1L of water equivalent to 1kg.

Supplementary Table 7: H_2 generation for 1 year under constrained conditions according to imposed water flow rates for the Western Pyrenees**.**

Flow [L day^{-1}]	H_2 total [tonnes]	Std [tonnes]	Conc [mol kg^{-1}]	SatTime [days]	Turnover [days]	Turnover [% day^{-1}]	Limit
1.00E+01	0.01	0.00	0.8793	3.16E+06	3.55E+06	0.00	water
2.04E+01	0.02	0.00	0.8792	1.55E+06	1.74E+06	0.00	water
4.18E+01	0.03	0.01	0.8792	7.57E+05	8.51E+05	0.00	water
8.53E+01	0.06	0.02	0.8792	3.71E+05	4.17E+05	0.00	water
1.74E+02	0.13	0.04	0.8790	1.81E+05	2.04E+05	0.00	water
3.56E+02	0.26	0.08	0.8785	8.88E+04	9.98E+04	0.00	water
7.28E+02	0.53	0.17	0.8772	4.36E+04	4.88E+04	0.00	water
1.49E+03	1.09	0.34	0.8751	2.14E+04	2.39E+04	0.00	water
3.04E+03	2.22	0.69	0.8721	1.05E+04	1.17E+04	1.00E-02	water
6.21E+03	4.51	1.39	0.8677	5.17E+03	5.72E+03	2.00E-02	water
1.27E+04	9.15	2.83	0.8615	2.55E+03	2.80E+03	4.00E-02	water
2.59E+04	18.50	5.73	0.8526	1.26E+03	1.37E+03	7.00E-02	sat
5.30E+04	37.25	11.57	0.8403	6.27E+02	6.71E+02	1.50E-01	sat
1.08E+05	74.59	23.24	0.8235	3.14E+02	3.28E+02	3.00E-01	sat
2.21E+05	148.19	46.44	0.8007	1.59E+02	1.61E+02	6.20E-01	sat
4.52E+05	290.98	92.15	0.7694	8.18E+01	7.86E+01	1.27E+00	rate
9.24E+05	562.04	181.14	0.7273	4.32E+01	3.85E+01	2.60E+00	rate

1.89E+06	1061.23	351.36	0.6721	2.38E+01	1.88E+01	5.31E+00	rate
3.86E+06	1939.52	671.85	0.6011	1.40E+01	9.22E+00	1.09E+01	rate
7.88E+06	3401.58	1256.69	0.5159	9.01E+00	4.51E+00	2.22E+01	rate
1.61E+07	5887.83	2443.06	0.4370	6.41E+00	2.21E+00	4.53E+01	rate
3.29E+07	8417.76	4546.31	0.3058	6.29E+00	1.08E+00	9.26E+01	rate
6.72E+07	5191.40	3382.84	0.0923	1.14E+01	5.30E-01	1.89E+02	rate
1.37E+08	2618.68	1699.18	0.0228	2.24E+01	2.60E-01	3.87E+02	rate
2.81E+08	1309.87	850.46	0.0056	4.49E+01	1.30E-01	7.90E+02	rate
5.74E+08	640.44	414.12	0.0013	9.15E+01	6.00E-02	1.61E+03	rate
1.17E+09	318.22	206.54	0.0003	1.85E+02	3.00E-02	3.30E+03	rate
2.40E+09	155.81	101.06	0.0001	3.77E+02	1.00E-02	6.74E+03	rate
4.89E+09	76.27	49.45	0.0000	7.70E+02	1.00E-02	1.38E+04	rate
1.00E+10	37.38	24.24	0.0000	1.57E+03	0.00E+00	2.81E+04	rate

Descriptions: Flow, water-delivery target evaluated (logarithmic sweep); H_2 total, mean total dissolved H_2 produced under solubility constraints; Std, standard deviation of H_2 total ; Conc, mean dissolved H_2 concentration per unit mass of delivered water; SatTime, time required for pore water to reach 99% of the mean H_2 solubility; Turnover days, bulk pore-volume turnover time; Turnover, fraction of pore water replaced per day; Limit, dominant limiting regime controlling H_2 generation (water, saturation, or reaction rate). Note: we assume 1L of water equivalent to 1kg.

Supplementary Table 8: H_2 generation for 1 year under constrained conditions according to imposed water flow rates for Northern California**.**

Flow [L day^{-1}]	H_2 total [tonnes]	Std [tonnes]	Conc [mol kg^{-1}]	Sat time [days]	Turnover [days]	Turnover [% day^{-1}]	Limit
1.00E+01	0.00	0.00	0.3901	5.15E+06	5.16E+06	0.00E+00	water
2.04E+01	0.01	0.00	0.3901	2.52E+06	2.52E+06	0.00E+00	water
4.18E+01	0.01	0.00	0.3901	1.23E+06	1.24E+06	0.00E+00	water
8.53E+01	0.03	0.01	0.3901	6.03E+05	6.05E+05	0.00E+00	water
1.74E+02	0.06	0.02	0.3900	2.95E+05	2.96E+05	0.00E+00	water
3.56E+02	0.12	0.04	0.3899	1.45E+05	1.45E+05	0.00E+00	water
7.28E+02	0.24	0.07	0.3894	7.08E+04	7.09E+04	0.00E+00	water
1.49E+03	0.48	0.15	0.3886	3.47E+04	3.47E+04	0.00E+00	water
3.04E+03	0.99	0.30	0.3874	1.71E+04	1.70E+04	1.00E-02	water
6.21E+03	2.00	0.61	0.3857	8.38E+03	8.31E+03	1.00E-02	water
1.27E+04	4.07	1.24	0.3834	4.13E+03	4.07E+03	2.00E-02	water
2.59E+04	8.24	2.50	0.3800	2.04E+03	1.99E+03	5.00E-02	water
5.30E+04	16.64	5.06	0.3753	1.01E+03	9.74E+02	1.00E-01	rate
1.08E+05	33.41	10.19	0.3689	5.04E+02	4.77E+02	2.10E-01	rate
2.21E+05	66.66	20.44	0.3602	2.53E+02	2.33E+02	4.30E-01	rate
4.52E+05	131.68	40.73	0.3482	1.29E+02	1.14E+02	8.80E-01	rate
9.24E+05	256.53	80.49	0.3320	6.67E+01	5.59E+01	1.79E+00	rate
1.89E+06	490.17	157.48	0.3104	3.56E+01	2.73E+01	3.66E+00	rate
3.86E+06	911.47	304.28	0.2825	1.99E+01	1.34E+01	7.48E+00	rate
7.88E+06	1634.56	575.74	0.2479	1.20E+01	6.55E+00	1.53E+01	rate
1.61E+07	2858.71	1094.38	0.2122	7.87E+00	3.20E+00	3.12E+01	rate
3.29E+07	5058.42	2300.29	0.1838	5.79E+00	1.57E+00	6.38E+01	rate
6.72E+07	4571.53	2950.46	0.0813	8.32E+00	7.70E-01	1.30E+02	rate
1.37E+08	2317.34	1497.66	0.0202	1.63E+01	3.80E-01	2.66E+02	rate
2.81E+08	1227.10	804.54	0.0052	3.12E+01	1.80E-01	5.44E+02	rate
5.74E+08	595.86	388.10	0.0012	6.38E+01	9.00E-02	1.11E+03	rate
1.17E+09	296.46	193.60	0.0003	1.29E+02	4.00E-02	2.27E+03	rate

2.40E+09	146.27	95.65	0.0001	2.61E+02	2.00E-02	4.64E+03	rate
4.89E+09	71.65	46.84	0.0000	5.33E+02	1.00E-02	9.49E+03	rate
1.00E+10	35.09	22.94	0.0000	1.09E+03	1.00E-02	1.94E+04	rate

Descriptions: Flow, water-delivery target evaluated (logarithmic sweep); H_2 total, mean total dissolved H_2 produced under solubility constraints; Std, standard deviation of H_2 total ; Conc, mean dissolved H_2 concentration per unit mass of delivered water; SatTime, time required for pore water to reach 99% of the mean H_2 solubility; Turnover days, bulk pore-volume turnover time; Turnover, fraction of pore water replaced per day; Limit, dominant limiting regime controlling H_2 generation (water, saturation, or reaction rate). Note: we assume 1L of water equivalent to 1kg.

Supplementary Table 9: Univariate sensitivity analysis for unconstrained hydrogen generation

Parameter	Range	Mean [tonnes]	Mean min [tonnes]	Mean max [tonnes]	Δmean [tonnes]	Δ% base	Elasticity
Rock vol	(0.4, 1.6)	196176.513	78470.605	313882.422	235411.816	120.00	1.0000
Prod rate	(0.5, 1.5)	196176.513	98088.257	294264.770	196176.513	100.00	1.0000
Surface area	(0.6, 1.4)	196176.513	117705.908	274647.119	156941.211	80.00	1.0000
Serp cor	(0.8, 1.2)	196176.513	156941.211	235411.816	78470.605	40.00	1.0000
Vol	(0.8, 1.2)	196176.513	156941.211	235411.816	78470.605	40.00	1.0000

Descriptions: Parameter: Name of the factor being varied in the one-at-a-time univariate sweep (all other factors fixed at 1.0). Rock vol, serpentinization-front velocity scaling. Prod rate, factor scaling the volumetric H_2 production rate; Surface area, fracture water–rock reactive surface area per unit rock volume; Serp cor, serpentinization correction factor; Vol, factor scaling the reactive rock volume used in the model. Range, multiplicative interval applied to the baseline value; Mean, mean H_2 production when only that factor is varied; Mean min / Mean max, H_2 generation at the lower and upper bound of the range (all other factors fixed at baseline); Δmean, difference between Mean max and Mean min; Δ% base, Δmean normalized by the baseline mean (all factors = 1.0); Elasticity, normalized sensitivity expressed as percent change in capped H_2 per 1% change in the factor.

Supplementary Table 10: Univariate sensitivity analysis for constrained hydrogen generation

Parameter	Range	Mean [tonnes]	Mean min [tonnes]	Mean max [tonnes]	Δmean [tonnes]	Δ% base	Elasticity
Solubility	(0.7, 1.3)	336.785	235.749	437.821	202.072	60.00	1.0000
Mean press	(0.7, 1.3)	336.415	240.204	431.227	191.023	56.72	0.9453
Vol	(0.8, 1.2)	336.784	336.784	336.784	0.000	0.00	0.0000
Serp deg	(0.8, 1.2)	336.784	336.784	336.784	0.000	0.00	0.0000
Spacing	(0.5, 1.5)	336.784	336.784	336.784	0.000	0.00	0.0000
Perm	(0.8, 1.2)	336.784	336.784	336.784	0.000	0.00	0.0000
Prod rate	(0.8, 1.2)	336.784	336.784	336.784	0.000	0.00	0.0000
Dist	(0.5, 1.5)	336.784	336.784	336.784	0.000	0.00	0.0000
Kg rocks	(0.4, 1.6)	336.784	336.784	336.784	0.000	0.00	0.0000

Descriptions: Parameter, name of the factor being varied in the one-at-a-time univariate sweep (all other factors fixed at 1.0); Solubility, factor scaling the computed H_2 solubility in water; Mean press, factor scaling the mean pressure used for pressure-dependent calculations; Vol, factor scaling the reactive rock volume used in the model; Serp deg, factor scaling the volumetric serpentinization degree; Spacing, factor scaling internal fracture spacing in the reactive blocks; Perm, factor scaling internal fracture permeability in the reactive blocks; Prod rate, factor scaling the volumetric H_2 production rate; Dist, factor scaling the size of the reactive blocks; Kg rocks, factor scaling the reactive rock mass available for H_2 production. Range, multiplicative interval applied to the baseline value; Mean, mean H_2 production when only that factor is varied; Mean min / Mean max, H_2 generation at the lower and upper bound of the range (all other factors fixed at baseline); Δmean, difference between Mean max and Mean min; Δ% base, Δmean normalized by the baseline mean (all factors = 1.0); Elasticity, normalized sensitivity expressed as percent change in capped H_2 per 1% change in the factor.

List of Symbols and Parameters

A_f: Fracture surface area [m²].

A_{ext}: External face area of the cell [m²].

A_{fract}: Total fracture surface area within the shell [m²].

A_{front}: Effective surface area of the serpentinization front for one cell [m²].

$C_{serp}(f)$: Serpentinization correction factor accounting for previously altered rock volume [-].

$D_{eff}(T)$: Effective diffusivity, temperature-dependent [m² s⁻¹].

$dist_x, dist_y, dist_z$: Fracture spacing parameters in the x, y, and z directions [m].

f: Degree of serpentinization, fraction from 0 (unaltered) to 1 (fully serpentinized).

f_c: Connection fraction accounting for hydraulically connected fractures [-].

$f(z)$: Weighting factor between hydrostatic and lithostatic pressure depending on depth z [-].

$f_k^{(j)}$: Multiplicative uncertainty factors applied to kinetic and volumetric terms

j: Monte Carlo realization index [-]

$J_{diff}(T,P)$: Diffusive water inflow [kg s⁻¹].

$J_{fract}(T,P)$: Fracture-driven water inflow [kg s⁻¹].

$J_{in}(T,P)$: Total external water inflow into a cell [kg s⁻¹].

k: Permeability of fractured rock [m²].

k_v: Kinetic scenario factor (minimum, average, maximum) [-].

L: Flow path length, approximated by depth [m].

L_{frac}: Effective fractured length of the fault [m].

$m(T,P)$: Hydrogen solubility (molality) under in-situ conditions [mol H_2 kg⁻¹ H_2O].

$m_0(T)$: Reference hydrogen solubility at reference pressure P_0 [mol H_2 kg⁻¹ H_2O].

$M_{H2O}(t,T,P)$: Mass of water available in the reactive zone at time t [kg].

n_f: Number of fractures [-].

n_{fract}: Number of fractures within the reactive shell [-].

$n_{H2}^{cell}(\mathrm{T},\mathrm{P})$: Hydrogen produced by a cell at conditions T [mol].

$n_{H2}^{max}(T, P)$: Maximum amount of hydrogen that can be dissolved in the available water [mol].

$n_{H2}^{unc}(T, P)$: Hydrogen produced in the absence of solubility constraints [mol].

$n_{H2}^{total}(T, P)$: Total hydrogen generation integrated over all cells and time considering physical constraints [mol].

$n_{H2,tot}$: Total hydrogen generation over all (T, P) intervals for a given sensitivity scaling factor s [mol]

$n_{H2,tot}^{ref}$: Reference total hydrogen generation corresponding to the baseline configuration (s=1 [mol]

$P(z)$: Pressure at depth z [MPa].

P_0: Reference pressure (1 bar) [Pa].

$P_{hydro}(z)$: Hydrostatic pressure gradient [MPa].

$P_{litho}(z)$: Lithostatic pressure gradient [MPa].

ϕ_p: $Porosity$ [%]

$\phi(T, P)$: Fugacity coefficient of H_2 [-].

$\theta^{(j)}$: Vector of uncertain parameters in Monte Carlo realization j

Q: Volumetric flow rate of water through fractures [m^3 s^{-1}].

R: Universal gas constant [J mol^{-1} K^{-1}].

$R_{H2}(T, P)$: Hydrogen generation rate derived from thermodynamic equilibrium simulations [mol H_2 kg^{-1} rock].

s: Spacing between internal fractures [m].

$S_{H2}(t, T, P)$: Hydrogen saturation in water at time t, fraction from 0 (unsaturated) to 1 (fully saturated).

T: Temperature [°C].

T_{ref}: Reference temperature for serpentinization velocity [°C].

τ: Dimensionless scaling factor applied to a single model parameter in the univariate sensitivity analysis [−]

V_∞: Partial molar volume of dissolved hydrogen at infinite dilution [m^3 mol^{-1}].

V_{cell}: Reactive volume of a cell after serpentinization correction [m^3].

V_{cell}^{init}: Initial reactive volume per subdivided cell based on fracture spacing [m^3].

$v_f(T, P)$: Serpentinization front velocity dependent on temperature and kinetic scenario [m day^{-1}].

$V_{serp}(T)$: Volume of serpentinite within a specific temperature range [m^3].

V_{voxel}: Volume of a single voxel in the discretized mesh [m^3].

$\vec{x}$: Spatial coordinates in three-dimensional space [m].

z: Depth below the surface [m].

Z_g: Vapour-phase compressibility factor [-].

δ: Thickness of the reactive shell (distance to unreacted core) [m].

$\lambda(z)$: Depth-dependent thermal conductivity $[\mathrm{W/m \cdot K}]$.

μ: Dynamic viscosity of water [Pa·s].

ρ: Density [kg/m^3].

ρ_{rock}: Density of the host rock [kg m^{-3}].

χ: Magnetic susceptibility [SI].

ϕ: Reaction-generated porosity [-].

References

1. Astic, T. & Oldenburg, D. W. A framework for petrophysically and geologically guided geophysical inversion using a dynamic Gaussian mixture model prior. *Geophysical Journal International* **219**, 1989–2012 (2019).
2. Astic, T., Heagy, L. J. & Oldenburg, D. W. Petrophysically and geologically guided multi-physics inversion using a dynamic Gaussian mixture model. *Geophysical Journal International* **224**, 40–68 (2020).
3. Mével, C. Serpentinization of abyssal peridotites at mid-ocean ridges. *Comptes Rendus. Géoscience* **335**, 825–852 (2003).
4. Debret, B., Nicollet, C., Andreani, M., Schwartz, S. & Godard, M. Three steps of serpentinization in an eclogitized oceanic serpentinization front (Lanzo Massif – Western Alps). *Journal Metamorphic Geology* **31**, 165–186 (2013).
5. Uno, M. & Kirby, S. Evidence for multiple stages of serpentinization from the mantle through the crust in the Redwood City Serpentinite mélange along the San Andreas Fault in California. *Lithos* **336–337**, 276–292 (2019).
6. Huang, R., Sun, W., Song, M. & Ding, X. Influence of pH on Molecular Hydrogen (H2) Generation and Reaction Rates during Serpentinization of Peridotite and Olivine. *Minerals* **9**, 661 (2019).
7. Huang, R., Sun, W., Ding, X., Zhao, Y. & Song, M. Effect of pressure on the kinetics of peridotite serpentinization. *Phys Chem Minerals* **47**, 33 (2020).

8. Miller, D. J. & Christensen, N. I. *Seismic Velocities of Lower Crustal and Upper Mantle Rocks from the Slow-Spreading Mid-Atlantic Ridge, South of the Kane Transform Zone (MARK). Proceedings of the Ocean Drilling Program, 153 Scientific Results*. vol. 153 (Ocean Drilling Program, 1997).

9. Bonnemains, D. *et al.* Magnetic signatures of serpentinization at ophiolite complexes. *Geochem Geophys Geosyst* **17**, 2969–2986 (2016).

10. Chen, H. *et al.* Induced Polarization and Magnetic Responses of Serpentinized Ultramafic Rocks From Mid-Ocean Ridges. *JGR Solid Earth* **126**, e2021JB022915 (2021).

11. Cutts, J. A. *et al.* Deducing Mineralogy of Serpentinized and Carbonated Ultramafic Rocks Using Physical Properties With Implications for Carbon Sequestration and Subduction Zone Dynamics. *Geochem Geophys Geosyst* **22**, e2021GC009989 (2021).

12. Chibati, N., Géraud, Y. & Essa, K. S. Petrophysical characterization and thermal conductivity prediction of serpentinized peridotites. *Geophysical Journal International* **231**, 1786–1805 (2022).

13. Manning, C. E. & Ingebritsen, S. E. Permeability of the continental crust: Implications of geothermal data and metamorphic systems. *Reviews of Geophysics* **37**, 127–150 (1999).

14. Longe, P. O., Davoodi, S., Mehrad, M. & Wood, D. A. Combined Deep Learning and Optimization for Hydrogen-Solubility Prediction in Aqueous Systems Appropriate for Underground Hydrogen Storage Reservoirs. *Energy Fuels* **38**, 22031–22049 (2024).

15. Lescoutre, R. *et al.* The fingerprint of structural inheritances in the W-Pyrenees revealed by machine learning detection of seismicity. *Tectonophysics* **903**, 230685 (2025).

16. Lehujeur, M. *et al.* Three-dimensional shear velocity structure of the Mauléon and Arzacq Basins (Western Pyrenees). *BSGF - Earth Sci. Bull.* **192**, 47 (2021).

17. Saspiturry, N., Allanic, C. & Peyrefitte, A. Serpentinization and Magmatic Distribution in a Hyperextended Rift Suture: Implication for Natural Hydrogen Exploration (Mauléon Basin, Pyrenees). *Tectonics* **43**, e2024TC008385 (2024).

18. Cuadrat, J. M. *et al.* Climate of the Pyrenees: Extremes indices and long-term trends. *Science of The Total Environment* **933**, 173052 (2024).

19. Global Heat Flow Data Assessment Group *et al.* The Global Heat Flow Database: Release 2024. https://doi.org/10.5880/FIDGEO.2024.014 (2024) doi:10.5880/FIDGEO.2024.014.

20. Gemant, A. The Thermal Conductivity of Soils. *Journal of Applied Physics* **21**, 750–752 (1950).

21. Clauser, C. & Huenges, E. Thermal Conductivity of Rocks and Minerals. *Rock physics and phase relations: a handbook of physical constants* **3**, 105–126 (1995).

22. O'Hanley, D. S. Solution to the volume problem in serpentinization. *Geol* **20**, 705 (1992).

23. Plümper, O. & Matter, J. Olivine—The Alteration Rock Star. *Elements* **19**, 165–172 (2023).

24. Chogani, A. & Plümper, O. Decoding the nanoscale porosity in serpentinites from multidimensional electron microscopy and discrete element modelling. *Contrib Mineral Petrol* **178**, 78 (2023).

25. Farough, A., Moore, D. E., Lockner, D. A. & Lowell, R. P. Evolution of fracture permeability of ultramafic rocks undergoing serpentinization at hydrothermal conditions: An experimental study. *Geochem Geophys Geosyst* **17**, 44–55 (2016).

26. Tichadou, C. *et al.* Mineralogical and geochemical study of serpentinized peridotites from the North-Western Pyrenees: New insights on serpentinization along magma-poor continental passive margins. *Lithos* **406–407**, 106521 (2021).

27. Choi, J.-H., Edwards, P., Ko, K. & Kim, Y.-S. Definition and classification of fault damage zones: A review and a new methodological approach. *Earth-Science Reviews* **152**, 70–87 (2016).

28. Caine, J. S., Evans, J. P. & Forster, C. B. Fault zone architecture and permeability structure. *Geol* **24**, 1025 (1996).

29. Faulkner, D. R. *et al.* A review of recent developments concerning the structure, mechanics and fluid flow properties of fault zones. *Journal of Structural Geology* **32**, 1557–1575 (2010).

30. Malvoisin, B. & Brunet, F. Water diffusion-transport in a synthetic dunite: Consequences for oceanic peridotite serpentinization. *Earth and Planetary Science Letters* **403**, 263–272 (2014).

31. Furlong, K. P., Villaseñor, A., Benz, H. M. & McKenzie, K. A. Formation and Evolution of the Pacific-North American (San Andreas) Plate Boundary: Constraints From the Crustal Architecture of Northern California. *Tectonics* **43**, e2023TC007963 (2024).

32. Godfrey, N. J., Beaudoin, B. C. & Klemperer, S. L. Ophiolitic basement to the Great Valley forearc basin, California, from seismic and gravity data: Implications for crustal growth at the North American continental margin. *Geological Society of America Bulletin* **109**, 1536–1562 (1997).

33. Langenheim, V. E., Jachens, R. C., Wentworth, C. M. & McLaughlin, R. J. Previously unrecognized regional structure of the Coastal Belt of the Franciscan Complex, northern California, revealed by magnetic data. *Geosphere* **9**, 1514–1529 (2013).
34. Langenheim, V. E., McLaughlin, R. J. & Melosh, B. L. Integrated geologic and geophysical modeling across the Bartlett Springs fault zone, northern California (USA): Implications for fault creep and regional structure. *Geosphere* **20**, 129–151 (2024).
35. National Centers for Environmental Information. Climate at a Glance: California average annual temperature. (2023).
36. Shervais, J. W. *et al.* Multi-Stage Origin of the Coast Range Ophiolite, California: Implications for the Life Cycle of Supra-Subduction Zone Ophiolites. *International Geology Review* **46**, 289–315 (2004).
