# Supplementary material for: Controls on natural hydrogen generation during serpentinization of mantle rocks
Source: Nat Commun. 2026 Jun 11;17:5211. doi: 10.1038/s41467-026-73920-5 (PMC13260452; doi:10.1038/s41467-026-73920-5)
Supplement: Supplementary file 1 — Transparent Peer Review file [file 41467_2026_73920_MOESM1_ESM.pdf]

nature portfolio

Peer Review File

Controls on natural hydrogen generation during serpentinization of mantle rocks

Corresponding Author: Dr Rodolfo Christiansen

This file contains all reviewer reports in order by version, followed by all author rebuttals in order by version.

Attachments originally included by the reviewers as part of their assessment can be found at the end of this file.

Version 0:

Reviewer comments:

Reviewer #1

(Remarks to the Author)
I found this paper to be a highly ambitious and technically rigorous piece of work. It tackles a pressing question - whether natural hydrogen can truly be considered a renewable energy resource - through a sophisticated integration of geophysical inversion, thermodynamic modeling, and petrophysical data. The choice of contrasting case studies (Western Pyrenees and Northern California) is particularly well thought out and makes the findings relevant beyond a local context. At the same time, the manuscript often reads as highly technical and dense, and I worry that some of its impact could be lost on readers who are not experts in serpentinization kinetics or geophysical modeling.

I also was impressed by the scientific novelty and the timeliness of this work. The authors provide a careful and methodologically robust reassessment of hydrogen generation rates, countering previous inflated estimates. The breadth of methods - from 3D structural inversion to stochastic fracture - flow simulations — shows a command of the subject matter that is rare and valuable. I also appreciated the clarity of the figures, especially those that link geological context with model outputs, and the paper’s relevance to the broader debate on energy transition is undeniable.

That said, I did find several weaknesses that should be addressed before publication. First, the manuscript is extremely dense, which makes it less accessible to readers outside of a narrow technical field; at times the sheer amount of detail obscures the main message. Second, while the paper critiques inflated values from previous literature, it sometimes dismisses them too quickly rather than exploring the underlying assumptions that led to such discrepancies. I also found the treatment of uncertainty inconsistent; ranges of production are given, but confidence intervals or explicit error quantification are missing. Finally, the use of key terms like “renewable,” “fossil-type resource,” or “upper bound” sometimes feels too loose for a paper that is otherwise very precise.

If I were to suggest improvements, I would encourage the authors to add a schematic “big picture” diagram that synthesizes the interplay of thermodynamics, kinetics, and geophysical constraints on hydrogen generation. I would also like to see uncertainties presented more explicitly - ideally with sensitivity tests showing which parameters control the models most strongly. The discussion could be strengthened by comparing the modeled production more directly with global analogues, and even a short reflection on economic feasibility would help readers connect the science with its practical implications. I also recommend simplifying some of the longer sentences, harmonizing the terminology, and fixing minor typos (for instance, “498 tonnes/yea” should read “year”).

In conclusion, I believe this is an important and relevant study that deserves to be published, but not in its current form. The science is sound, the data are rich, and the contribution to the energy transition debate is clear, but clarity, uncertainty treatment, and style need revision. For these reasons, my recommendation is minor revisions.

(Remarks on code availability)

Reviewer #2

(Remarks to the Author)
The manuscript entitled, “Natural hydrogen production from serpentinized mantle rocks” by Christiansen et al. reports on some model predictions of the amount of hydrogen that may potentially be generated at two well characterized sites containing ultramafic rocks. The study finds that in the western Pyrenees ~498 tons/year of H_2 is produced from ~630 km^3 of reactive lherzolite. Whereas, in northern California ~346 tons/year is produced from ~4,500 km^3 of highly serpentinized harzburgite. The results of the study indicate that H_2 saturation in fluids, porosity evolution, and reaction kinetics are the primary limiting factors on H2 generation. The authors conclude that natural hydrogen generation is geologically slow and not renewable at industrial scales. The manuscript is well written, and the arguments are generally presented logically. However, the manuscript suffers from a lack of rigor in acknowledging the assumptions and simplifications in the derivation of the model used, and the justification for this approach. I think that there is much more uncertainty about the model results than is acknowledged by the authors. In my opinion, the amount of revision required for the manuscript to be acceptable for publication in Nature Communications is such that it would need ab initio review. Consequently, I recommend that this manuscript not be accepted for publication. Details of the specific issues are described below. I hope that the authors find these comments constructive.

General comments:

1. In my opinion, the major weakness of this manuscript, in its present form, is the emphasis on the magnitude of annual hydrogen generation predicted by the model. I think that there is too much uncertainty in the model construction and inputs to ascribe much validity to these numbers (see details in the subsequent comments). I think that this work demonstrates that the annual generation rate in the western Pyrenees and northern California is likely to be substantially lower than previously reported, but how much lower is still highly uncertain. More important are the factors that influence these results. The model does an excellent job of revealing the impacts of H2 saturation and reaction kinetics on H2 generation rates. The simplifying assumptions that go into the model are unlikely to substantially alter these findings. This should be highlighted as the main conclusion of the study.

2. Text in the abstract [lines 23-24] and introduction [lines 55-59, 62-63] correctly point out that there are many uncertainties associated with natural hydrogen generation and the factors that control it. Despite acknowledging the known uncertainties, this study claims to "provide accurate estimates of hydrogen production constrained by detailed geological and geophysical data." [lines 81-82] I agree with the authors’ assertion that the present study is more detailed than the previous studies of the western Pyrenees that are cited as examples of recent estimates of natural hydrogen production rates [lines 66-69]. However, in my opinion, the model presented in this manuscript is still a highly simplified representation of these very complex natural systems and the results cannot justifiably be considered accurate. That is not to say that the results are not meaningful, but the significance of the work must be presented in a realistic context.

3. H2 generation via water reduction involving Fe-rich minerals in ultramafic rocks can occur at temperatures much higher than 325C as noted on lines 335-337. The text needs to provide some justification for cutting the reaction off at 325C [lines 100-101, 184-185].

4. The use of pure water in the model calculations is a significant simplification of the natural system with profound implications for the results. This fact is completely ignored in the text. There needs to be an acknowledgment that this is a major simplification of the chemistry of the system and some discussion of the potential implications. I do not think that the assertion that this study provides an accurate estimate of the H2 generation in these systems [line 81] can be justified when the model uses pure water.

5. The notion that serpentinization causes an increase in porosity is speculative and unsupported. The precipitation of serpentine minerals during serpentinization fills fractures and reduces porosity. However, the serpentinization process involves hydration of Fe-rich minerals, which is accompanied by a volumetric expansion (up to 40%). This expansion can induce new fractures that increase porosity. I don't think that anyone knows if the net effect is an increase or decrease in porosity. It is probably highly variable.

6. I question the notion of a constant reaction rate over millions of years. I think that it is much more likely that tectonic events open fractures that allow the reaction to proceed. As the peridotite is replaced with serpentinite these fractures become occluded, and the reaction shuts down. A subsequent event then opens fractures again exposing fresh rock and another wave of serpentinization occurs, etc. For the purpose of modeling the system, it is certainly acceptable to make the simplifying assumption of a constant rate, but this should be acknowledged and explained.

7. The dimensions of the modeled fault systems seem to have been chosen based on the dimensions of the mapped ultramafic rock bodies [e.g., line 110]. How geologically realistic is this? This seems to be another simplification of the natural system that likely has profound implications on the results but is glossed over in the text. It seems that a substantial amount of work went into the fracture modeling, but the details are not well explained or justified by citations of previous work.

8. It sounds like the choice of flow rate used in the model was constrained by the geochemistry [lines 114-116]. How realistic is this assumed range of probable flow rates geologically? The introduction states that accurate estimates of hydrogen generation are constrained by geologic and geophysical data, yet there does not appear to be any geologic data behind the choice of the range of flow rates examined. Again, these sorts of simplifying assumptions are certainly acceptable, but they must be acknowledged as such. In reality, the flow rate has likely fluctuated over the millions of years that these rocks have been in place.

9. I have doubts about the appropriateness of some of the input data that was used to build the model. Line 106 cites ref 29 (García-Pintado et al., 2025) for the thermodynamic database used in the study. However, the methods section of the manuscript seems to point to refs 49 and 50 (Holland & Powell 1998; 2011). I do not have access to a copy of García-Pintado et al., (2025) to check, but this publication does not seem to be an appropriate source of thermodynamic data. If this publication cites other sources for the thermodynamic data, the original source(s) should be cited. The range of values used for constraining the geometry and structure of the fracture networks are said to be based on sampling of key parameters [lines 403-415], yet there are no citations for the source of this information (except for one citation for permeability). The data used to calculate H2 saturation in water is from ref 62, but this published model is only valid from 0 to 100° C. There are better models out there such as Longe et al., 2024, which covers a much broader range of temperatures.

10. Additional lesser comments are included in the annotated copy of the manuscript.

References
García-Pintado, J. & Pérez-Gussinyé, M. Shallow peridotites at magma-poor rifted margins: occurrences, serpentinization, and H2 generation. Natural Hydrogen Systems: Properties, Ocurrences, Generation Mechanisms, Exploration, Storage and 580 Transportation. (R. Rezaee & B. Evans, Eds) De Gruyter Academic Publishing. (2025) 581 doi:https://doi.org/10.1515/9783111437040-005.
Holland, T. J. B. & Powell, R. An improved and extended internally consistent thermodynamic dataset for phases of petrological interest, involving a new equation of state for solids: THERMODYNAMIC DATASET FOR PHASES OF PETROLOGICAL 637 INTEREST. Journal of Metamorphic Geology 29, 333–383 (2011).
Holland, T. J. B. & Powell, R. An internally consistent thermodynamic data set for phases of petrological interest. Journal Metamorphic Geology 16, 309–343 (1998).
Longe, P. O., S. Davoodi, M. Mehrad, and D. A. Wood, 2024, Combined Deep Learning and Optimization for Hydrogen-Solubility Prediction in Aqueous Systems Appropriate for Underground Hydrogen Storage Reservoirs: Energy & Fuels, v. 38, no. 22, p. 22031–22049, doi:10.1021/acs.energyfuels.4c03376.

(Remarks on code availability)

Version 1:

Reviewer comments:

Reviewer #1

(Remarks to the Author)
I have had the opportunity to review the responses submitted to both reviewers. I must acknowledge the work done. The present manuscript remains publishable because its distinctive contribution is the combination of 3D geophysical inversion, thermo-hydro-chemical constraints, pressure-dependent H_2 solubility, and explicit uncertainty/sensitivity analysis across two contrasting mantle systems, rather than one regional conceptual argument alone. But the authors should state this novelty more crisply in the introduction and discussion; otherwise, readers may see the paper as mainly a downward revision of prior Pyrenean estimates rather than as a broader methodological advance. The paper also appropriately situates its normalized fluxes against recent observations from Bulqizë and Zambales, which helps, but that comparison should remain cautious because seep fluxes are not identical to generation rates.

The uncertainty/sensitivity concern is satisfactorily addressed; the solubility criticism is substantially addressed; the citation problem for thermodynamic and fracture-network inputs is addressed; and the framing of previous inflated Pyrenean estimates is improved. The “big picture” schematic requested was not added; I do not regard that as fatal, although one synthetic conceptual figure would still help. I do insist in this improvement. The request for economic feasibility was declined; that is acceptable for this journal, because the paper is fundamentally a geoscience/process paper, not a techno-economic assessment. More importantly, the paper still needs a slightly tighter editorial separation between what is demonstrated and what is inferred. The revised text does this much better than the original, but not perfectly. For example, the introduction and discussion now present the model as quasi-dynamic and first-order, yet the case-study numbers remain visually prominent and may still invite over-interpretation unless the title, abstract, and conclusions are disciplined consistently.

In summary, I do advise to accept this paper after the previous requested improvements.

(Remarks on code availability)

Reviewer #2

(Remarks to the Author)
I find the revised manuscript to be excellent. This work represents an important contribution to our understanding of the occurrence of natural hydrogen in the Earth's crust. A few minor suggested corrections are included in the annotated copy of the revised manuscript.

(Remarks on code availability)

Open Access This Peer Review File is licensed under a Creative Commons Attribution 4.0 International License, which permits use, sharing, adaptation, distribution and reproduction in any medium or format, as long as you give appropriate credit to the original author(s) and the source, provide a link to the Creative Commons license, and indicate if changes were made.
In cases where reviewers are anonymous, credit should be given to 'Anonymous Referee' and the source.
The images or other third party material in this Peer Review File are included in the article's Creative Commons license, unless indicated otherwise in a credit line to the material. If material is not included in the article's Creative Commons license and your intended use is not permitted by statutory regulation or exceeds the permitted use, you will need to obtain permission directly from the copyright holder.
To view a copy of this license, visit https://creativecommons.org/licenses/by/4.0/

ASNWERS TO REVIEWER COMMENTS

We thank the reviewers for their constructive and thoughtful comments, which have significantly improved the quality, clarity, and robustness of the manuscript. In response, we revised the text throughout to better align the presentation of the model with its underlying assumptions and limitations. We implemented an explicit uncertainty quantification framework based on Monte Carlo simulations, complemented by a univariate sensitivity analysis (both now described in the supplementary material), allowing uncertainties in input parameters to be propagated directly into hydrogen production estimates and enabling a clear view of what are the main parameters affecting the results. We also recalculated the H_2 solubility using a physically consistent, pressure-temperature dependent thermodynamic approach based on experimental data and including fugacity corrections, making the estimates more robust over the full pressure-temperature range considered. In the course of revising the numerical implementation, we identified and corrected a bug in the code whereby the amount of water available for serpentinization (particularly at very high flow rates) was inadvertently overestimated. This correction leads to a different behaviour only at unrealistically high flow rates, but does not affect the main conclusions of the study, as such flow conditions lie well outside the range considered geologically plausible. Furthermore, we upload the code to GitHub and made it publicly available including the data to reproduce our reults.

Reviewer #1 (Remarks to the Authors):

1. I worry that some of its impact could be lost on readers who are not experts in serpentinization kinetics or geophysical modeling. The manuscript is extremely dense, which makes it less accessible to readers outside of a narrow technical field; at times the sheer amount of detail obscures the main message.

We recognize that the level of technical detail may limit accessibility for non-specialist readers. To address this, we revised the manuscript to clarify and highlight the main message. These changes can be found in the final paragraphs of the Introduction, where the main objectives and conclusions are stated more explicitly, and in the first two paragraphs of the Discussion, which were rewritten to clearly highlight the key controlling processes.

2. Second, while the paper critiques inflated values from previous literature, it sometimes dismisses them too quickly rather than exploring the underlying assumptions that led to such discrepancies

We acknowledge that earlier estimates were previously discussed too briefly. In the revised manuscript, we now examine the assumptions underlying these high production values, including implicit assumptions of unlimited water supply and fluid renewal, as well as the neglect of hydrogen saturation and kinetic limitations. We also clarify that these studies estimate "cumulative theoretical potential" (wrongly translated into production rates) rather than sustained production rates and directly compare them with results from our unconstrained model. These changes are implemented in the Introduction and Discussion.

3. I also found the treatment of uncertainty inconsistent; ranges of production are given, but confidence intervals or explicit error quantification are missing.

We have revised the model to account for uncertainty through Monte Carlo simulations, including a statistical treatment of rate outputs (standard deviation based on the runs). This approach propagates uncertainties in input parameters directly into hydrogen production estimates, rather than relying on end-member scenarios based on serpentinization front velocity like before (min, avg, and max). As a result, production rates are now reported as a single value for each temperature range with the corresponding standard deviation derived from the Monte Carlo runs. In addition, we implemented a univariate sensitivity analysis, in which individual parameters are varied independently within their uncertainty ranges while all others are held constant (described in detail in our response to Question 6). These methodological updates are described in the Methods section ("Uncertainty quantification and sensitivity analysis"), presented in the Results section ("Model sensitivity"), and summarized in the Supplementary files and tables, which report both Monte Carlo statistics and univariate sensitivity results.

4. The use of key terms like "renewable," "fossil-type resource," or "upper bound" sometimes feels too loose for a paper that is otherwise very precise.

We agree that clearer terminology was needed. In the revised manuscript, qualitative labels such as "renewable" or "fossil-type resource" have been removed from the scientific interpretation, and terminology has been harmonized throughout the Discussion and Conclusions.

5. I would encourage the authors to add a schematic "big picture" diagram that synthesizes the interplay of thermodynamics, kinetics, and geophysical constraints on hydrogen generation.

We agree that a clear synthesis of the controlling processes is important. In the revised manuscript, this role is fulfilled by Figs. 2 and 4, which together show that hydrogen generation is controlled by (i) the amount of water supplied, (ii) hydrogen saturation in the fluid, and (iii) the rate at which serpentinization is affecting the rocks. We therefore did not add an additional schematic figure, as these controls are already directly expressed in the quantitative results and are site dependent. In addition, we simplified the terminology for non-specialist readers and now use for example "rate-limited" instead of "thermodynamic constraint".

6. I would also like to see uncertainties presented more explicitly - ideally with sensitivity tests showing which parameters control the models most strongly.

We addressed this issue through the implementation of Monte Carlo uncertainty propagation and a univariate sensitivity analysis, which together quantify model uncertainty and identify the parameters controlling hydrogen production. These additions are described in the Methods, subsection "Uncertainty quantification and sensitivity analysis", presented in the Results, subsection "Model sensitivity", and summarized in the Supplementary material (see response to Question 3 for details).

7. The discussion could be strengthened by comparing the modeled production more directly with global analogues, and even a short reflection on economic feasibility would help readers connect the science with its practical implications.

We agree that broader comparisons and economic considerations are useful for placing model results in a wider context. However, our intention is to base the Discussion strictly on the data and results presented in this study. Introducing global analogues or an economic assessment would open a new line of discussion that goes beyond the primary aim of the paper and is not directly supported by the modelling framework. That said, the revised Discussion does place our results in a broader physical context by addressing the conditions required for hydrogen accumulation. In particular, we discussed now that hydrogen generation is predominantly aqueous, that production does not necessarily lead to accumulation, and that prospectivity is controlled mainly by migration, phase separation, and trapping efficiency rather than by generation rates alone. These points follow directly from the model results and are therefore discussed without extending beyond the scope of the present study.

8. I also recommend simplifying some of the longer sentences, harmonizing the terminology, and fixing minor typos (for instance, “498 tonnes/yea” should read “year”).

The manuscript has been carefully edited to improve clarity, consistency, and overall readability, and minor typographical issues have been corrected.

Reviewer #2 (Remarks to the Authors):

1. The major weakness of this manuscript is the emphasis on the magnitude of annual hydrogen generation predicted by the model. I think that there is too much uncertainty in the model construction and inputs to ascribe much validity to these numbers (see details in the subsequent comments). I think that this work demonstrates that the annual generation rate in the western Pyrenees and northern California is likely to be substantially lower than previously reported, but how much lower is still highly uncertain. More important are the factors that influence these results. The model does an excellent job of revealing the impacts of H2 saturation and reaction kinetics on H2 generation rates. The simplifying assumptions that go into the model are unlikely to substantially alter these findings. This should be highlighted as the main conclusion of the study.

We agree that absolute annual hydrogen generation rates remain uncertain and should not be interpreted as precise in situ values. This point is now addressed in the revised manuscript. We clarify that the reported production rates represent “first-order magnitudes”, and that mantle-derived natural hydrogen behaves as a slowly replenished geological resource at large scales. Moreover, the emphasis of the revised Discussion is placed on order-of-magnitude behaviour and on the physical controls governing hydrogen generation, rather than on exact numerical values. In particular, the main conclusion of the study highlights the dominant roles of fluid availability, hydrogen saturation, and reaction kinetics in limiting hydrogen generation. Within this framework, the average production rates obtained (now presented as rate per kg of rock) are shown to be consistent with normalized hydrogen fluxes inferred from other well-documented crustal settings,

supporting the interpretation that the model captures the correct order of magnitude of natural hydrogen generation while also identifying its first-order controls.

9. The model presented in this manuscript is still a highly simplified representation of these very complex natural systems and the results cannot justifiably be considered accurate. That is not to say that the results are not meaningful, but the significance of the work must be presented in a realistic context.

We agree that the model necessarily represents a simplified view of complex natural systems and that the results should not be interpreted as accurate in situ predictions (see Answer 1). In the revised manuscript, we now address this by discussing the main first-order assumptions underlying the modelling framework and their implications for hydrogen production estimates. We clarify that hydrogen generation is treated as a quasi-dynamic, time-averaged process and that several simplifying assumptions (such as pure-water conditions, homogeneous fluid access, and restriction to magnetite-related reactions) affect predicted rates. As a result, the derived production rates are framed as first-order, order-of-magnitude estimates rather than precise values.

2. H2 generation via water reduction involving Fe-rich minerals in ultramafic rocks can occur at temperatures much higher than 325C as noted on lines 335-337. The text needs to provide some justification for cutting the reaction off at 325C.

In the revised manuscript, we clarify that the temperature cutoff at 300-350°C (avg 325 °C) does not represent a general limit to hydrogen generation, but rather reflects the specific reaction pathway considered in the model. Our thermodynamic calculations account only for hydrogen production associated with magnetite formation during serpentinization (now explicitly informed in the manuscript). Above ~325 °C, magnetite stability and serpentinization reactions involving magnetite become progressively limited (additional hydrogen-generating pathways are not included in the present modelling framework). This clarification is now stated in the text to avoid confusion between the temperature range of the modelled reaction and the broader range of conditions under which hydrogen generation may occur in natural ultramafic systems.

3. The use of pure water in the model calculations is a significant simplification of the natural system with profound implications for the results. This fact is completely ignored in the text. There needs to be an acknowledgment that this is a major simplification of the chemistry of the system and some discussion of the potential implications.

This point is now acknowledged in the revised manuscript. We clarify that thermodynamic calculations in the database are performed using pure water (probably further refinements will include not salinity). We further discuss that natural fluids commonly exhibit elevated salinity and reduced water activity, which experimental studies indicate can significantly slow serpentinization reaction rates. As such, the use of pure water systematically biases hydrogen production toward higher values, and the resulting production rates should therefore be interpreted as first-order estimates within the defined reaction framework.

10. The notion that serpentinization causes an increase in porosity is speculative and unsupported. The precipitation of serpentine minerals during serpentinization fills fractures

and reduces porosity. However, the serpentinization process involves hydration of Fe-rich minerals, which is accompanied by a volumetric expansion (up to 40%). This expansion can induce new fractures that increase porosity. I don't think that anyone knows if the net effect is an increase or decrease in porosity. It is probably highly variable.

We agree that the net effect of serpentinization on porosity is uncertain and likely highly variable and the relative importance of these processes is not well constrained and likely depends on local conditions. In our modelling framework, we therefore do not assume a bulk or long-term increase in porosity. Instead, we assume that transient porosity is locally generated at the serpentinization reaction front, facilitating water access to reactive minerals (e.g., Chogani & Plümper, 2023). This assumption does not imply that porosity is conserved or increases throughout the rock volume after reaction. Rather, only the reaction front is considered water-filled, while the surrounding rock remains neutral in the model. This clarification has now been stated in the Methods section ("Hydrogen production estimation"), where we emphasize that serpentinization is associated with localized and transient porosity changes that may enhance water inflow at the reaction front.

4. I question the notion of a constant reaction rate over millions of years. I think that it is much more likely that tectonic events open fractures that allow the reaction to proceed. As the peridotite is replaced with serpentinite these fractures become occluded, and the reaction shuts down. A subsequent event then opens fractures again exposing fresh rock and another wave of serpentinization occurs, etc. For the purpose of modeling the system, it is certainly acceptable to make the simplifying assumption of a constant rate, but this should be acknowledged and explained.

We agree that serpentinization and associated hydrogen generation are unlikely to proceed at a constant rate over millions of years and are more realistically controlled by episodic fracture opening and sealing. This point is now acknowledged and discussed, in the paragraph beginning "The present scenario likely does not apply to the earliest stages of serpentinization". There, we clarify that the modelled serpentinization rates should be interpreted as effective, time-averaged rates, representative of periods with sustained fluid access and enhanced fracturing, rather than as continuous long-term reaction rates. We further state that in natural systems, fluid supply and permeability are expected to be intermittent and spatially localized, with elevated reaction rates occurring during short-lived fracture-controlled fluid-infiltration events separated by longer inactive periods.

5. The dimensions of the modeled fault systems seem to have been chosen based on the dimensions of the mapped ultramafic rock bodies [e.g., line 110]. How geologically realistic is this? This seems to be another simplification of the natural system that likely has profound implications on the results but is glossed over in the text. It seems that a substantial amount of work went into the fracture modeling, but the details are not well explained or justified by citations of previous work.

The geological realism and the intentionally simplified nature of the fault-system geometry are now clarified in the Methods section, subsection "Deep Water Flow Capacity Estimation". We state that fault geometries are parameterized to match the mapped extent of ultramafic bodies and

are not intended to represent detailed fault architectures. Instead, fault length, damage-zone thickness, fracture density, permeability and hydraulic connectivity are sampled stochastically over physically plausible ranges, based on published constraints for fractured and faulted crust. As a result, individual realizations rarely reproduce fault geometries that coincide exactly with the full extent of the ultramafic bodies, as the model is intentionally designed to explore a range of admissible configurations. However, when considered altogether, these parameters can lead to hydraulic conditions that allow the range of water fluxes considered in this study (green-shaded domains in Figs. 2 and 4) to be achieved. These flux ranges were tested using the Monte Carlo framework and correspond to flow conditions that can be sustained in at least 70% of the realizations. Accordingly, the flow values derived for fault-controlled transport should be interpreted as an upper-bound estimate, rather than as a unique or directly realizable flow rate for the study area.

6. It sounds like the choice of flow rate used in the model was constrained by the geochemistry [lines 114-116]. How realistic is this assumed range of probable flow rates geologically? The introduction states that accurate estimates of hydrogen generation are constrained by geologic and geophysical data, yet there does not appear to be any geologic data behind the choice of the range of flow rates examined. Again, these sorts of simplifying assumptions are certainly acceptable, but they must be acknowledged as such. In reality, the flow rate has likely fluctuated over the millions of years that these rocks have been in place.

The assumed range of flow rates are now justified and discussed in the Results (Western Pyrenees and Northern California subsections; Figs. 2b and 4b) and in the Methods, subsection “Deep Water Flow Capacity Estimation”. We clarify that the explored flow ranges represent time-averaged effective fluxes, bounded on the lower end by the minimum sustained flux required to achieve the observed degrees of serpentinization over geological timescales, and on the upper end by the maximum transport capacity of fault systems inferred from permeability-based Monte Carlo simulations based on geology (see answer 5). We acknowledge that natural flow rates likely fluctuated through time and that the adopted values are simplifying, time-averaged representations, rather than constant geological fluxes.

7. I have doubts about the appropriateness of some of the input data that was used to build the model. Line 106 cites ref 29 (García-Pintado et al., 2025) for the thermodynamic database used in the study. However, the methods section of the manuscript seems to point to refs 49 and 50 (Holland & Powell 1998; 2011). I do not have access to a copy of García-Pintado et al., (2025) to check, but this publication does not seem to be an appropriate source of thermodynamic data. If this publication cites other sources for the thermodynamic data, the original source(s) should be cited.

We state that thermodynamic equilibrium data are derived from the Holland & Powell framework using Theriak-Domino, and clarify that García-Pintado et al. is cited as the source compiling and applying these calculations, while the original thermodynamic datasets are now referenced.

8. The range of values used for constraining the geometry and structure of the fracture networks are said to be based on sampling of key parameters [lines 403-415], yet there are no citations for the source of this information (except for one citation for permeability).

The ranges used to describe fracture-network geometry and hydraulic properties are now fully documented in the Methods subsection "Deep Water Flow Capacity Estimation". Specifically, fault length, damage-zone thickness, fracture spacing, fracture density, permeability, and hydraulic connectivity are sampled stochastically within physically plausible ranges derived from published studies of fractured and faulted crust (Choi et al., 2016; Caine et al., 1996; Faulkner, 2010; Manning and Ingebritsen, 1999). Citations have been added for each parameter class (e.g., permeability, fracture spacing, and fault-zone structure), and we clarify that these ranges are intended to represent first-order geological variability rather than site-specific fault architectures.

9. The data used to calculate H2 saturation in water is from ref 62, but this published model is only valid from 0 to 100° C. There are better models out there such as Longe et al., 2024, which covers a much broader range of temperatures.

We revised the treatment of H_2 solubility and developed an explicit, data-driven solubility model based on experimental measurements. After several attempts to obtain the solubility database created by Longe et al. were unsuccessful, we reconstructed the solubility model directly from the publicly available experimental data reported in that study. These data consist of measured H_2 mole fractions in pure water (we cleaned saline solutions to ensure consistency with our modelling). Solubility was modelled using the Krichevsky-Kasarnovsky formulation. Fugacity corrections were applied using a Peng-Robinson equation of state for pure H_2. Importantly, this update does not represent a re-fitting of a pressure-solubility curve. Rather, the previous linear approximation has been replaced by a pressure-temperature dependent thermodynamic formulation, calibrated against the full experimental dataset and evaluated point-wise at the pressure and temperature of each measurement. Figure 1 illustrates the effect of this reformulation by comparing solubility estimates obtained using the previously adopted linear model with those derived from the revised Krichevsky-Kasarnovsky formulation.

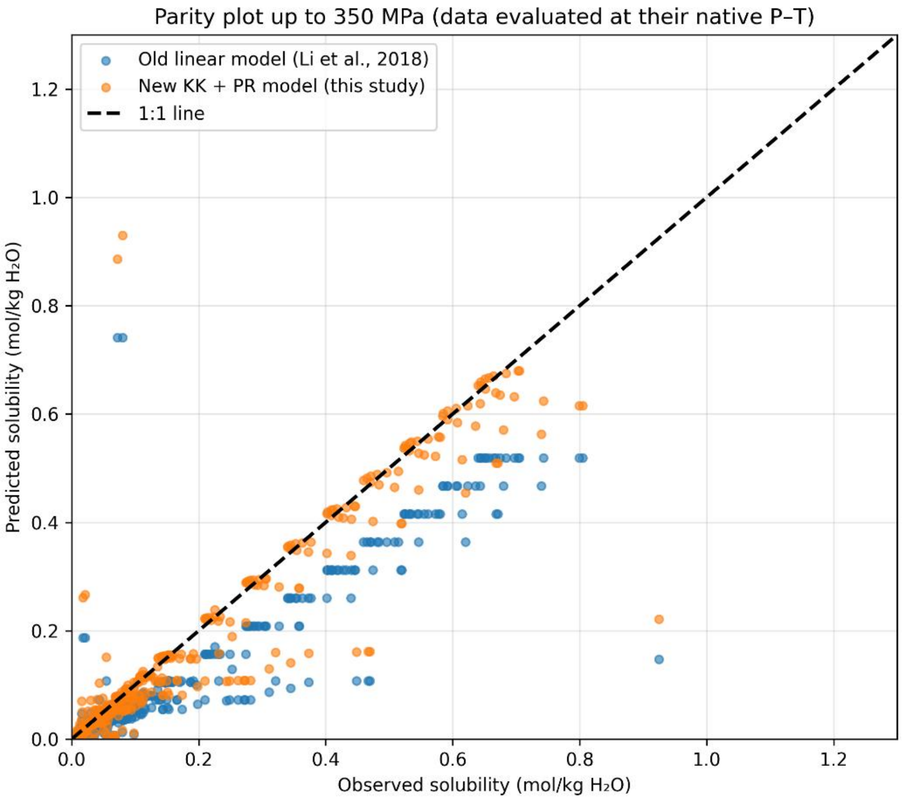

Fig. 1: Comparison of H_2 solubility models. Parity plot comparing experimental H_2 solubilities with predictions from the previously used linear approximation and the revised Krichevsky-Kasarnovsky formulation.

ASNWERS TO REVIEWER COMMENTS

We thank the reviewers for their thoughtful comments. In response, we revised the text accepting the corrections of both reviewers.

Reviewer #1 (Remarks to the Author):

1- The authors should state this novelty more crisply in the introduction and discussion; otherwise, readers may see the paper as mainly a downward revision of prior Pyrenean estimates rather than as a broader methodological advance.

We agree with this assessment. The title, abstract, and discussion have been revised to foreground the methodological contribution of the work more clearly. Please refer to the annotated manuscript for the specific changes.

2- The paper also appropriately situates its normalized fluxes against recent observations from Bulqizë and Zambales, which helps, but that comparison should remain cautious because seep fluxes are not identical to generation rates.

We thank the reviewer for this important clarification. The following sentence has been added immediately after the comparison: *"Although seep fluxes are not direct proxies for generation rates, this agreement suggests that the model captures the correct order of magnitude of natural hydrogen generation systems.*

3- The "big picture" schematic requested was not added; I do not regard that as fatal, although one synthetic conceptual figure would still help. I do insist in this improvement.

We considered the possibility of including a conceptual schematic, but concluded that no single figure can faithfully represent the controls on hydrogen generation during serpentinization, as these are inherently site-dependent. In the Western Pyrenees, generation is primarily limited by H_2 solubility in the fluid, while in Northern California the dominant control is the reaction rate, a fundamentally different regime. A universal diagram would risk obscuring this contrast, which is one of the central findings of our study.

4- More importantly, the paper still needs a slightly tighter editorial separation between what is demonstrated and what is inferred. The revised text does this much better than the original, but not perfectly. For example, the introduction and discussion now present the model as quasi-dynamic and first-order, yet the case-study numbers remain visually prominent and may still invite over-interpretation unless the title, abstract, and conclusions are disciplined consistently.

We agree. The title, abstract, and discussion have been revised to consistently frame the results as estimations rather than precise in-situ measurements. Please refer to the annotated manuscript for the specific changes.

Reviewer #2 (Remarks to the Author):

5- A few minor suggested corrections are included in the annotated copy of the revised manuscript

We thank Reviewer #2 for the careful editorial review. All suggested corrections have been accepted and incorporated into the revised manuscript.

Natural hydrogen production from serpentinized mantle rocks

Rodolfo Christiansen[1]*, Mohamed Sobh[1], Christian Ostertag-Henning[2], Guido Gianni[3], Nicolas Saspiturry[4], Sebastien Chevrot[5,6], Victoria Langenheim[7], Javier Garcia-Pintado[8], Gerald Gabriel[1,9]

[1] LIAG Institute for Applied Geophysics, 30655 Hannover, Germany
[2] Federal Institute for Geosciences and Natural Resources (BGR), 30655 Hannover, Germany
[3] Czech Academy of Sciences, Institute of Geophysics, 14100 Prague, Czechia
[4] Géosciences Montpellier, Université de Montpellier, CNRS, 34095 Montpellier, France
[5] Université de Toulouse, UPS-OMP, IRAP, 31028 Toulouse, France
[6] GET, CNRS, Observatoire Midi Pyrénées, Université de Toulouse, UMR5563, F-31400 Toulouse, France
[7] U.S. Geological Survey, Moffett Field, California 94035, USA
[8] MARUM - Center for Marine Environmental Sciences, University of Bremen, 28359 Bremen, Germany
[9] Leibniz University Hannover, Institute of Earth System Sciences, 30167 Hannover, Germany

Abstract

Mantle rocks undergoing serpentinization at upper-crustal levels can generate significant amounts of natural hydrogen. Although the potential for substantial hydrogen generation during fluid-rock reactions of these rocks is documented by lab experiments and occurrences of hydrogen-rich natural gas seeps, the exact production rates and the factors limiting production remain poorly understood. Here, we demonstrate that in two mantle rock systems with distinct compositions, the fertile lherzolites of the Western Pyrenees and the depleted harzburgites of Northern California, continuous fluid infiltration may sustain hydrogen generation, but only at rates of approximately 500 tonnes per year. Furthermore, by integrating three-dimensional geophysical inversion with a semi-coupled thermo-hydro-chemical model, we show that the main limiting factors controlling hydrogen yield are H_2 saturation in the fluid, porosity evolution at the reaction front, and reaction kinetics. The generation rates determined within the physico-chemical limits suggest that, although natural hydrogen from mantle rock serpentinization is a clean energy source, it may not be renewable on a large scale. Instead, its generation times for substantial accumulations more closely resemble those of fossil-type resources.

1. Introduction

Serpentinization is an abiotic reaction involving the hydration of ultramafic rocks, in which Fe(II) in primary minerals is oxidized to Fe(III) in secondary phases, reducing hydrogen atoms in water molecules to molecular hydrogen (H_2). This process is one of the primary natural mechanisms for hydrogen generation [1–5]. The generation rate of molecular hydrogen (hereafter referred to as hydrogen or H_2) and the rate of serpentinization are highly dependent on temperature, with maximum rates between 200°C and 300°C [2,6]. This mineral replacement reaction also affects the petrophysical properties of the rocks by decreasing density and seismic velocities, concomitant most often with increasing magnetic susceptibility, allowing serpentinites to be distinguished from fresh mantle rocks based on their geophysical characteristics, such as gravity, magnetic, and seismic properties[7–9]. In the past years natural hydrogen has emerged as a promising source of clean energy [10]. However, more detailed investigations suggests that it may not meet the criteria to be considered a renewable resource at an industrial scale, as preliminary estimates indicate that its generation occurs over geological timescales, with fluxes limited by thermodynamic and kinetic constraints even in the most favourable conditions such as deep olivine serpentinization [11,12]. Major obstacles to quantifying natural hydrogen generation include the inaccessibility of deep crustal environments, a limited understanding of water availability for the serpentinization reaction, and uncertainties in reaction kinetics within the source rocks, especially for low water-to-rock ratios.

Serpentinization is associated with some of the largest discoveries of natural occurrences of hydrogen worldwide, with measured flow rates of several hundred tonnes per year and high hydrogen concentrations in emanating gases [13,14]. However, model-based estimates remain highly uncertain. For example, in the Western Pyrenees, reported values range from several hundred thousand to several million tonnes of hydrogen produced per year [15,16], orders of magnitude higher than hydrogen flows observed in comparable geological settings worldwide. Thus, these estimates remain under debate and likely overstate actual rates due to oversimplified assumptions. Key limiting processes, such as saturation of H_2 in the fluid in gas-fluid-rock equilibria, restricted porosity development, and limited fluid renewal are often omitted in models. Here, we present an open source model for quantifying natural H_2 generation via serpentinization, which considers geological parameters within precise petro-physico-chemical boundary conditions. The model accounts for fluid saturation and

incorporates thermodynamic and rock-specific petrophysical properties, including mineral-bound water uptake, pressure dependent hydrogen solubility, water renewal rates, and porosity-controlled reaction front propagation velocity. Building on these capabilities, we have analysed the serpentinization processes in two key areas, the Western Pyrenees and Northern California, selected because of good data availability and for their contrasting mantle rock compositions. The first case involves relatively fertile mantle rocks rich in olivine and orthopyroxene, whereas the second is dominated by less reactive rocks that reflect residues of high degrees of partial melting. Through these case studies, we examine the local parameters governing natural hydrogen generation, assess the volume of water that can infiltrate the ultramafic source rocks, and provide accurate estimates of hydrogen production constrained by detailed geological and geophysical data. Taken together, our results provide a reassessment of natural hydrogen derived from mantle rocks, which, on large scales, behaves as a slowly replenished resource.

Results

Western Pyrenees. The Western Pyrenees (Fig. 1a, b) consist of a tectonic wedge, where mantle-derived rocks, mainly spinel lherzolites [17,18], were exhumed during mid-Cretaceous rifting [19,20] and tectonically uplifted to their current shallow position (~10 km) as a result of Eocene-Oligocene convergence, which ended about 25 Ma ago [21–24]. Since then, tectonic conditions have remained relatively stable, which provides a prolonged window that keeps the exhumed mantle rocks within optimum pressure-temperature conditions for serpentinization [15,16,25]. This process is further supported by the occurrence of microseismicity around a high-velocity mantle body, which is interpreted as evidence of ongoing hydration [26,27]. Evaporites and claystones within the overlying sedimentary sequence may act as a trap for the hydrogen being produced [28]. Soil gas measurements (Fig. 1b) confirmed that hydrogen seeps near major thrust faults, suggesting that it migrates to the surface [22]. Gravity and magnetic anomaly maps align with a body of variably serpentinized lithospheric mantle at an unusual shallow depth [25].

Geophysical inversion of gravity and magnetic data (Fig. 1c, d), constrained by seismic velocity models [27], and combined with thermal modelling (Fig. 1d), indicates the presence of ~2,700 km^3 of ultramafic material serpentinized over ~37–57% of its volume (Fig. 1e). Of this volume, 630 km^3 are capable of generating hydrogen, while ~2,100 km^3 occur outside the hydrogen production range (> 325°C). These rocks are spatially associated with a second group of ~2,400 km^3 of mantle rocks characterized by a minimal serpentinization of ~10% (green unit in Fig. 1c and Fig. 1e, with volume limited by the 20 km depth extent of the model). These two

groups, moderately serpentinized rocks and weakly altered mantle, are partially interlayered or superimposed at depth. The maximum amount of H_2 that these rocks can produce was assessed using a thermodynamic database [29] designed for lherzolite phase equilibria with pure water (Fig. 2a).

The possible range of water input for the rocks volume (shaded area in Fig. 2b) was constrained on one end by the maximum transport capacity of a 50 km fault zone (see Methods and Fig. 7), selected to match the lateral extent of the ultramafic unit, and on the other end by the flow required to serpentinize 47% of the mantle rock volume over 25 Ma. An intermediate flow rate of 5×10^5 L/day was selected as a representative scenario, yielding a water-to-rock ratio (W/R) of 0.16, based on the amount of water interacting with the rock relative to the mass affected daily by the advancing serpentinization front. This rate ensures sufficient hydrogen generation while avoiding unrealistically rapid depletion of the reactive rock or, conversely, negligible production due to insufficient fluid input. Considering the entire range of probable flow rates, annual hydrogen production, as dissolved H_2, could be in the range of ~100 to ~2,000 tonnes. Narrowing to the selected intermediate flow rate, predicted output ranges from 314 to 965 tonnes/year, with an average of 498 tonnes/yea (Fig. 2a, b). The results also indicate that the primary limiting factor on hydrogen production under this flow of water is the rapid saturation of hydrogen in the fluid phase, caused by inefficient removal of the reacted fluids, which suppresses further hydrogen-generating reactions due to thermodynamic constraints (Fig. 2b).

Additional simulations were carried out to test hydrogen generation (with a fixed W/R = 0.16) across a range of fluid flows from 1 to 10^{10} L/day (Fig. 2b). Water residence time, defined as the duration water remains in the reactive zone, depends on flow rate and pore volume. When flow is low, water renewal is minimal, leading to long residence times. At very low flows ($<10^3$ L/day), water availability is the limiting factor and production rates remain below ~10 tonnes/year (adopted reference threshold). At moderate flows (~10^3–10^7 L/day), residence time decreases but still allows dissolved H_2 to accumulate to saturation, which inhibits further hydrogen-generating reactions due to thermodynamic limits on product removal. At higher flows ($>10^7$ L/day), residence time becomes very short and although water supply is sufficient to transport hydrogen away, overall production is limited by reaction kinetics. As a result, even under very high water-flow conditions, average hydrogen production is limited to ~30,000 tonnes/year for this area (Fig. 2b).

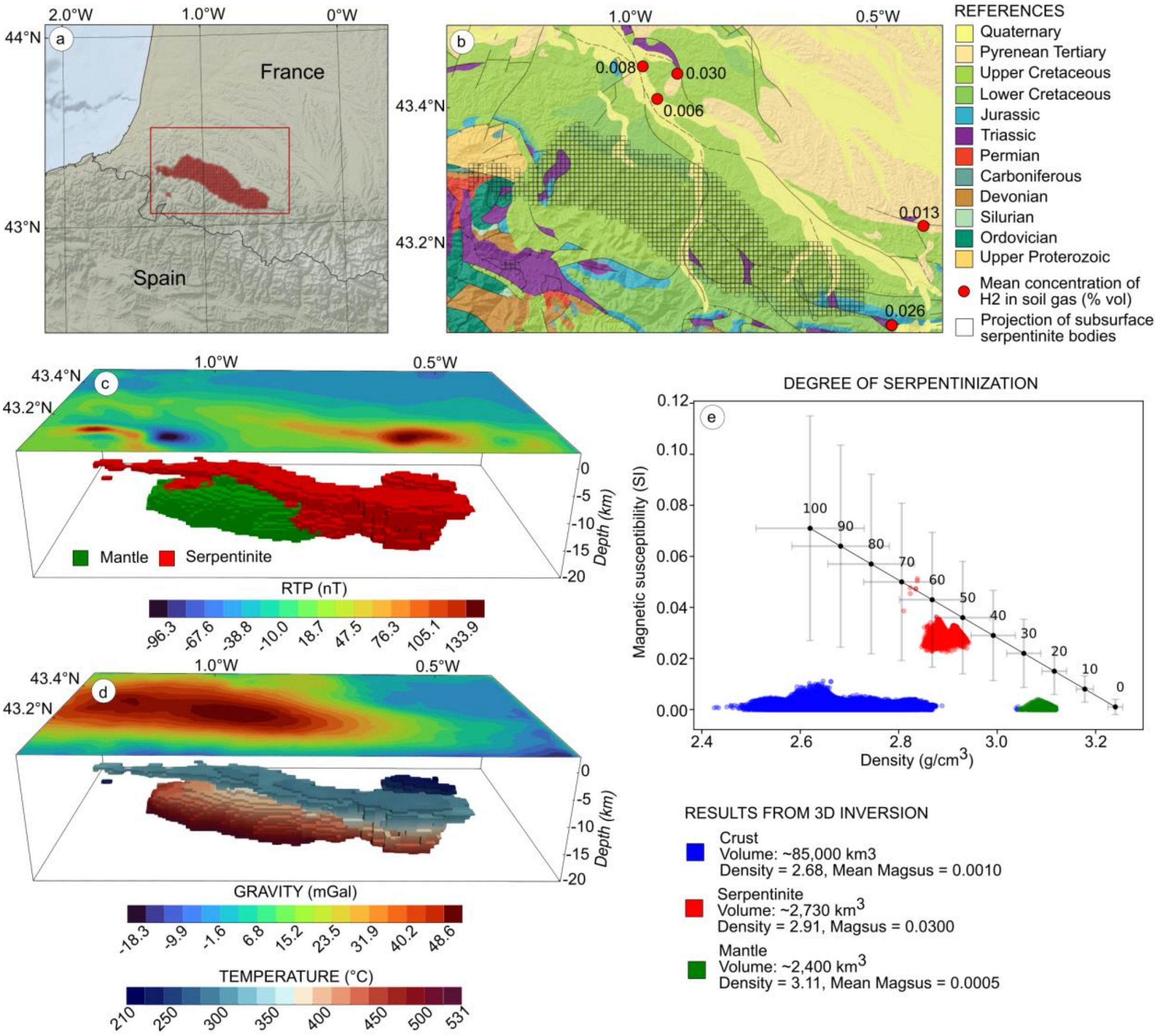

Fig. 1: Results of source rock volumes for the Western Pyrenees. a) Index map of study area (red box). The red polygons shows modelled serpentinite extent projected to the surface. b) Age of geologic units in the study area (modified from ref. 30). Polygons show modelled serpentinite blocks projected to the surface. Red circles represent H2 measurements (%) in soil gases (modified from ref. 22). c) Reduced to the pole magnetic anomalies (RTP) for the area (modified from ref. 31 and ref. 25) with 3D blocks showing rock classification based on joint inversion of gravity and magnetic data. d) Residual isostatic gravity anomalies for the area (modified from ref. 25) with 3D blocks showing temperature distribution. e) Results of the Gaussian Mixture Model (GMM) indicating the degree of serpentinization based on density and magnetic susceptibility results. Values are projected to the closest serpentinization point in the line.

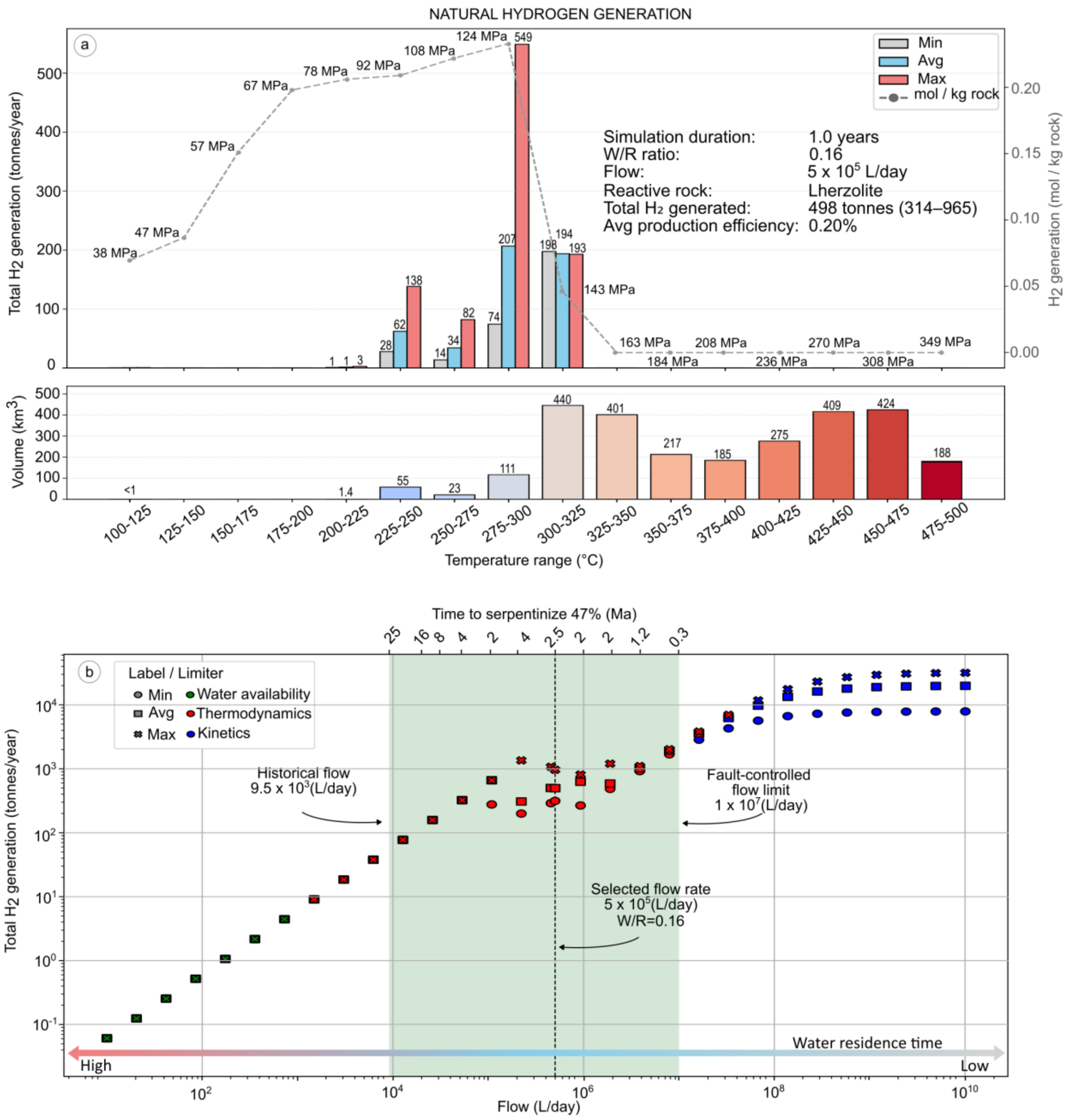

Fig. 2. Overview of $H_2(aq)$ production estimates under contrasting water availability scenarios for the Western Pyrenees. a) Results of natural hydrogen production (bars in the upper rectangle), for a flow of $5x10^5$ L/day, showing the corresponding hydrogen yield per kilogram of rock (in grey) and the volumes of partially serpentinized rocks (lower rectangle, fresh mantle not included). Numbers next to the bars indicate the H_2 production in tonnes/year and numbers next to the points represent pressure values in MPa. For visualization purposes, hydrogen production values were rounded to the nearest whole number. Production efficiency relative to unconstrained generation conditions. b) Total annual hydrogen production plotted against the daily water-flow (for a W/R=0.16). Marker shape identifies the advancing serpentinization front scenario (min, avg, max), while marker colour shows the dominant limiting factor. Green for water limitation for production below 10 tonnes/year, red for thermodynamics (e.g., saturation of H_2 in the fluid) and blue for kinetics (e.g., creation of new porosity). The shaded area highlights the plausible flow range, spanning from historically inferred values to those controlled by fault zone permeability. The dashed line at 5×10^5 L/day marks the selected intermediate scenario.

Northern California. The ~~south-~~western part of the United States of America, north of San Francisco (Fig. 3a, b), hosts extensive, shallow harzburgite-rich ophiolite complexes [32]. These include the Coast Range Ophiolite, which likely originated in a supra-subduction zone setting and was emplaced onto continental crust during Late Jurassic tectonic events [33,34]. Their present crustal position reflects not only Jurassic emplacement but also significant Cretaceous tectonic imbrication and uplift since approximately 90 Ma [35]. The presence of coarse, unfoliated bastite (orthopyroxene pseudomorphs) in some areas, however, indicates that strong tectonic overprinting or deep burial did not occur pervasively throughout the complex [36]. Great Valley forearc data indicate that Jurassic-Cretaceous syn-sedimentary extension, followed by Cenozoic folding, uplift, and strike-slip faulting linked to the shift from subduction to a transform boundary, reshaped these structures [37,38].

Currently, these complexes are at depths where temperatures and structural conditions are favourable for serpentinization [32]. Large-amplitude positive magnetic anomalies (Fig. 3c) align with the serpentinite bodies mapped in the region. In contrast, gravity highs (Fig. 3d) correspond to denser, unaltered mantle rocks [39]. Recent studies suggest that serpentinite-rich fault zones, such as those along the Bartlett Springs Fault, are associated with creep behaviour at depth, with serpentinite acting as a proxy to infer zones of aseismic slip [39]. Surface measurements showing elevated hydrogen concentrations (Fig. 3b) support serpentinization processes, suggesting active fluid migration from depth [40,41]. Geophysical inversion of gravity and magnetic data, guided by the lithospheric geometry derived from P-wave tomographic models [42], and integrated with thermal modelling (Fig. 3c, d), reveals the presence of ~12,000 km^3 of highly serpentinized rocks. Of this volume, ~4,500 km^3 fall within a temperature range capable of sustaining hydrogen generation. In addition, ~7,500 km^3 of such rocks lie outside the hydrogen production range (>325°C). Results also suggest that the degree of serpentinization of the mantle rocks in Northern California is very high, with average values approaching full serpentinization (Fig. 3f). The maximum amount of H_2 that can be produced from these rocks was estimated using a thermodynamic model [29] constructed for equilibrium conditions involving harzburgite and pure water (Fig. 4a).

The expected range of fluid input (highlighted in Fig. 4b) was derived from simulations of flow along a 250 km fault system, which coincides with the mapped extent of the ultramafic units (see Methods and Fig. 7). The lower end of this range reflects a conservative estimate of sustained flow over 90 million years, while the upper bound corresponds to the maximum

capacity of the fault system to deliver water over time. A flow rate of 2×10^6 L/day and a water-to-rock ratio (W/R) of 0.20 were selected as an intermediate reference. Across the spectrum of plausible flow scenarios, hydrogen output as dissolved H_2, is estimated to range from approximately 200 to 3,000 tonnes per year. However, under the selected intermediate flow rate, annual production falls between 283 and 367 tonnes, with a mean value of 346 tonnes per year. The main limitations to hydrogen production under this specific flow of water arise from the dynamics of the serpentinization front. As large rock volumes undergo alteration, hydrogen accumulates rapidly, leading to fluid saturation inhibiting further reactions due to thermodynamic feedbacks. Conversely, when the front advances slowly, shorter water residence times imply that reaction kinetics become the primary limiting factor.

A broader suite of simulations (Fig. 4b) explored the impact of varying water supply over ~~several~~ orders of magnitude (1 to 10^{10} L/day) and tested hydrogen generation for a fixed W/R = 0.20. At very low flows ($\leq 10^3$ L/day), the long residence time coincides with limited water availability, yielding hydrogen production below 10 tonnes/year (adopted reference threshold). In the intermediate range ($\sim 10^3$ to 10^7 L/day), residence time decreases but remains sufficient for dissolved H_2 to reach saturation, making thermodynamic constraints the dominant limiting factor. At elevated inflow rates ($>10^7$ L/day), residence time becomes very short and, although water is available in sufficient quantities to transport hydrogen, the hydrogen-generation rate is limited by reaction kinetics, capping annual output at around 5,000 tonnes (Fig. 4b).

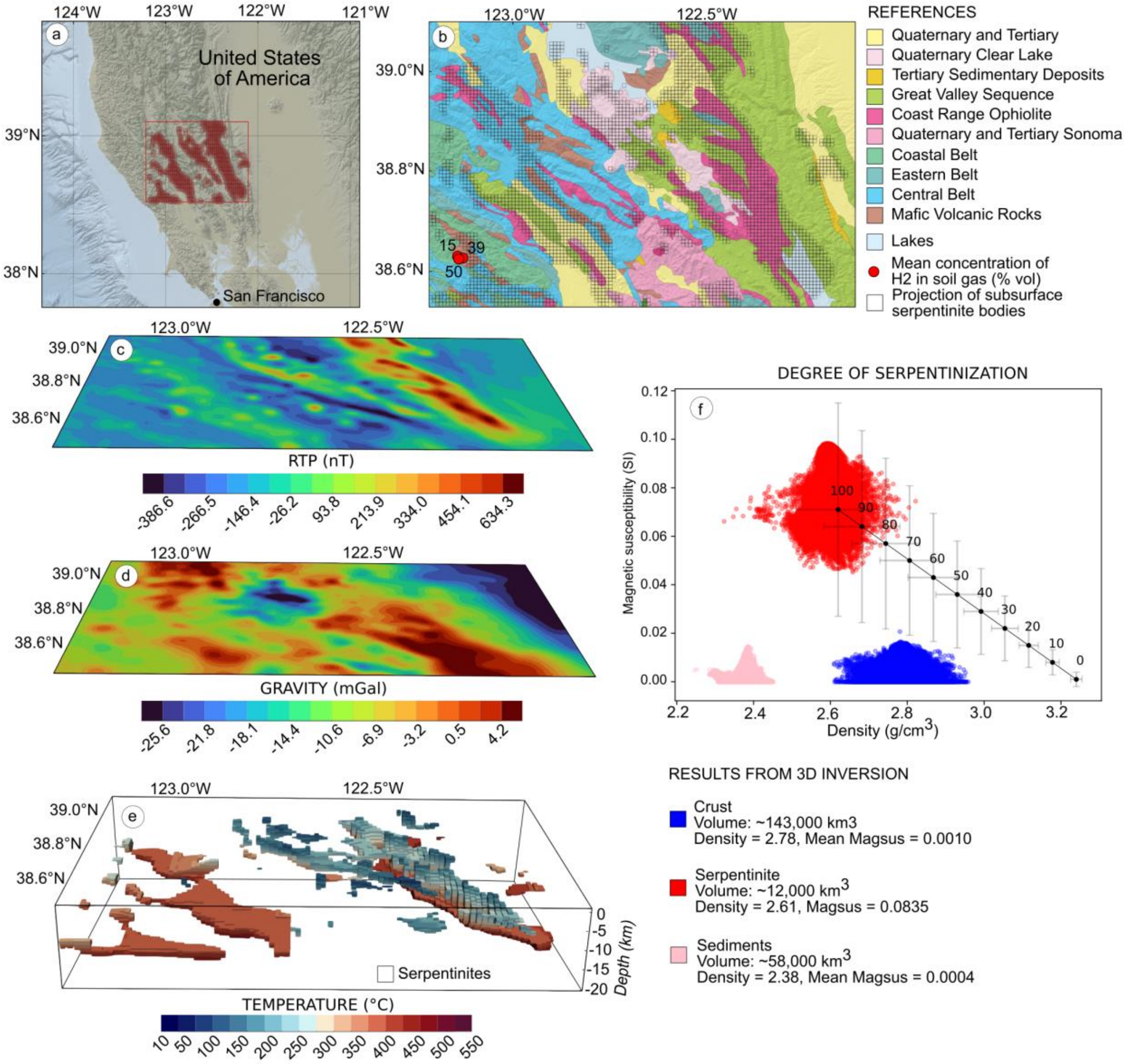

Fig. 3: Results of source rock volumes for Northern California. a) Index map of study area (red box). The Red polygons shows modeled serpentinite extent projected to the surface. b) Age of geologic units in the study area (modified from ref. 39). Polygons show modeled serpentinite blocks projected to the surface. Red circles represent H_2 measurements (%) in soil gases (modified from ref. 41). c) Reduced to the pole magnetic anomalies (RTP) for the area (modified from ref. 39). d) Residual isostatic gravity anomalies for the area (modified from ref. 39). e) 3D blocks showing temperature distribution for the serpentinites. f) Results of the Gaussian Mixture Model (GMM) indicating the degree of serpentinization based on density and magnetic susceptibility results. Values are projected to the closest serpentinization point in the line.

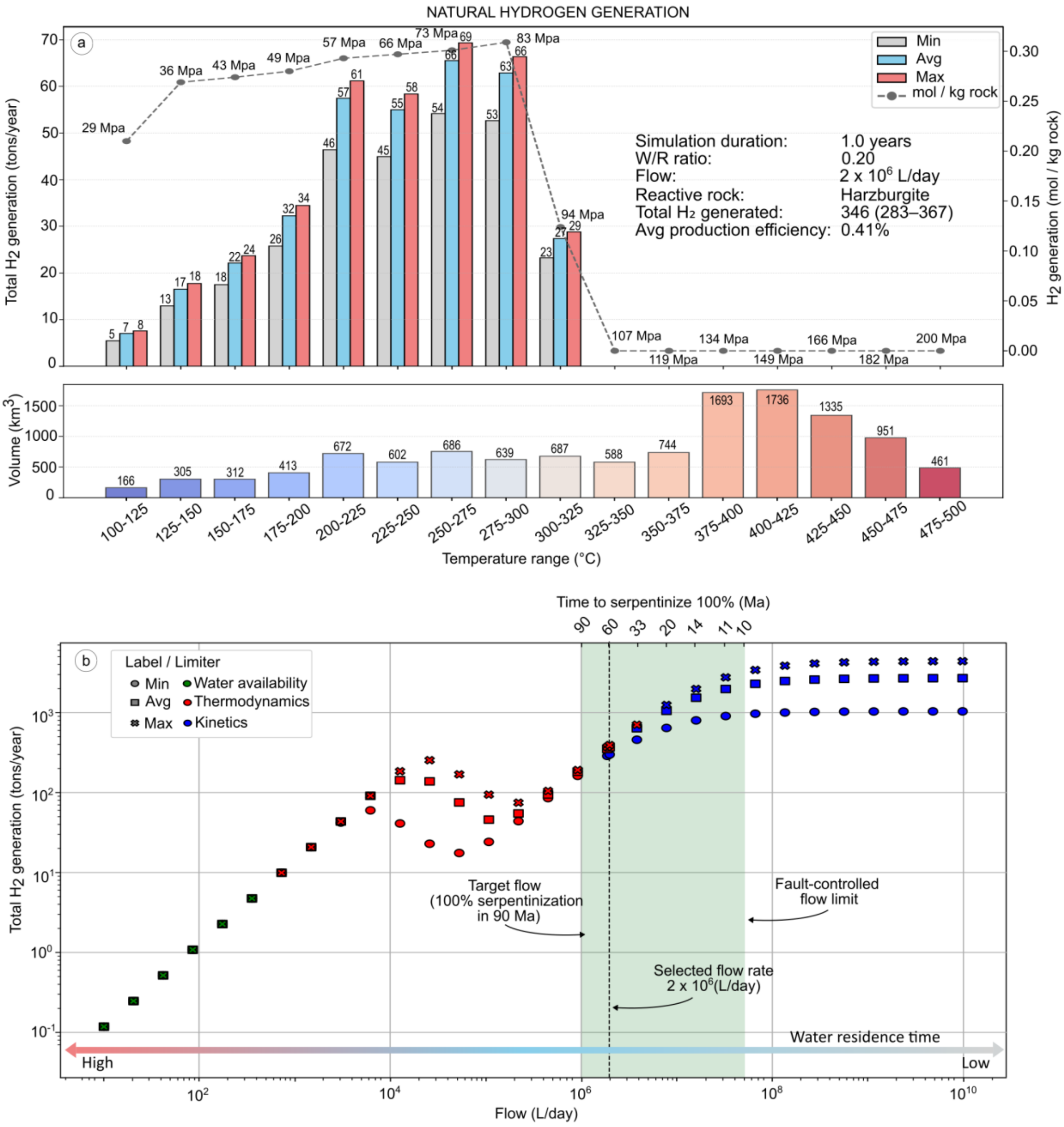

Fig. 4. Overview of H_2(aq) production estimates under contrasting water availability scenarios for Northern California. a) Results of natural hydrogen production, for a flow of 2x10^6 L/day, showing the corresponding hydrogen yield per kilogram of rock (upper rectangle) and the volumes of partially serpentinized rocks (lower rectangle). Numbers next to points represent pressure values in MPa. For visualization purposes, hydrogen production values were rounded to the nearest whole number. Production efficiency relative to unconstrained generation conditions. b) Total annual hydrogen production plotted against the daily water-flow. Marker shape identifies the advancing serpentinization front scenario (min, avg, max), while marker colour shows the dominant limiting factor. Green indicates water limitation for production below 10 tonnes/year, red for thermodynamics (e.g., saturation of H_2 in the fluid) and blue for kinetics (e.g., creation of new porosity). The shaded area highlights the plausible flow range. The dashed line at 2 × 10^6 L/day marks the selected intermediate scenario.

2. Discussion

Our three-dimensional structural models of the Western Pyrenees (Fig. 2c, d) suggest that exhumed mantle rocks extend from ~8 km to at least 20 km depth (lower limit of our model). These ultramafic rocks can be divided into two main groups, a deeper one with a low degree of serpentinization of ~10%, and a shallower one with a level of alteration of around 37 to 57%. This spatial trend coincide with previous interpretations based on gravity, magnetic, and P-wave velocity models [25,27], as well as petrophysical modelling [15]. Our results suggest that average natural hydrogen generation rate in the Western Pyrenees is ~500 tonnes/year for a reactive volume of ~630 km^3, assuming a sustained fluid flow of $5x10^5$ L/day reaching the reactive mantle peridotites. In this area, hydrogen production is mainly governed by thermodynamic factors, such as hydrogen solubility in the fluid and the limited renewal of reactive fluids. In contrast to our findings, previous studies [15,16] have estimated hydrogen production at an industrial scale ranging from 0.6 to 72 Mt per year. Of that range, only the lower bound is comparable to the output of our models when all major physical and chemical constraints on serpentinization are omitted and water availability is assumed to be virtually unlimited (see Supplementary Data 1 for detailed numerical data).

For Northern California, we estimate an average production rate of ~350 tonnes/year for a reactive volume of ~4,500 km^3 and a fluid supply of $2x10^6$ L/day. Our calculations indicate that a fault system comprising multiple connected segments over a total length of about 250 kilometres, such as the ones present in the study area can support this level of fluid flow. Although the volume of reactive rocks in this zone is greater than in the Western Pyrenees, the underlying processes are different because the mantle peridotites are almost completely serpentinized. This interpretation is in line with published seismic velocity models [42] which, when considered together with the composition of serpentinites mapped at the surface and 2D modelling of magnetic anomalies [39], point to widespread alteration of the ultramafic rocks. The reduced volume of reactive rock implies a proportionally larger amount of circulating water, which prevents rapid fluid saturation with hydrogen. As a result, hydrogen production in this area is mainly limited by reaction kinetics (e.g., the slow formation of new porosity) and only secondarily by the saturation of the fluid with the hydrogen it generates.

Our H_2 production estimates, ~0.8 tonnes H_2/km^3/year for the Pyrenees and ~0.07 tonnes H_2/km^3/year for Northern California, are in the range of natural hydrogen flow rates based on H_2 measurements in other crustal geological settings. For instance, 200 tonnes per year have

been reported in the Bulqizë ophiolite in Albania for an approximate volume of ultramafic rocks of 1110 km^3 [13], corresponding to ~0.2 tonnes H_2/km^3/year. Similarly, 808 tonnes per year have been observed in the Zambales ophiolite in Philippines [14] for a volume of ultramafic rocks of approximately 550 km^3 [43], corresponding to ~1.4 tonnes H_2/km^3/year. However, it should be noted that these latter values likely represent emissions from subsurface gas reservoirs rather than actual production rates. However, if one considers that only the excess hydrogen escapes while the rest accumulates, these flow rates may approximate the true production levels once the reservoir pressure has equilibrated.

In both cases, hydrogen output remains well below the unconstrained theoretical maximum (see Supplementary Data 1 and Supplementary Data 2 for detailed numerical data), primarily due to kinetic or thermodynamic constraints, depending on water flow rates. Nonetheless, even under these constraints, the modelled serpentinization rates would lead to complete hydration of the ultramafic volume well before the time elapsed since the exhumation of the mantle bodies. However, the present scenario likely does not apply to the early stages of serpentinization, when large volumes of fresh rock were exposed to water inputs probably comparable to present-day flow rates. In such conditions, the initial water-to-rock ratio would have been extremely low, limiting reaction progress and resulting in very slow hydrogen production. As serpentinization progressed and the reactive rock fraction decreased, the water-to-rock ratio would have gradually increased, allowing for more extensive fluid-rock interaction and a progressive acceleration of hydrogen generation. Furthermore, our results show that diffusive transport through serpentinites alone is insufficient to supply the fluid volumes required for sustained serpentinization (Figs. 5 and 6). This finding reinforces previous laboratory evidence indicating that fracture-controlled flow is the dominant mechanism for fluid delivery in ultramafic settings [44].

The reported hydrogen production rates are relatively modest (<500 tonnes per year) and likely represent an upper bound under geologically realistic conditions. At such production rates, the hydrogen volumes would be sufficient to supply only a few thousand households, suggesting that continuous natural hydrogen generation may be considered a renewable resource only at localized or small scales. In addition, hydrogen is predominantly generated and transported in the aqueous phase, remaining dissolved throughout the production zone and during its migration upward toward shallower depths. Only limited amounts of free gas may separate from solution at the generation zone, but the volumes involved are expected to be minimal.

However, if favourable structural conditions exist, such as low-permeability seals and effective traps, then local accumulations may occur, potentially increasing the extractable fraction. Importantly, increasing fluid flow would not necessarily translate into higher recoverable volumes, because hydrogen would become increasingly diluted in the fluid phase, further reducing the likelihood of forming detectable natural accumulations. For this reason, future work should integrate hydrogen generation models with reactive transport and migration simulations, and assess the potential for phase separation and retention to better constrain the viability of subsurface hydrogen accumulations.

3. Methods

Subsurface Modelling and Petrophysical Constraints. Initial three-dimensional models of the primary subsurface rock units (e.g., mantle rocks, serpentinized rocks, basement rocks) are constructed based on 2D sections. These initial models are not intended to represent detailed geological structures and serve only as initial guides to help the inversion converge toward geologically plausible solutions. Petrophysical data are parametrized from seismic velocity models (see Supplementary Table 1 and Supplementary Table 2 for more information). A Petrophysically Guided Inversion (PGI) approach [45,46] is used to invert observed gravity and magnetic anomalies for density and magnetic susceptibility, constrained by the initial petrophysical relationships. Cells consistent with serpentinized rocks are identified based on their petrophysical characteristics (Figs. 1e and 3f), and the volumes of these units are determined.

Thermal Modelling. Subsurface temperature distributions (Figs. 5a and 6a) are calculated by solving the steady-state heat conduction equation using the finite volume method as implemented in the FiPy package [47]. At each depth interval, thermal properties are simulated using piecewise-linear functions representing the natural variability of the thermal conductivity with depth. A fixed temperature at the surface (i.e., Dirichlet boundary condition), and a background heat flow at the base (i.e., Neumann boundary condition) are imposed. The identified volumes of partially serpentinized rocks from the geophysical inversion are compared with the temperature distribution. In principle, ultramafic rocks in the 100 °C to 500 °C range are considered capable of generating hydrogen, although this range is subsequently narrowed based on the constraints of the thermodynamic databases.

H_2 Production Database. Thermodynamic equilibrium calculations were performed for two generic ultramafic rock compositions with pure water: lherzolite (Al_2O_3 4.46, CaO 2.65, FeO 7.86, MgO 40.13, SiO_2 44.75 wt%) and harzburgite (Al_2O_3 3.13, CaO 1.25, FeO 8.69, MgO 43.53, SiO_2 43.33 wt%). Maximum H_2 production reaches around 250 mmol/kg rock for lherzolite and 300 mmol/kg rock for harzburgite (Figs. 5b; 6b) while maximum absorbed water into the rock for these productions reaches around 0.75 mol H_2O/kg rock (Figs. 5c and 6c). Thermodynamic calculations are conducted using the Gibbs free energy minimization software Theriak-Domino [48], applying the publicly available JUN92d.bs database and a version of the TC-DS633 dataset, both derived from the Holland & Powell [49,50] thermodynamic framework [29]. Solid solution models for Fe(III)-bearing serpentines [51] were not included in these versions. As a result, hydrogen generation is attributed only to magnetite formation.

Serpentinization Framework. A correction was applied to account for the loss in hydrogen generation due to the progressive volume replacement of peridotite by serpentinite (Figs. 5d and 6d), assuming serpentinization progresses inward from the periphery of each block (see *Fracture Network, Serpentinization Front Velocities and H_2 Generation Scenarios* for details) and accounting for unaltered olivine cores. As a result, the hydrogen generation potential of each block is reduced compared to an entirely unaltered peridotite volume, since only the remaining unaltered fraction is available for ongoing serpentinization reactions. The serpentinization degree is estimated by comparing the average density and magnetic susceptibility weighted by the proportions of altered and unaltered material to reference datasets [7–9,52,53]. Even at advanced degrees of serpentinization, a small fraction (1 - 4%) of unaltered primary minerals (typically olivine or pyroxene) is expected to remain [54,55]. To conservatively account for isolated or kinetically inhibited volumes resistant to complete alteration at the core, a residual value of 2% is adopted in this study.

Fracture Network, Serpentinization Front Velocities and H2 Generation Scenarios. Reactive rock blocks with sub-meter to meter-scale dimensions are defined based on fracture spacing observed in outcrops [56–60]. To estimate the progression velocities of the serpentinization front across temperature ranges (Figs. 5e and 6e), we adopt a scaling approach based on hydrogen production rates. A reference temperature-pressure condition with a known front velocity (e.g., reference 61) is first selected. The serpentinization front propagation velocity at temperature T, pressure P and kinetic scenario kv, is then estimated by scaling the reference velocity according to the relative hydrogen production rate

$$v_f(P,T,kv) = v_{ref} \cdot \frac{R_{H2}(T,P)}{R_{H2}^{ref}} \cdot f(kv) \quad (1)$$

where v_{ref} is a reference velocity, $R_{H2}(T,P)$ is the hydrogen production rate at those conditions, R_{H2}^{ref} is the rate at reference conditions and $f(kv)$ is a scaling factor that captures the uncertainty associated with kinetic conditions (minimum, average, and maximum).

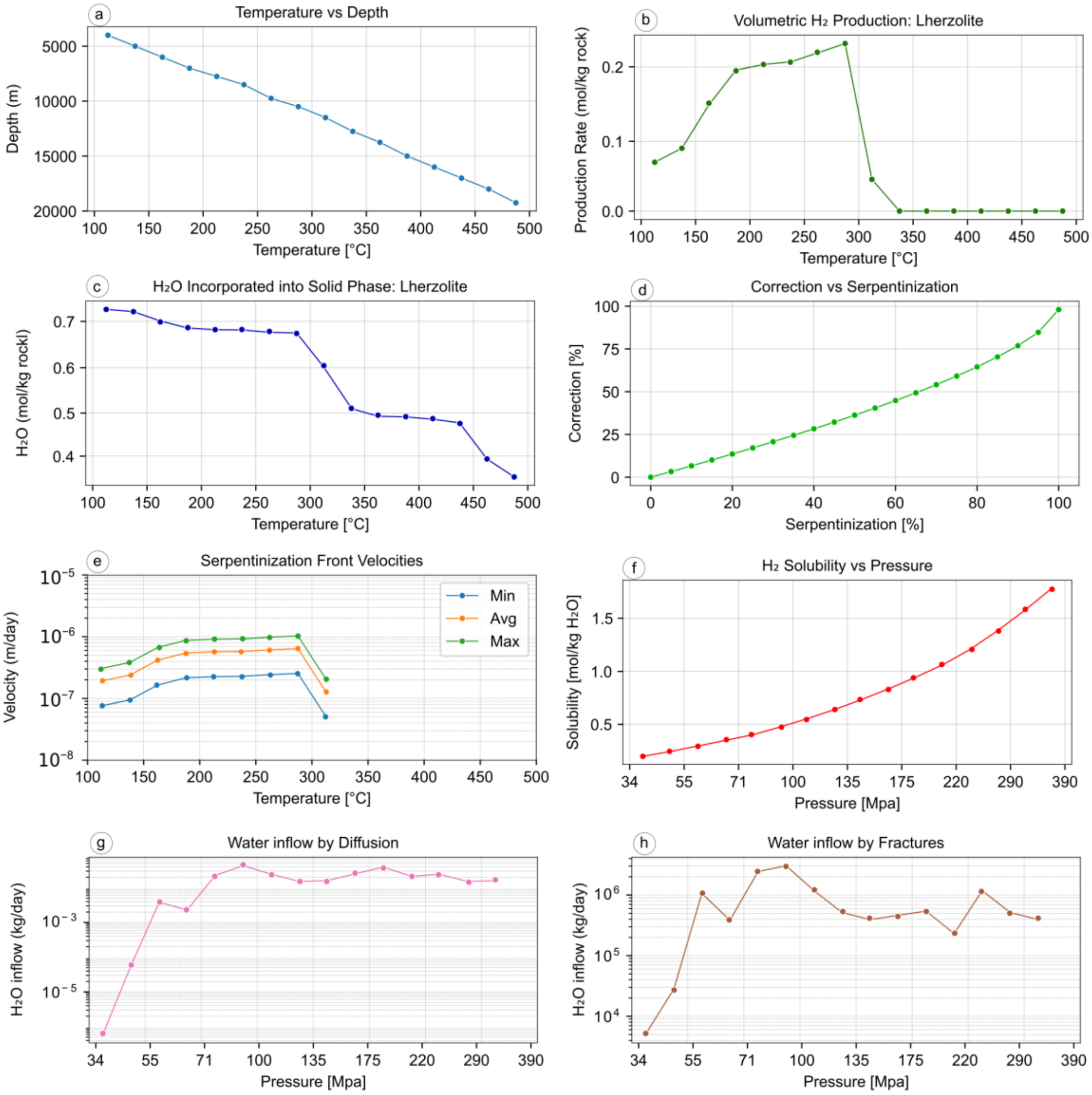

Fig. 5. Thermodynamic and kinetic parameters of serpentinization modelled for the studied P-T conditions in the Western Pyrenees (see Supplementary Data 1 for detailed numerical data). a) Modelled temperature profile as a function of depth. b) Volumetric hydrogen production rates (mol H_2/kg rock) estimated for each temperature interval based on thermodynamic modelling for lherzolite. c) Amount of water incorporated into the solid phase during serpentinization (mol H2O/kg rock). d) Empirical correction factors as a function of serpentinization degree. e) Estimated serpentinization front velocities showing minimum, average, and maximum advance rates. Non-existent values reflect conditions where serpentinization is not active. f) Solubility of molecular hydrogen in water as a function of pressure, calculated from empirical relations [62]. g) Modelled water inflow rates by molecular diffusion across the serpentinized shell as a function of pressure. h) Estimated water inflow rates across the serpentinized shell through fracture-controlled advection, based on fracture permeability models parameterized for the studied lithologies and pressure gradients.

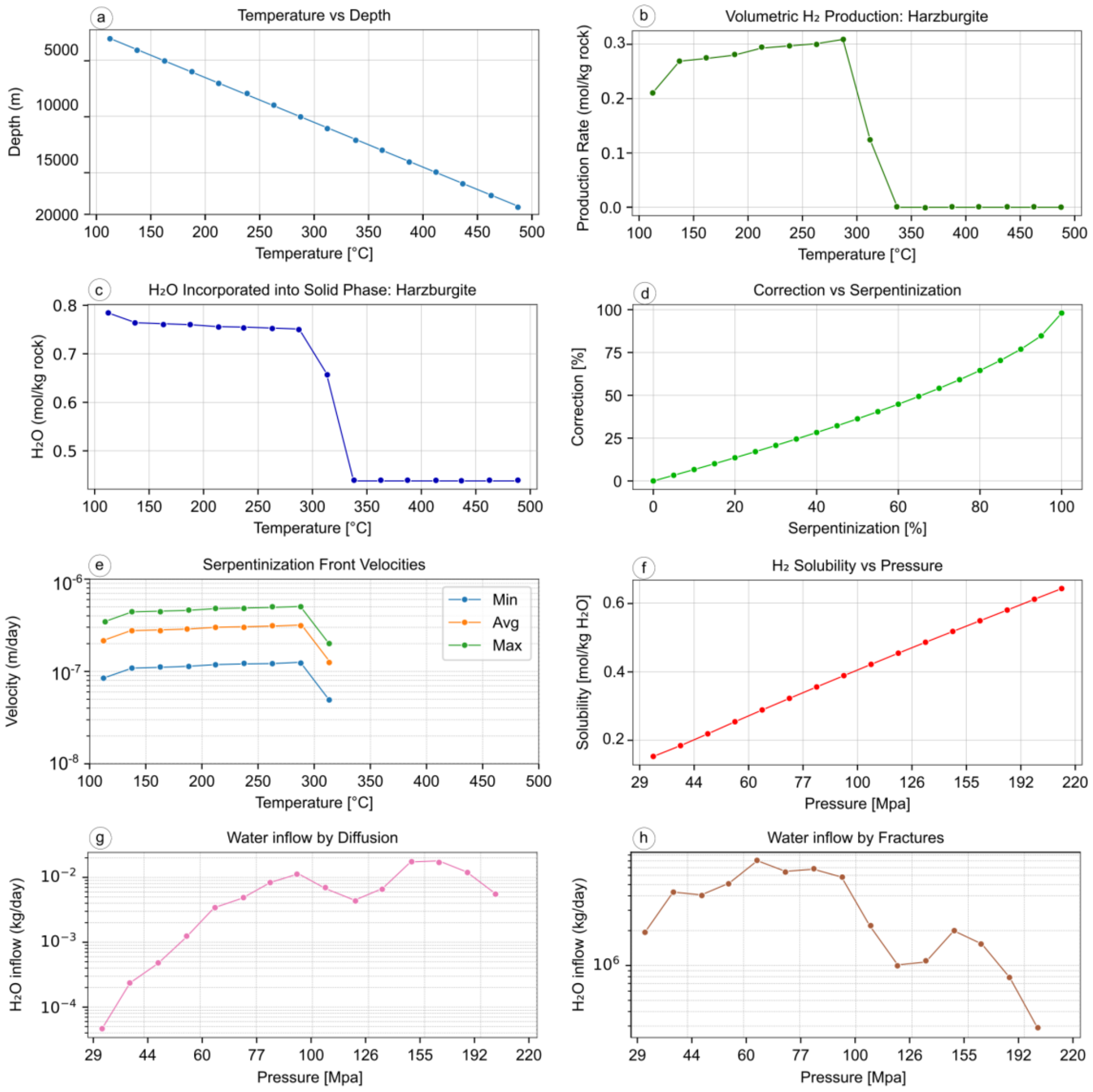

Fig. 6. Thermodynamic and kinetic parameters of serpentinization modelled for the studied P-T conditions in the Northern California (see Supplementary Data 2 for detailed numerical data). a) Modelled temperature profile as a function of depth. b) Volumetric hydrogen production rates (mol H_2/kg rock) estimated for each temperature interval based on thermodynamic modelling for lherzolite. c) Amount of water incorporated into the solid phase during serpentinization (mol H2O/kg rock). d) Empirical correction factors as a function of serpentinization degree. e) Estimated serpentinization front velocities showing minimum, average, and maximum advance rates. Non-existent values reflect conditions where serpentinization is not active. f) Solubility of molecular hydrogen in water as a function of pressure, calculated from empirical relations [62]. g) Modelled water inflow rates by molecular diffusion across the serpentinized shell as a function of pressure. h) Estimated water inflow rates across the serpentinized shell through fracture-controlled advection, based on fracture permeability models parameterized for the studied lithologies and pressure gradients.

Deep Water Flow Capacity Estimation. A Monte Carlo approach was used to test the deep-water flow potential under different fracture network configurations. Although the analysed faults have the capacity to transport the volumes of water used in our calculations, the potential source of this water has not been established. Flow is calculated as a function of permeability, fracture area, depth, pressure gradient, and fluid viscosity using Darcy's law. Fracture network geometries were generated by sampling key parameters across physically plausible ranges. Maps of projected serpentinites were used to extract fault segments, while damage zone thickness was randomly sampled within the 1-1,000 m range to reflect the observed variability in fracture network widths. In these zones, 5-30% of the fault length was assumed hydraulically active, while fracture density decreases inversely with depth. The total fracture wall area available for flow was computed as a function of fracture count, fault length, and vertical extent. Effective permeability was sampled based on an empirical depth-dependent trend [63]. A hydraulic connection fraction of 10-25% was applied to simulate partial connectivity among fractures. Pressure differentials driving flow were sampled between 60 and 140 MPa, representing lithostatic-hydrostatic contrasts at depths ranging from 1 to 12 km. Water viscosity was varied between 1×10^{-3} and 1×10^{-5} Pa·s, reflecting temperatures from near-surface to ~600 °C. For each realization, permeability and all other variables were combined to estimate resulting flow rates. Success was defined as achieving or exceeding a pre-defined target flux in L/day. The simulations quantified success rates and identified flow rates sustainable in at least 70% of cases (Fig. 7).

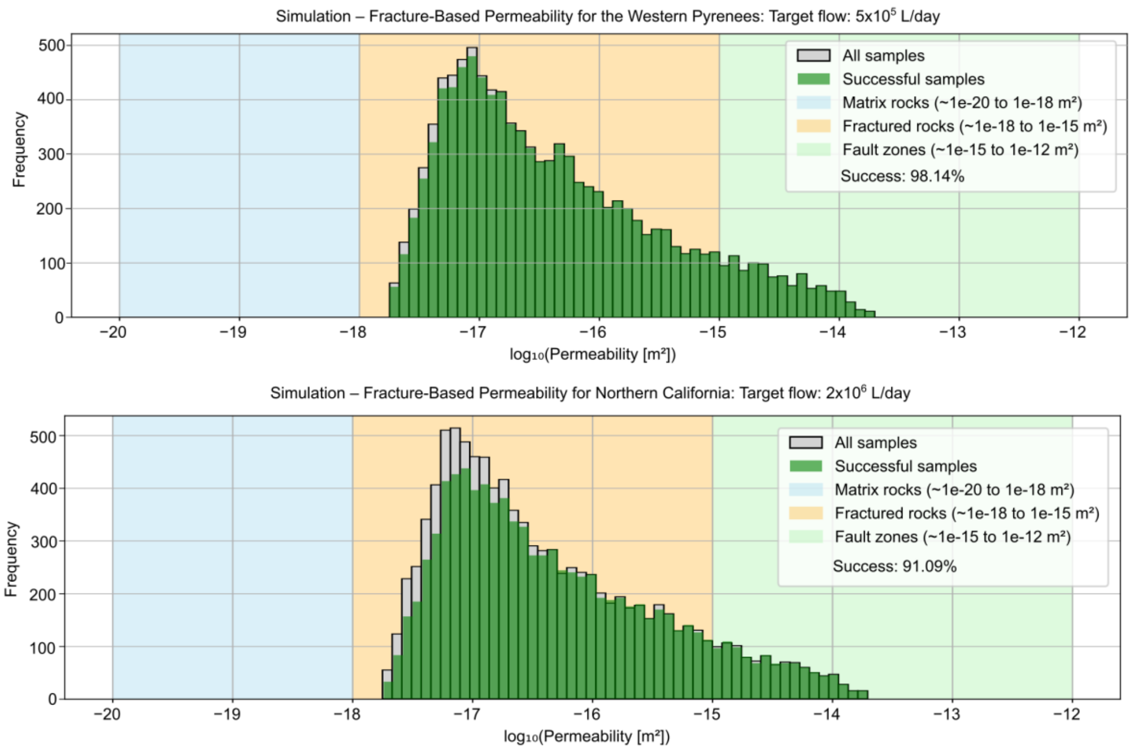

Fig. 7. Permeability distributions and hydraulic success for water delivery into serpentinizing mantle rocks. Top: Western Pyrenees simulations; Bottom: Northern California simulations. The histograms show the full ensemble of sampled fracture permeabilities (gray) and the subset of realizations that meet or exceed the target deep water flux (green). Shaded background regions indicate approximate permeability ranges for matrix rocks (blue: ~10^{-20}-10^{-18} m²), fractured rocks (orange: ~10^{-18}-10^{-15} m²), and fault zones (green: ~10^{-15}-10^{-12} m²). Simulations consider stochastic variability in fracture network geometry, depth, fluid pressure, viscosity, and hydraulic connectivity.

Hydrogen Production Estimation. Hydrogen generation is first estimated under unconstrained conditions, assuming no kinetic or thermodynamic limitations (see Supplementary 1 for a more detailed mathematical explanation). For each temperature and pressure condition the theoretical hydrogen generation is calculated as

$$n_{H2}^{unconstrained}(T, P, k_v) = \rho_{rock} \cdot R_{H2}(T, P) \cdot V_{front}(P, T, k_v) \quad (2)$$

where ρ_{rock} is the rock density and R_{H2} is the hydrogen generation rate derived from the thermodynamic modelling and V_{front} is the advancing reactive volume under kinetic scenario k_v. These values represent the theoretical upper limit of hydrogen production, determined solely by the intrinsic properties of the rock and the extent of serpentinization.

In practice, production is constrained by water availability, saturation, and evolving porosity at the reaction front. As serpentinization progresses, porosity increases and promotes water inflow, but saturation effects progressively reduce hydrogen release. A fixed daily water flow is imposed and distributed across temperature intervals in proportion to their reactive volume

(Fig. 8a), ensuring preferential allocation to active zones (i.e., zones with higher fracture density and permeability). Within each interval, water is further partitioned between diffusive inflow and fracture-controlled flow (Fig. 8b to d), governed by distinct transport laws.

Hydrogen solubility in water $S_{max}(P)$ is estimated using an empirical relation [62]. The maximum amount of hydrogen that the available water can retain under in-situ pressure conditions is

$$n_{H2}^{max}(\mathrm{T,P},k_v) = S_{max}(P) \cdot M_{H2O}(T,P,k_v) \tag{3}$$

where M_{H2O} includes both water filling newly formed porosity and inflow from diffusion and fractures. The model tracks the cumulative amount of hydrogen already dissolved in the water of each cell $n_{H2}^{dissolved}$. This is used to update the saturation ratio, which limits further production

$$S_{H2}^{eq}(\mathrm{T,P},k_v) = \frac{n_{H2}^{dissolved}(\mathrm{T,P},k_v)}{n_{H2}^{max}(\mathrm{T,P},k_v)} \tag{4}$$

As saturation increases, less hydrogen can be retained in the aqueous phase. The effective hydrogen production is thus scaled by the fraction of unsaturated capacity

$$n_{H2}^{cell}(\mathrm{T,P},k_v) = n_{H2}^{unconstrained}(T,P,k_v) \cdot \left[1 - S_{H2}^{eq}(\mathrm{T,P},k_v)\right] \tag{5}$$

The total production is finally obtained by summing contributions from all reactive cells over the simulation period (typically one year).

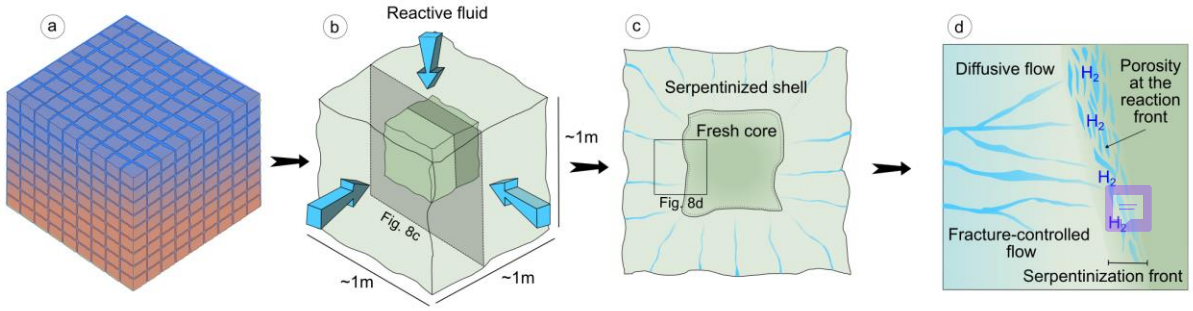

Fig. 8. Conceptual representation of the serpentinization front architecture and associated fluid transport mechanisms. (a) The model domain is discretized into cubic elements for integration with the coupled thermodynamic and kinetic framework. (b) The reactive fluid enters each ~1 m block through external boundaries (fracture system), simulating infiltration across fracture-controlled pathways and diffusive gradients. (c) Progressive alteration results in the formation of a serpentinized shell enclosing a progressively shrinking fresh ultramafic core. (d) At the serpentinization front, hydrogen (H_2) is generated as unaltered minerals react with the infiltrating fluid. Access of the reactive fluid to the alteration front is governed by two transport processes: (i) diffusive flow across the low-permeability serpentinized shell, parameterized as a function of temperature and pressure, and (ii) fracture-controlled flow, controlled by fracture network permeability and pressure gradients. The evolving porosity at the reaction front regulates both fluid accessibility and reaction progression.

Data availability

Note: All data supporting the findings of this study, as well as the PoNHy (Potential for Natural Hydrogen) source code, will be made openly available on GitHub upon publication. For peer review, we have included all necessary materials to ensure full reproducibility of the results as part of the submission.

Acknowledgements

This work was developed within the framework of the HyAfrica project, part of the Long-term Europe-Africa Partnership on Renewable Energy (LEAP-RE) programme. LEAP-RE has received funding from the European Union’s Horizon 2020 Research and Innovation Programme under Grant Agreement No. 963530. German partners of HyAfrica received funding from the German Ministry for Education and Research (BMBF), LIAG under grant 03SF0680B. We would like to thank Tom McCollom for his generous discussions regarding hydrogen production rates in open systems. We acknowledge the SimPEG project for providing open-source geophysical inversion libraries, which greatly facilitated the development of our modeling framework.

Author Contributions

R.C. designed the methodology, developed and implemented the full modelling framework, wrote the source code, and led the writing of the manuscript. M.S. contributed to the development of the code, assisted with the geophysical inversion and writing of the manuscript. C.O.-H. provided key input on the methodology and serpentinization processes, contributed to the conceptual framework and writing of the manuscript. G.G. contributed to the tectonic interpretation and writing of the manuscript. N.S. and S.C. provided geological and geophysical data for the Western Pyrenees and contributed to the regional interpretation and scientific discussions. V.L. contributed geological data and regional interpretation for the Northern California case study, as well as writing of the manuscript and scientific discussions. J.G.-P. provided guidance on the thermodynamic modelling, contributed to the conceptual development of the study and scientific discussions. G. Gabriel supervised the project, contributed to discussions throughout and writing of the manuscript. All authors reviewed the manuscript and contributed to the interpretation of the results.

Corresponding author

Correspondence to Rodolfo Christiansen - Rodolfo.Christiansen@liag-institut.de

Competing interests

The authors declare no competing interests.

References

1. Mayhew, L. E., Ellison, E. T., McCollom, T. M., Trainor, T. P. & Templeton, A. S. Hydrogen generation from low-temperature water–rock reactions. *Nature Geosci* **6**, 478–484 (2013).
2. McCollom, T. M. *et al.* Temperature trends for reaction rates, hydrogen generation, and partitioning of iron during experimental serpentinization of olivine. *Geochimica et Cosmochimica Acta* **181**, 175–200 (2016).
3. Deville, E. & Prinzhofer, A. The origin of N2-H2-CH4-rich natural gas seepages in ophiolitic context: A major and noble gases study of fluid seepages in New Caledonia. *Chemical Geology* **440**, 139–147 (2016).
4. Lamadrid, H. M. *et al.* Effect of water activity on rates of serpentinization of olivine. *Nat Commun* **8**, 16107 (2017).
5. Barbier, S. *et al.* A Review of H2, CH4, and Hydrocarbon Formation in Experimental Serpentinization Using Network Analysis. *Front. Earth Sci.* **8**, 209 (2020).
6. Macdonald, A. H. & Fyfe, W. S. Rate of serpentinization in seafloor environments. *Tectonophysics* **116 (1–2)**, 123–135 (1985).
7. Miller, D. J. & Christensen, N. I. *Seismic Velocities of Lower Crustal and Upper Mantle Rocks from the Slow-Spreading Mid-Atlantic Ridge, South of the Kane Transform Zone (MARK). Proceedings of the Ocean Drilling Program, 153 Scientific Results*. vol. 153 (Ocean Drilling Program, 1997).
8. Bonnemains, D. *et al.* Magnetic signatures of serpentinization at ophiolite complexes. *Geochem Geophys Geosyst* **17**, 2969–2986 (2016).

9. Chibati, N., Géraud, Y. & Essa, K. S. Petrophysical characterization and thermal conductivity prediction of serpentinized peridotites. *Geophysical Journal International* **231**, 1786–1805 (2022).

10. Blay-Roger, R. *et al.* Natural hydrogen in the energy transition: Fundamentals, promise, and enigmas. *Renewable and Sustainable Energy Reviews* **189**, 113888 (2024).

11. Ballentine, C. J. *et al.* Natural hydrogen resource accumulation in the continental crust. *Nat Rev Earth Environ* **6**, 342–356 (2025).

12. Brunet, F. & Malvoisin, B. Can natural H2 be considered renewable? The reference case of a deep aquifer in an intracratonic sedimentary basin. *Advances in Geochemistry and Cosmochemistry* **1**, 738 (2025).

13. Truche, L. *et al.* A deep reservoir for hydrogen drives intense degassing in the Bulqizë ophiolite. *Science* **383**, 618–621 (2024).

14. Aquino, K. A. *et al.* High hydrogen outgassing from an ophiolite-hosted seep in Zambales, Philippines. *International Journal of Hydrogen Energy* **105**, 360–366 (2025).

15. Pajang, S., Mouthereau, F., Robert, A., Kumar, A. & Callot, J. A Petro-Physical Model for Serpentinized Mantle and Origin of Natural Hydrogen in the Pyrenees. *Geochem Geophys Geosyst* **26**, e2024GC011804 (2025).

16. Zwaan, F. *et al.* Rift-inversion orogens are potential hot spots for natural H2 generation. *Science Advances* **11(8)**, (2025).

17. Le Roux, V. *et al.* The Lherz spinel lherzolite: Refertilized rather than pristine mantle. *Earth and Planetary Science Letters* **259**, 599–612 (2007).

18. Tichadou, C. *et al.* Mineralogical and geochemical study of serpentinized peridotites from the North-Western Pyrenees: New insights on serpentinization along magma-poor continental passive margins. *Lithos* **406–407**, 106521 (2021).

19. Clerc, C., Lagabrielle, Y., Neumaier, M., Reynaud, J.-Y. & De Saint Blanquat, M. Exhumation of subcontinental mantle rocks: evidence from ultramafic-bearing clastic deposits nearby the Lherz peridotite body, French Pyrenees. *Bulletin de la Société Géologique de France* **183**, 443–459 (2012).
20. Mouthereau, F. *et al.* Placing limits to shortening evolution in the Pyrenees: Role of margin architecture and implications for the Iberia/Europe convergence: Plate convergence in the Pyrenees. *Tectonics* **33**, 2283–2314 (2014).
21. Chevrot, S. *et al.* The non-cylindrical crustal architecture of the Pyrenees. *Sci Rep* **8**, 9591 (2018).
22. Lefeuvre, N. *et al.* Native H_2 Exploration in the Western Pyrenean Foothills. *Geochem Geophys Geosyst* **22**, e2021GC009917 (2021).
23. Saspiturry, N. *et al.* Closure of a hyperextended system in an orogenic lithospheric pop-up, Western Pyrenees: The role of mantle buttressing and rift structural inheritance. *Terra Nova* **32**, 253–260 (2020).
24. Wang, Y. *et al.* The deep roots of the western Pyrenees revealed by full waveform inversion of teleseismic P waves. *Geology* **44**, 475–478 (2016).
25. Saspiturry, N., Allanic, C. & Peyrefitte, A. Serpentinization and Magmatic Distribution in a Hyperextended Rift Suture: Implication for Natural Hydrogen Exploration (Mauléon Basin, Pyrenees). *Tectonics* **43**, e2024TC008385 (2024).
26. Lehujeur, M. *et al.* Three-dimensional shear velocity structure of the Mauléon and Arzacq Basins (Western Pyrenees). *BSGF - Earth Sci. Bull.* **192**, 47 (2021).
27. Lescoutre, R. *et al.* The fingerprint of structural inheritances in the W-Pyrenees revealed by machine learning detection of seismicity. *Tectonophysics* **903**, 230685 (2025).
28. Lefeuvre, N. *et al.* Natural hydrogen migration along thrust faults in foothill basins: The North Pyrenean Frontal Thrust case study. *Applied Geochemistry* **145**, 105396 (2022).

29. García-Pintado, J. & Pérez-Gussinyé, M. Shallow peridotites at magma-poor rifted margins: occurrences, serpentinization, and H2 generation. Natural Hydrogen Systems: Properties, Ocurrences, Generation Mechanisms, Exploration, Storage and Transportation. *(R. Rezaee & B. Evans, Eds) De Gruyter Academic Publishing.* (2025) doi:https://doi.org/10.1515/9783111437040-005.
30. Barnolas, A. *et al.* Mapa geológico de los Pirineos a escala 1: 400 000. (2008).
31. Le Maire, P. *et al.* New Magnetic compilation and interpretation of the Bay of Biscay and surrounding continental shelves. *BSGF - Earth Sci. Bull.* **192**, 58 (2021).
32. Shervais, J. W. *et al.* Multi-Stage Origin of the Coast Range Ophiolite, California: Implications for the Life Cycle of Supra-Subduction Zone Ophiolites. *International Geology Review* **46**, 289–315 (2004).
33. Godfrey, N. J., Beaudoin, B. C. & Klemperer, S. L. Ophiolitic basement to the Great Valley forearc basin, California, from seismic and gravity data: Implications for crustal growth at the North American continental margin. *Geological Society of America Bulletin* **109**, 1536–1562 (1997).
34. Arkula, C. *et al.* The forearc ophiolites of California formed during trench-parallel spreading: Kinematic reconstruction of the western USA Cordillera since the Jurassic. *Earth-Science Reviews* **237**, 104275 (2023).
35. Frei, L. S. & Blake, M. C. Remagnetization of the coast range ophiolite and lower part of the Great Valley Sequence in northern California and southwest Oregon. *J. Geophys. Res.* **92**, 3487–3499 (1987).
36. Smith, C. & Griggs, A. *Chromite Deposits near San Luis Obispo, San Luis Obispo County, California*. https://pubs.usgs.gov/publication/b945B (1944) doi:10.3133/b945B.
37. Constenius, K. N., Johnson, R. A., Dickinson, W. R. & Williams, T. A. Tectonic evolution of the Jurassic–Cretaceous Great Valley forearc, California: Implications for

the Franciscan thrust-wedge hypothesis. *Geological Society of America Bulletin* **112**, 1703–1723 (2000).

38. McLaughlin, R. J., Moring, B. C., Hitchcock, C. S. & Valin, Z. C. *Framework Geologic Map and Structure Sections along the Bartlett Springs Fault Zone and Adjacent Area from Round Valley to Wilbur Springs, Northern Coast Ranges, California*. 60 https://doi.org/10.3133/sim3395 (2018) doi:10.3133/sim3395.
39. Langenheim, V. E., McLaughlin, R. J. & Melosh, B. L. Integrated geologic and geophysical modeling across the Bartlett Springs fault zone, northern California (USA): Implications for fault creep and regional structure. *Geosphere* **20**, 129–151 (2024).
40. Gelman, S. E., Hearon, J. S. & Ellis, G. S. Prospectivity Mapping for Geologic Hydrogen. *US Geological Survey* **1900**, (2025).
41. Morrill, P. L. *et al.* Geochemistry and geobiology of a present-day serpentinization site in California: The Cedars. *Geochimica et Cosmochimica Acta* **109**, 222–240 (2013).
42. Furlong, K. P., Villaseñor, A., Benz, H. M. & McKenzie, K. A. Formation and Evolution of the Pacific-North American (San Andreas) Plate Boundary: Constraints From the Crustal Architecture of Northern California. *Tectonics* **43**, e2023TC007963 (2024).
43. Abrajano, T. A., Pasteris, J. D. & Bacuta, G. C. Zambales ophiolite, Philippines I. Geology and petrology of the critical zone of the Acoje massif. *Tectonophysics* **168**, 65–100 (1989).
44. Farough, A., Moore, D. E., Lockner, D. A. & Lowell, R. P. Evolution of fracture permeability of ultramafic rocks undergoing serpentinization at hydrothermal conditions: An experimental study. *Geochem Geophys Geosyst* **17**, 44–55 (2016).
45. Astic, T., Heagy, L. J. & Oldenburg, D. W. Petrophysically and geologically guided multi-physics inversion using a dynamic Gaussian mixture model. *Geophysical Journal International* **224**, 40–68 (2020).

46. Astic, T. & Oldenburg, D. W. A framework for petrophysically and geologically guided geophysical inversion using a dynamic Gaussian mixture model prior. *Geophysical Journal International* **219**, 1989–2012 (2019).
47. Guyer, J. E., Wheeler, D. & Warren, J. A. FiPy: Partial Differential Equations with Python. *Comput. Sci. Eng.* **11**, 6–15 (2009).
48. De Capitani, C. & Petrakakis, K. The computation of equilibrium assemblage diagrams with Theriak/Domino software. *American Mineralogist* **95**, 1006–1016 (2010).
49. Holland, T. J. B. & Powell, R. An improved and extended internally consistent thermodynamic dataset for phases of petrological interest, involving a new equation of state for solids: THERMODYNAMIC DATASET FOR PHASES OF PETROLOGICAL INTEREST. *Journal of Metamorphic Geology* **29**, 333–383 (2011).
50. Holland, T. J. B. & Powell, R. An internally consistent thermodynamic data set for phases of petrological interest. *Journal Metamorphic Geology* **16**, 309–343 (1998).
51. Eberhard, L., Frost, D. J., McCammon, C. A., Dolejš, D. & Connolly, J. A. D. Experimental Constraints on the Ferric Fe Content and Oxygen Fugacity in Subducted Serpentinites. *Journal of Petrology* **64**, egad069 (2023).
52. Chen, H. *et al.* Induced Polarization and Magnetic Responses of Serpentinized Ultramafic Rocks From Mid-Ocean Ridges. *JGR Solid Earth* **126**, e2021JB022915 (2021).
53. Cutts, J. A. *et al.* Deducing Mineralogy of Serpentinized and Carbonated Ultramafic Rocks Using Physical Properties With Implications for Carbon Sequestration and Subduction Zone Dynamics. *Geochem Geophys Geosyst* **22**, e2021GC009989 (2021).
54. Janecky, D. R. Serpentinization Reaction Pathways: Implications for Modeling Approach. *Proceedings of the Workshop on Geochemical Modeling* 80 (1986).
55. Huang, R., Sun, W., Ding, X., Zhao, Y. & Song, M. Effect of pressure on the kinetics of peridotite serpentinization. *Phys Chem Minerals* **47**, 33 (2020).

56. Coleman, R. G. Petrologic and Geophysical Nature of Serpentinites. *Geol Soc America Bull* **82**, 897 (1971).
57. O'Hanley, D. S. Solution to the volume problem in serpentinization. *Geol* **20**, 705 (1992).
58. Plümper, O., Røyne, A., Magrasó, A. & Jamtveit, B. The interface-scale mechanism of reaction-induced fracturing during serpentinization. *Geology* **40**, 1103–1106 (2012).
59. Melosh, B. L. Fault Initiation in Serpentinite. *Geochem Geophys Geosyst* **20**, 2626–2646 (2019).
60. Uno, M. & Kirby, S. Evidence for multiple stages of serpentinization from the mantle through the crust in the Redwood City Serpentinite mélange along the San Andreas Fault in California. *Lithos* **336–337**, 276–292 (2019).
61. Malvoisin, B. & Brunet, F. Water diffusion-transport in a synthetic dunite: Consequences for oceanic peridotite serpentinization. *Earth and Planetary Science Letters* **403**, 263–272 (2014).
62. Li, D., Beyer, C. & Bauer, S. A unified phase equilibrium model for hydrogen solubility and solution density. *International Journal of Hydrogen Energy* **43**, 512–529 (2018).
63. Manning, C. E. & Ingebritsen, S. E. Permeability of the continental crust: Implications of geothermal data and metamorphic systems. *Reviews of Geophysics* **37**, 127–150 (1999).

Natural hydrogen generation from serpentinized mantle rocks

Rodolfo Christiansen[1]*, Mohamed Sobh[1], Christian Ostertag-Henning[2], Guido Gianni[3], Nicolas Saspiturry[4], Sebastien Chevrot[5,6], Victoria Langenheim[7], Javier Garcia-Pintado[8], Gerald Gabriel[1,9]

[1] LIAG Institute for Applied Geophysics, 30655 Hannover, Germany
[2] Federal Institute for Geosciences and Natural Resources (BGR), 30655 Hannover, Germany
[3] Czech Academy of Sciences, Institute of Geophysics, 14100 Prague, Czechia
[4] Géosciences Montpellier, Université de Montpellier, CNRS, 34095 Montpellier, France
[5] Université de Toulouse, UPS-OMP, IRAP, 31028 Toulouse, France
[6] GET, CNRS, Observatoire Midi Pyrénées, Université de Toulouse, UMR5563, F-31400 Toulouse, France
[7] U.S. Geological Survey, Moffett Field, California 94035, USA
[8] MARUM - Center for Marine Environmental Sciences, University of Bremen, 28359 Bremen, Germany
[9] Leibniz University Hannover, Institute of Earth System Sciences, 30167 Hannover, Germany

*email: Rodolfo.Christiansen@liag-institut.de or rodo_christiansen@hotmail.com

Abstract

Mantle rocks undergoing serpentinization can generate significant amounts of natural hydrogen, yet the rates and controlling processes remain poorly constrained. Here, we demonstrate that in two distinct mantle rock types, the fertile lherzolites of the Western Pyrenees and the depleted harzburgites of Northern California, continuous water infiltration may sustain hydrogen generation only at relatively low rates of ~0.1 to ~0.5 tonnes H_2 yr^{-1} km^{-3} of reactive rock. When integrated over the full reactive volumes in the areas, this corresponds to total production rates of ~300 to ~600 tonnes H_2 yr^{-1}. By combining three-dimensional geophysical inversion with numerical modelling of fluid-rock processes, we show that hydrogen generation rates are mainly limited by H_2 saturation in the fluid and reaction kinetics. Under these constraints, hydrogen generation in mantle-derived serpentinization systems proceeds slowly, making rapid large-scale replenishment unlikely and suggesting that large, economically relevant accumulations, if present, would require timescales of thousands to tens of thousands of years to develop.

Introduction

Serpentinization is an abiotic reaction involving the hydration of ultramafic rocks, in which Fe(II) in primary minerals is oxidized to Fe(III) in secondary phases, reducing hydrogen atoms in water molecules to molecular hydrogen (H_2). This process is one of the primary natural mechanisms for hydrogen generation[1–5]. The generation rate of molecular hydrogen (hereafter referred to as hydrogen or H_2) and the rate of serpentinization are highly dependent on temperature, with maximum rates between 200°C and 300°C[2,6]. This mineral replacement reaction also affects the petrophysical properties of the rocks by decreasing density and seismic velocities, associated most often with increasing magnetic susceptibility. These contrasts allow serpentinites to be distinguished from fresh mantle rocks based on their geophysical characteristics, such as gravity, magnetic, and seismic properties[7–9].

In recent years, natural hydrogen has emerged as a potential low-carbon energy resource[10]. However, growing evidence suggests that hydrogen generation associated with mantle rocks serpentinization proceeds over long timescales, with limited generation rates, even in the most favourable conditions, such as deep olivine serpentinization[11,12]. Major obstacles to quantifying natural hydrogen generation include the inaccessibility of deep crustal environments, a limited understanding of water availability, and uncertainties in reaction kinetics under low water-to-rock ratios (W/R), defined as the fluid-to-rock mass ratio. Serpentinization is nevertheless associated with some of the largest natural hydrogen occurrences worldwide, with high hydrogen concentrations measured in emanating gases and associated flow-rate estimates of several hundred tonnes per year[13,14]. In contrast, model-based estimates remain highly uncertain. For example, in the Western Pyrenees, reported values range from several hundred thousand[15] to several million tonnes of hydrogen produced per year[16]. These high estimates largely reflect approaches that quantify cumulative hydrogen generation potential, often

assuming efficient and sustained fluid-rock interaction and neglecting explicit constraints related to fluid flows, reaction kinetics, and hydrogen saturation in the fluids. As a result, such values are not directly comparable to observed hydrogen flow rates in similar geological settings.

Here, we present an open-source modelling framework for quantifying natural H_2 generation during serpentinization that explicitly constrains geological parameters within well-defined petro-physico-chemical boundary conditions. Hydrogen generation is evaluated using a sequential, quasi-dynamic modelling framework in which thermodynamic equilibrium is assumed locally for each fluid-rock interaction event. Sustained hydrogen production rates emerge from the progression of these equilibrated states when fresh water is supplied, hydrogen accumulates in solution, and the serpentinization front advances under explicit kinetic limitations. Fluid transport within the source rocks is parameterized through a combination of fracture-controlled flow and diffusive exchange. Feedbacks arising from fluid saturation and lithology-dependent rock alteration are explicitly resolved, together with rock-specific petrophysical properties, including mineral-bound water uptake, pressure-dependent hydrogen solubility, water renewal rates, and porosity-controlled reaction-front propagation velocity. We apply this model to two different geological areas, the Western Pyrenees and Northern California, selected for their good data availability and contrasting mantle rock compositions. The first case involves relatively fertile mantle rocks rich in olivine and orthopyroxene[17,18], whereas the second is dominated by less reactive rocks that represent residues of high degrees of partial melting[19]. By integrating geological, geophysical, and physico-chemical constraints, we provide first-order estimates of hydrogen production rates and show that, at large scales, mantle-derived natural hydrogen behaves as a slowly replenished geological resource.

Results

Western Pyrenees. The Western Pyrenees (Fig. 1a, b) consist of a tectonic wedge, where mantle-derived rocks, mainly spinel lherzolites[17,18], were exhumed during middle Cretaceous rifting[20,21], and tectonically uplifted to their current shallow position (~10 km) as a result of Eocene-Oligocene convergence, which ended about 25 Ma ago[22–25]. Since then, tectonic conditions have remained relatively stable, which provides a prolonged window that kept the exhumed mantle rocks within optimum pressure-temperature conditions for serpentinization[15,16,26]. This process is further supported by the occurrence of microseismicity around a high-velocity mantle body, which is interpreted as evidence of ongoing hydration[27,28]. Evaporites and claystones within the overlying sedimentary sequence may act as a trap for the hydrogen being produced[29]. Soil gas measurements (Fig. 1b) confirm the presence of hydrogen seepage in the vicinity of major thrust faults, indicating upward migration toward the surface[23]. Gravity and magnetic anomalies align with a body of variably serpentinized lithospheric mantle at an unusually shallow depth[26].

Geophysical inversion of residual isostatic Bouguer and residual reduced-to-the-pole (RTP) magnetic anomalies (Fig. 1c, d), constrained by seismic velocity models[28], and combined with thermal modelling (Fig. 1d), indicates the presence of ~2,700 km^3 of ultramafic material serpentinized over ~37–57% of its volume (Fig. 1e). Of this volume, ~630 km^3 fall within the temperature range where hydrogen generation occurs according to our thermodynamic calculations, whereas ~2,100 km^3 occur at temperatures higher than the modelled hydrogen production window (refer to H2 production database in Methods for details). These rocks are spatially associated with a second group of ~2,400 km^3 of mantle rocks characterized by a minimal serpentinization of ~10% (green unit in Fig. 1c and Fig. 1e, with volume limited by the 20 km depth extent of the model). Maximum H_2 production, as a function of pressure and

temperature, was computed for these rocks at a fixed water-to-rock ratio of 0.16 using thermodynamic calculations for a generic lherzolite in equilibrium with pure water (Fig. 2a).

The range of water input (1×10^4 to 1×10^7 L day^{-1}; shaded area in Fig. 2b) was constrained on one side by the maximum transport capacity of a 50 km long fault zone, selected to match the lateral extent of the ultramafic unit, and on the other side by the flow required to serpentinize 47% of the mantle rock volume over 25 Ma, assuming time-averaged fluxes (refer to Deep water flow capacity estimation in Methods for more information). Within this range, an intermediate flow rate of 5×10^5 L day^{-1} was selected as a representative scenario. Across the full range of possible flow rates, annual hydrogen production as dissolved H_2 is estimated to span ~0.01–9.5 tonnes H_2 yr^{-1} km^{-3} of reactive rock, corresponding to ~10–7,000 tonnes H_2 yr^{-1} when integrated over the entire reactive rock volume. For the selected intermediate scenario, production reaches ~0.51 ± 0.16 tonnes H_2 yr^{-1} km^{-3} of reactive rock, equivalent to 308 ± 88 tonnes H_2 yr^{-1} (Fig. 2a, b and Supplementary Table 5).

Additional simulations (Fig. 2b; Supplementary Table 7) were performed to assess sensitivity to water supply by varying inflow rates (10^1–10^{10} L day^{-1}; Fig. 2b). At low inflows ($<10^4$ L day^{-1}), limited water renewal leads to large pore turnover and saturation times ($>10^4$–10^5 days), resulting in negligible hydrogen production (<10 tonnes H_2 yr^{-1}) constrained by water availability. At intermediate flows (~10^4–10^6 L day^{-1}), pore turnover times decrease while saturation times become shorter than turnover times, causing dissolved H_2 to reach near-solubility before pore water is renewed and suppressing further hydrogen generation due to limited product removal. At higher inflows ($>10^6$ L day^{-1}), pore turnover becomes very short and hydrogen generation initially increases as larger fluid volumes pass through the reactive zone, reaching a broad maximum of ~8,500 tonnes H_2 yr^{-1} when turnover and saturation timescales become comparable (refer to Supplementary Table 7). At the highest flow rates,

hydrogen generation decreases as fluids are advected through the system before substantial reaction progress can occur, with pore-water turnover rates exceeding 100% per day (i.e., the effective pore-water volume is renewed more than once per day; Fig. 2b).

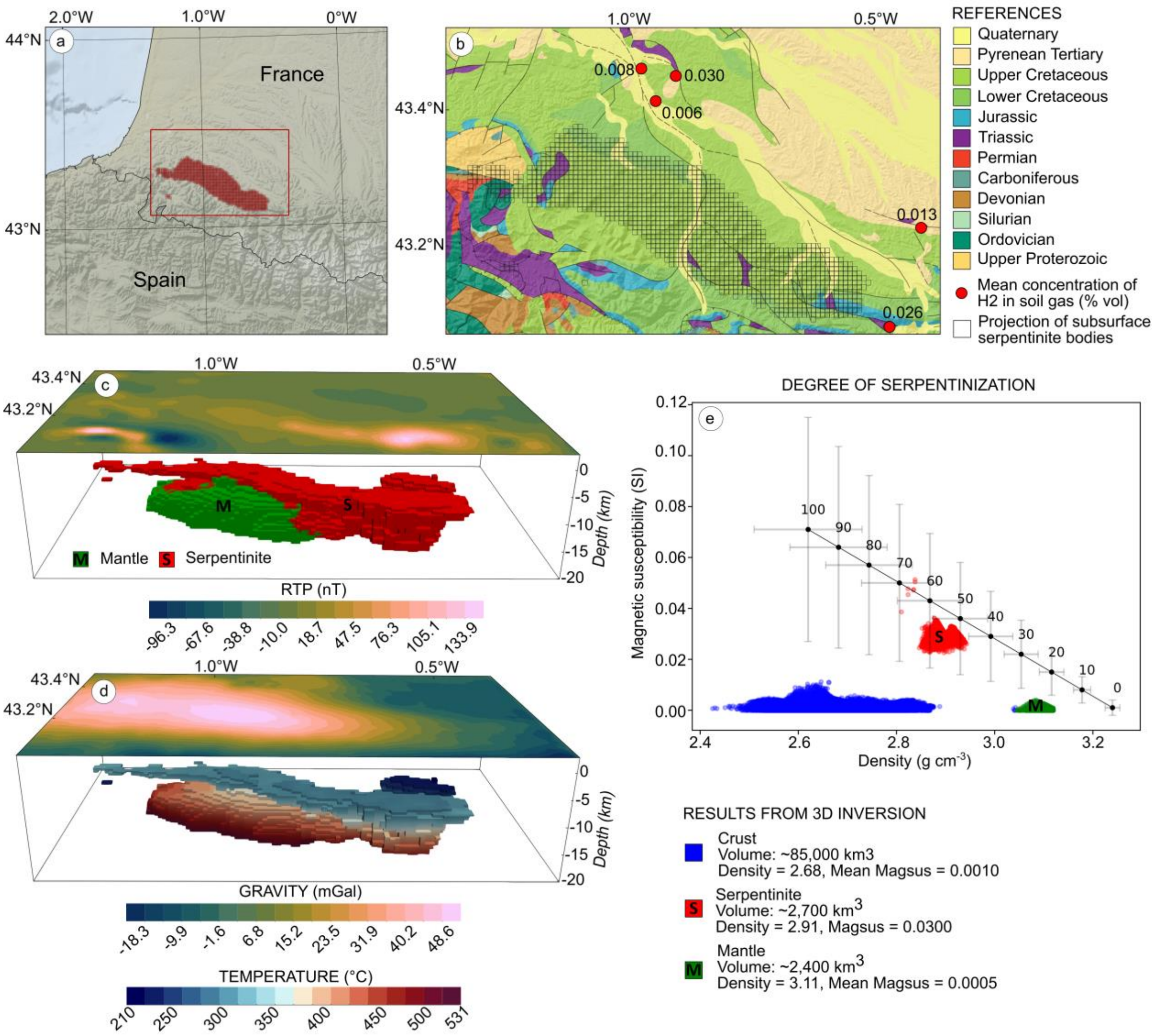

Fig. 1: Results of source rock volumes for the Western Pyrenees. a) Index map of study area (red box). The red polygons show modelled serpentinite extent projected on to the surface. b) Age of geologic units in the study area (modified from Ref.[30]). Polygons show modelled serpentinite blocks projected on to the surface. Red circles represent H_2 measurements (%) in soil gases (modified from Ref.[23]). c) Reduced to the pole magnetic anomalies (RTP) for the area (modified from Ref.[31] and Ref.[26]) with 3D blocks showing rock classification based on joint inversion of gravity and magnetic data. d) Residual Bouguer gravity anomalies for the area (modified from Ref.[26]) with 3D blocks showing temperature distribution. e) Results of the Gaussian Mixture Model (GMM) indicating the degree of serpentinization based on density and magnetic susceptibility results. Values are projected to the closest serpentinization point in the line. Crustal rocks correspond to an averaged petrophysical representation of the heterogeneous continental crustal assemblage included in the model (e.g., granitoids, gneisses, schists, locally mafic to ultramafic rocks, and metasedimentary units).

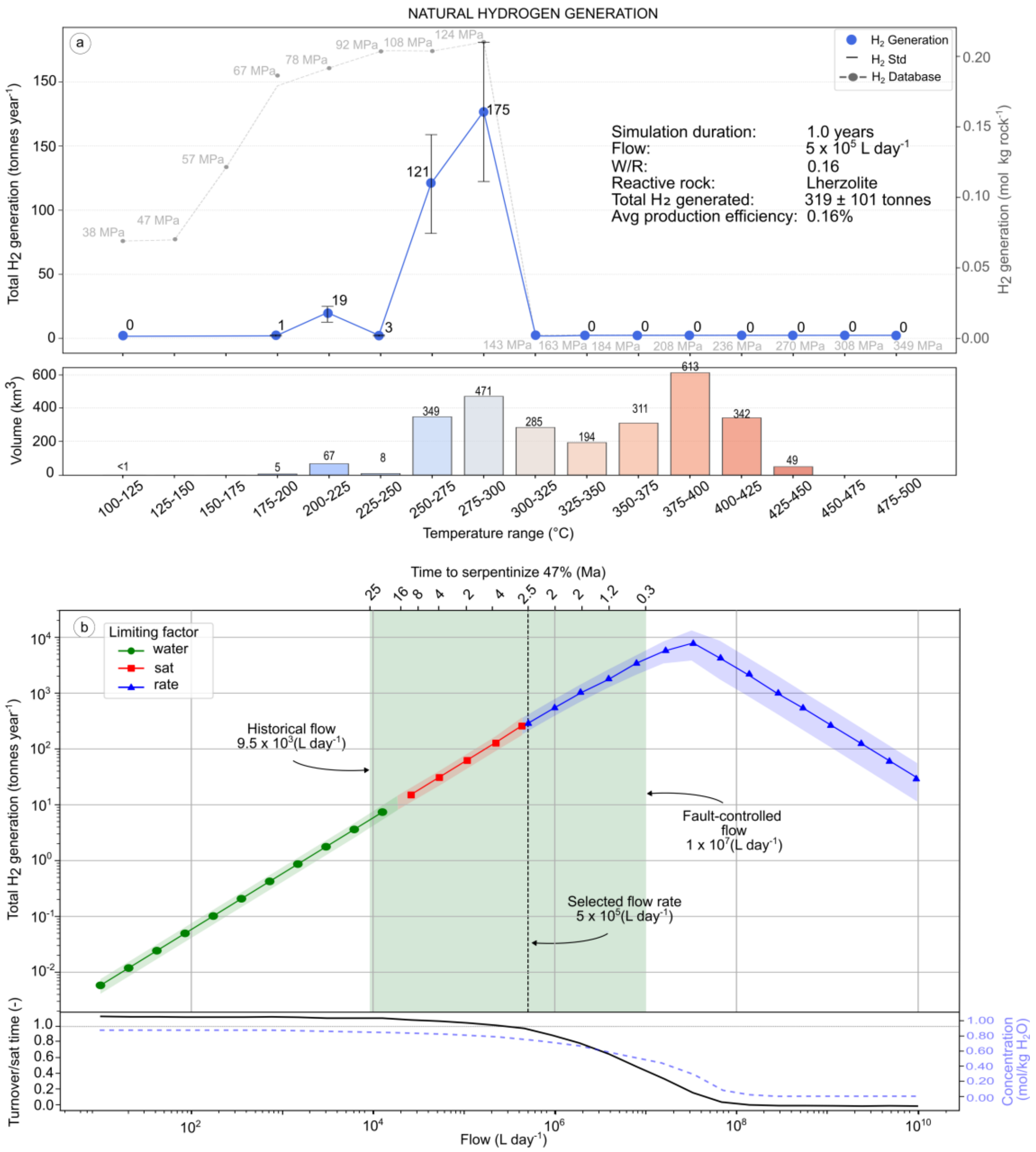

Fig. 2. Overview of $H_2(aq)$ generation estimates under contrasting water availability scenarios for the Western Pyrenees. a) Results of natural hydrogen generation and ±1 standard deviation of the values (blue dots in the upper rectangle and black error lines), for a flow of $5x10^5$ L day^{-1}, showing the corresponding hydrogen yield per kilogram of rock (in grey) and the volumes of partially serpentinized rocks (lower rectangle, fresh mantle not included). Numbers next to the blue points indicate the H_2 production in tonnes $year^{-1}$ and numbers next to the grey points represent pressure values in MPa. For visualization purposes, hydrogen production values were rounded to the nearest whole number. Production efficiency relative to unconstrained generation conditions (refer to Supplementary Table 3). W/R denotes the water-to-rock mass ratio. b) Total annual hydrogen production plotted against daily water flow. Marker and colour indicate the dominant limiting factor, and the shaded envelope represents ±1 standard deviation (Note: standard deviations are shown on a logarithmic scale, visually distorting the uncertainty envelope). Green dot for water limitation for production below 10 tonnes $year^{-1}$, red squares for saturation of H_2 in the fluid and blue triangles for kinetics (e.g., serpentinization front velocity). The shaded area highlights the plausible flow range, spanning from historically inferred values to those controlled by fault zone permeability. The dashed line at 5×10^5 L day^{-1} marks the selected intermediate scenario. The lower panel shows the ratio between pore turnover time and saturation time (black line): values greater than 1 indicate that H_2 saturation is reached before pore-water renewal, whereas values lower than 1 indicate faster pore-water renewal. A ratio close to 1 marks conditions where both processes occur on comparable timescales. The panel also reports the average dissolved H_2 concentration (blue dashed line). The reported concentration is computed using only the water that effectively enters the reactive pore system (diffusive + fracture inflow), not the total imposed flow.

Northern California. The north-western part of the United States of America, north of San Francisco (Fig. 3a, b), hosts extensive, shallow harzburgite-rich ophiolite complexes[19]. These include the Coast Range ophiolite, which likely originated in a supra-subduction zone setting and was emplaced onto continental crust during Late Jurassic tectonic events[32,33]. Their present crustal position reflects not only Jurassic emplacement but also major Cretaceous tectonic imbrication and uplift since approximately 90 Ma[34]. The presence of coarse, unfoliated bastite (orthopyroxene pseudomorphs) in some areas, however, indicates that strong tectonic overprinting or deep burial did not occur pervasively throughout the complex[35]. Great Valley forearc data indicate that Jurassic-Cretaceous syn-sedimentary extension, followed by Cenozoic folding, uplift, and strike-slip faulting linked to the shift from subduction to a transform boundary, reshaped these structures[36,37].

Currently, these complexes are at depths where temperatures and active fault systems are favourable for serpentinization, with faults providing permeable pathways for fluids to hydrate the ultramafic rocks[19]. Large-amplitude positive magnetic anomalies (Fig. 3c) align with the serpentinite bodies mapped in the region, sometimes acting as a proxy to infer zones of aseismic slip[38]. In contrast, gravity highs (Fig. 3d) reflect the presence of relatively dense crustal units beneath thinner sedimentary cover. Surface measurements showing elevated hydrogen concentrations (Fig. 3b) and thus ongoing serpentinization processes, suggest active fluid migration from depth[39,40]. Geophysical inversion of residual isostatic Bouguer and residual reduced to the pole magnetic anomalies, guided by the lithospheric geometry derived from P-wave tomographic models[41], and integrated with thermal modelling (Fig. 3c, d), reveals the presence of ~12,000 km^3 of highly serpentinized rocks. Of this volume, ~4,500 km^3 fall within the temperature range predicted by our thermodynamic framework to sustain hydrogen generation (refer to H2 production database in Methods for details). In addition, ~7,500 km^3 of ultramafic rocks lie outside this hydrogen-producing temperature window. Results also suggest

that the degree of serpentinization of the mantle rocks in Northern California is very high, with average values approaching complete serpentinization (Fig. 3f). Maximum H_2 production as a function of pressure and temperature was computed for these rocks at a fixed water-to-rock ratio of 0.16 using thermodynamic calculations for a generic harzburgite in equilibrium with pure water (Fig. 4a).

The expected range of fluid input (~10^6 to ~5×10^7 L day^{-1}; highlighted in Fig. 4b) combines constraints from two independent considerations. The upper bound reflects the estimated maximum water-delivery capacity of a 250 km long fault system, consistent with the mapped extent of the ultramafic units (refer to Deep water flow capacity estimation in Methods for more information). In contrast, the lower bound represents the minimum sustained, time-averaged fluid flux required to achieve near-complete serpentinization over a ~90 Ma timescale, assuming continuous fluid circulation. An intermediate reference flow rate of 2×10^6 L day^{-1} was selected to represent present-day fluid circulation conditions. Across this plausible range of flow rates, annual hydrogen production as dissolved H_2 spans ~0.11–1.11 tonnes H_2 yr^{-1} km^{-3} of reactive rock, corresponding to ~500–5,000 tonnes H_2 yr^{-1}, when integrated over the entire reactive volume. For the intermediate flow scenario, production is estimated at ~0.11 ± 0.03 tonnes H_2 yr^{-1} km^{-3}, equivalent to 515 ± 168 tonnes H_2 yr^{-1} (Fig. 4a, b and Supplementary Table 6).

Sensitivity to water supply was tested by varying inflow rates (10^1–10^9 L day^{-1}; Fig. 4b and Supplementary Table 8) at fixed water-to-rock ratio (0.16). At very low inflow rates (<10^4 L day^{-1}), fluid renewal within the reactive volume is negligible, resulting in long residence times and hydrogen production remaining below ~10 tonnes yr^{-1}. With increasing inflow, fluid circulation becomes progressively more efficient, allowing hydrogen to be transported away from the reaction sites and leading to an increase in production. At intermediate to high inflow

rates (≳10^5–10^7 L day^{-1}), hydrogen generation is no longer limited by water availability but becomes controlled by reaction kinetics. Under these conditions, production reaches a broad maximum of ~5,000 tonnes H_2 yr^{-1}, before declining at the highest flow rates, as rapid pore-water renewal limits fluid residence time and prevents substantial hydrogen generation.

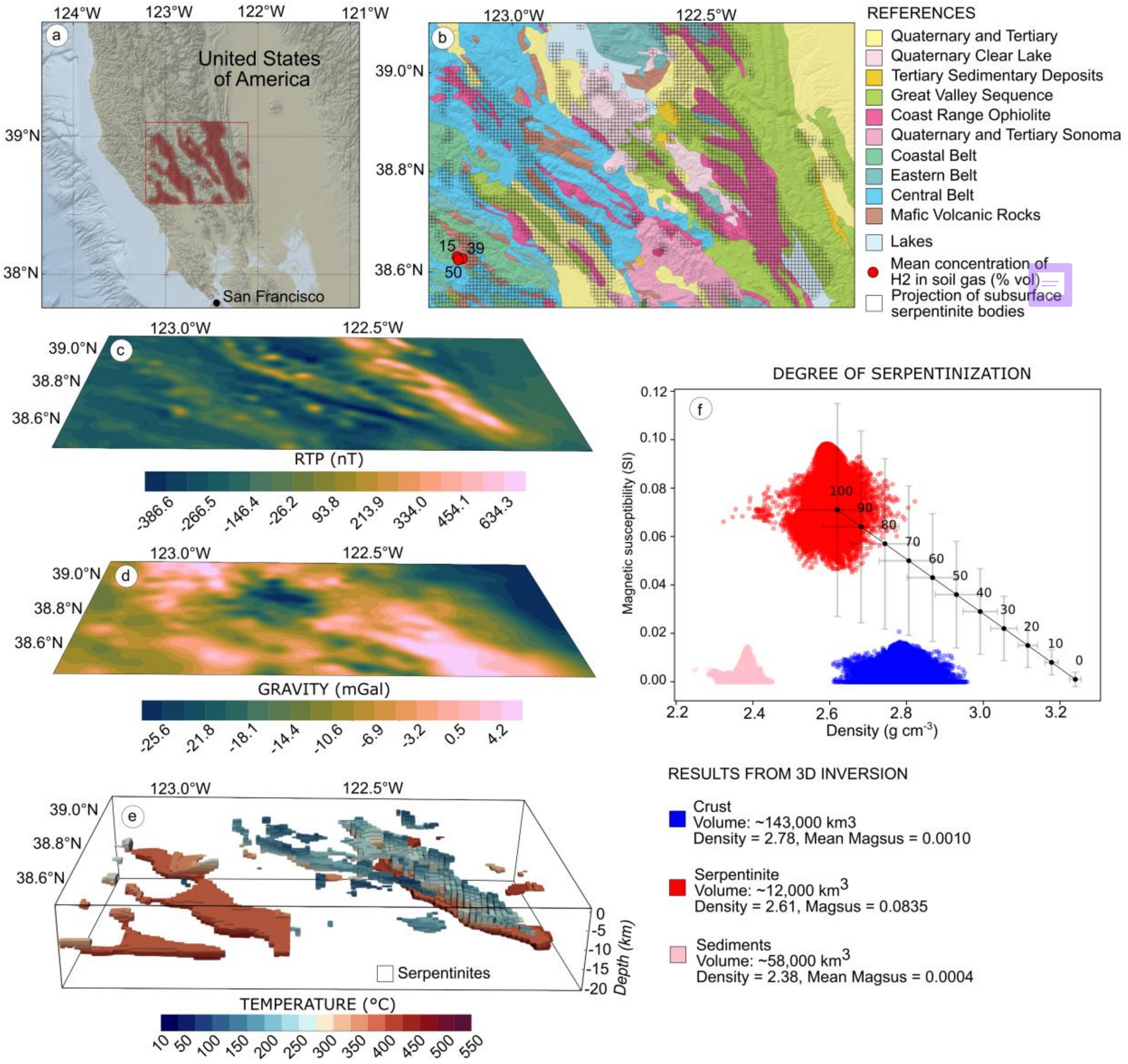

Fig. 3: Results of source rock volumes for Northern California. a) Index map of study area (red box). The Red polygons show modelled serpentinite extent projected on to the surface. b) Geologic units in the study area (modified from Ref.[38]). Polygons show modelled serpentinite blocks projected on to the surface. Red circles represent H_2 measurements (%) in soil gases (modified from Ref.[40]). c) Reduced to the pole magnetic anomalies (RTP) for the area (modified from Ref.[38]). d) Residual gravity anomalies for the area (modified from Ref.[38]). e) 3D blocks showing temperature distribution for the serpentinites. f) Results of the Gaussian Mixture Model (GMM), indicating the degree of serpentinization based on density and magnetic susceptibility results. Values are projected to the closest serpentinization point in the line. Crustal rocks and sediments represent averaged petrophysical descriptions of the heterogeneous crustal assemblage and the sedimentary cover included in the model (e.g., metasedimentary and metavolcanic rocks, greywackes, cherts, serpentinites and locally mafic rocks; unconsolidated to weakly lithified basin-fill and forearc sedimentary deposits).

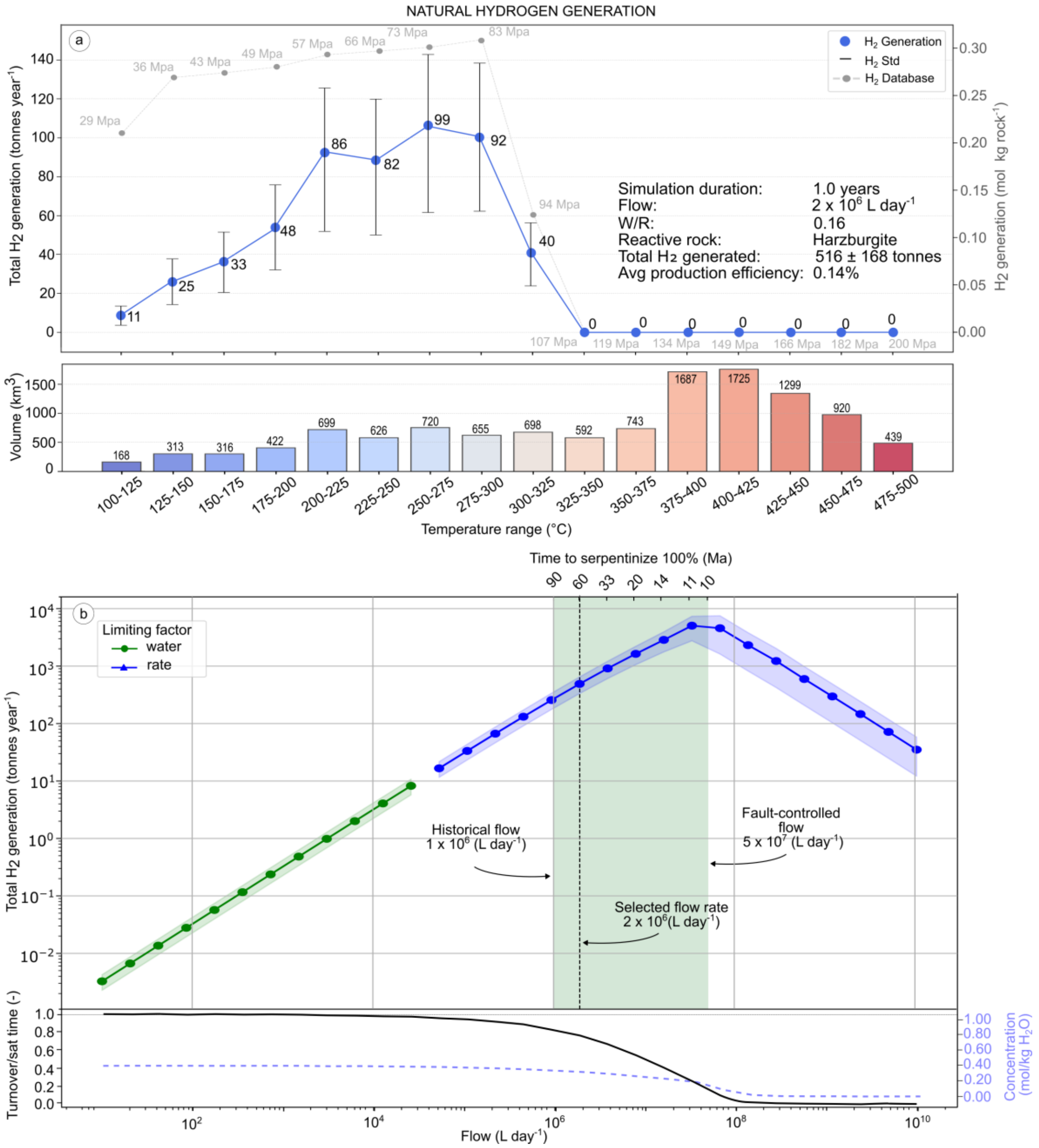

Fig. 4. Overview of H_2(aq) generation estimates under contrasting water availability scenarios for Northern California. a) Results of natural hydrogen generation and ±1 standard deviation of the values (blue dots in the upper rectangle and black error lines), for a flow of 2 x 10^6 L day^{-1}, showing the corresponding hydrogen yield per kilogram of rock (in grey) and the volumes of partially serpentinized rocks (lower rectangle). Numbers next to the blue points indicate the H_2 production in tonnes $year^{-1}$ and numbers next to the grey points represent pressure values in MPa. For visualization purposes, hydrogen production values were rounded to the nearest whole number. Production efficiency relative to unconstrained generation conditions (refer to Supplementary Table 4). W/R denotes the water-to-rock mass ratio. b) Total annual hydrogen production plotted against daily water flow. Marker colour indicates the dominant limiting factor, and the shaded envelope represents ±1 standard deviation (Note: standard deviations are shown on a logarithmic scale, visually distorting the uncertainty envelope). Green dot for water limitation for production below 10 tonnes $year^{-1}$, red squares for solubility (e.g., saturation of H_2 in the fluid) and blue triangles for kinetics (e.g., serpentinization front velocity). The shaded area highlights the plausible flow range, spanning from historically inferred values to those controlled by fault zone permeability. The dashed line at 2 x 10^6 L day^{-1} marks the selected intermediate scenario. The lower panel shows the ratio between pore turnover time and saturation time (black line): values greater than 1 indicate that H_2 saturation is reached before pore-water renewal, whereas values lower than 1 indicate faster pore-water renewal. A ratio close to 1 marks conditions where both processes occur on comparable timescales. The panel also reports the average dissolved H_2 concentration (blue dashed line). The reported concentration is computed using only the water that effectively enters the reactive pore system (diffusive + fracture inflow), not the total imposed flow.

Model sensitivity. Univariate sensitivity analyses based on 5,000 Monte Carlo realizations per parameter for both the unconstrained and constrained hydrogen generation models (refer to Supplementary Tables 9 and 10) yield consistent sensitivity patterns. The unconstrained (no-saturation) model shows strong and nearly linear sensitivity to multiple parameters that control hydrogen generation. Variations in serpentinization front velocity (0.4–1.6×) produce changes of ~120% relative to the baseline, while intrinsic production rate (0.5–1.5×), effective reactive surface area (0.6–1.4×), reactive volume (0.8–1.2×), and serpentinization correction (0.8–1.2×) result in changes of ~100%, ~80%, and ~40%, respectively. In contrast, in the constrained model, only parameters directly controlling hydrogen saturation in the fluid influence the amount of hydrogen finally delivered. Varying mean pressure and hydrogen solubility within 0.7–1.3× of their reference values results in changes corresponding to ~57–58% of the baseline production. All other parameters, including reactive volume (0.8–1.2×), serpentinization degree (0.8–1.2×), fracture spacing (0.5–1.5×), permeability (0.8–1.2×), intrinsic production rate (0.8–1.2×), block size (0.5–1.5×), and reactive rock mass (0.4–1.6×), produce negligible effects on total hydrogen output over their tested ranges.

Discussion

Our three-dimensional models of the Western Pyrenees (Fig. 1c, d) indicate that exhumed mantle rocks extend from depths of ~8 km to at least 20 km, corresponding to the lower limit of our model. These ultramafic rocks can be subdivided into a deeper domain characterized by a low degree of serpentinization (~10%), and a shallower domain that exhibits substantially higher alteration levels (~37–57%). This subdivision is largely consistent with the temperature window over which our thermodynamic calculations predicts hydrogen-producing serpentinization (Fig. 5b), and aligns with previous interpretations based on gravity, magnetic, and P-wave velocity models[26,28], as well as petrophysical modelling[16]. In this area, natural hydrogen generation calculated under the most likely fluid flow conditions inferred for the

study area is on the order of a few hundred tonnes of H_2 per year, corresponding to a normalized production rate of ~0.51 ± 0.16 tonnes H_2 yr^{-1} km^{-3} of reactive rock. Under these settings, dissolved H_2 accumulates to near-solubility levels before pore-water renewal occurs, progressively reducing the thermodynamic driving force for further hydrogen generation. In contrast to our findings, previous studies have estimated a theoretical hydrogen production potential ranging from several hundred thousand[15] to several tens of millions of tonnes per year[16]. Because these estimates are based solely on the volume of reactive rocks and do not account for water availability or fluid saturation, they are only comparable to the results obtained with our unconstrained model (refer to Supplementary Tables 3 and 4).

For Northern California, our results also indicate representative long-term hydrogen generation on the order of a few hundred tonnes of H_2 per year, as evaluated under flow conditions defined within the explored parameter space. When normalized to the volume of reactive rock, this corresponds to a production rate of ~0.11 ± 0.03 tonnes H_2 yr^{-1} km^{-3} of reactive rock. In contrast to the Western Pyrenees, hydrogen generation in this system is primarily limited by reaction kinetics, which reflects the near-complete serpentinization of the mantle peridotites. Seismic velocity models[41], together with surface observations of serpentinites and two-dimensional magnetic modelling[38], consistently indicate widespread and advanced alteration of the ultramafic rocks. As a result, the volume of remaining reactive material is comparatively limited, but extensive fracturing maintains a large effective pore space, allowing a high fluid circulation that prevents rapid saturation of the reacting fluids with dissolved hydrogen. Therefore, generation is controlled mainly by the rate at which new reactive surfaces and porosity can be generated, with fluid saturation playing only a secondary role.

Interpretation of these results requires consideration of several first-order simplifications inherent to the modelling approach. Fluid-rock reactions are represented as a sequence of local

thermodynamic equilibrium steps, such that short-lived kinetic limitations and fine-scale spatial heterogeneities are not explicitly resolved. Hydrogen production is therefore estimated as a quasi-dynamic, time-averaged rate rather than as instantaneous or peak fluxes. Moreover, thermodynamic calculations consider only H_2 generation associated with magnetite formation and assume pure water as the reacting fluid, although experimental studies indicate that increased salinity and reduced water activity can significantly slow serpentinization reaction rates[4]. In addition, fluids are assumed to be uniformly distributed throughout the reactive rock volume, whereas natural systems exhibit highly heterogeneous, fracture-oriented fluid access. These assumptions have counteracting effects on predicted hydrogen generation: pure-water conditions and homogeneous fluid access systematically bias hydrogen production toward higher values, while limiting H_2 generation to magnetite-forming reactions alone biases estimates toward lower total yields. Consequently, the derived production rates should be interpreted as first-order magnitudes rather than precise in-situ values. Nevertheless, our average production estimates of ~0.31 ± 0.08 tonnes H_2 yr^{-1} km^{-3} of reactive rock fall within the range of natural hydrogen fluxes inferred from other crustal settings, including the Bulqizë ophiolite (Albania) and the Zambales ophiolite (Philippines), which correspond to normalized values of ~0.2 and ~1.4 tonnes H_2 yr^{-1} km^{-3}, respectively[13,14,42], indicating that the model captures the correct order of magnitude of natural hydrogen generation systems.

The present scenario likely does not apply to the earliest stages of serpentinization. Although water may have been available, the initially low degree of fracturing in fresh ultramafic rocks would have limited fluid access and reactive surface area, and this would have resulted in weak fluid-rock interaction and low hydrogen production. As serpentinization progresses, together with tectonic deformation and reaction-induced cracking, fracture density and permeability increase, creating additional fluid pathways and exposing progressively larger reactive surfaces to fluids. This is consistent with laboratory evidence, which indicate that fracture-controlled

flow is the dominant mechanism for fluid delivery in ultramafic settings[43]. This structural and chemical evolution likely led to a gradual acceleration of serpentinization and associated hydrogen generation. However, maintaining such conditions continuously would imply complete hydration of the ultramafic volume well within the time elapsed since mantle exhumation. The modelled serpentinization rates should therefore be interpreted as effective rates representative of periods with sustained fluid access and enhanced fracturing[44]. In natural systems, fluid supply and permeability are expected to be intermittent and spatially localized, so elevated reaction rates are likely to occur only during short-lived, fracture-controlled fluid-infiltration events, separated by longer periods of limited or no reaction.

In our modelling framework, hydrogen is generated and transported exclusively in the aqueous phase, and the formation, migration and trapping of a free gas phase are not explicitly simulated. Partial exsolution may occur during upward migration, as pressure decreases and hydrogen solubility is reduced, but considerable accumulations are expected only where favourable structural conditions exist, such as effective traps and low-permeability seals. Higher fluid fluxes do not necessarily result in larger accumulations, as increased dilution in the aqueous phase can delay gas-phase separation and long-term retention. In line with recent play-based assessments of natural hydrogen systems[45], hydrogen prospectivity reflects the efficiency of migration, phase separation, and trapping rather than generation rates alone. The production levels estimated here fall below thresholds typically required for large industrial offtake and are therefore compatible with localized applications unless exceptional trapping conditions permit gas-phase accumulation. This conclusion does not rule out other geologic sources or processes that may operate over shorter timescales and could sustain higher production rates. Integrating hydrogen generation models with reactive transport and migration simulations would provide a more quantitative basis to evaluate the potential for phase

separation and long-term retention, thereby better constraining the viability of subsurface hydrogen accumulations.

Methods

Subsurface modelling and petrophysical constraints. Initial three-dimensional models of the primary subsurface rock units (e.g., mantle rocks, serpentinized rocks, basement rocks) are constructed based on 2D sections. These initial models are not intended to represent detailed geological structures and serve only as initial guides to help the inversion converge toward geologically plausible solutions. Petrophysical data are parametrized from seismic velocity models (refer to Supplementary Tables 1 and 2). Observed gravity and magnetic data are jointly inverted for density and magnetic susceptibility using a Petrophysically Guided Inversion (PGI) framework implemented in SimPEG[46,47] in which petrophysical information is incorporated through Gaussian mixture models linking lithological classes to admissible ranges of physical properties. For both study areas, density and magnetic susceptibility are constrained within physically plausible bounds, while initial models and background values are used only to initialize the inversion.

The inversion is formulated as a regularized optimization problem in which density and magnetic susceptibility are the control variables and the objective function minimizes the misfit between observed and predicted gravity and magnetic anomalies while enforcing petrophysical consistency and spatial smoothness. Regularization assumes laterally and vertically coherent property distributions and penalizes strong gradients in the three spatial directions through first- and second-order smoothness terms. The trade-off between data misfit and regularization is controlled and an iterative cooling schedule that progressively relaxes regularization during the inversion. Physical bounds are imposed on the recovered properties depending on lithology. Resulting cells consistent with serpentinized rocks are identified based on their joint density-

susceptibility values within the PGI framework (Figs. 1e and 3f), and the volumes of these units are subsequently quantified. A brief description of the inversion parameters used for each case study is provided in Supplementary Tables 1 and 2, and the full set of parameters used in the numerical experiments can be found in the configuration files made available in the Data Availability section.

Thermal modelling. Subsurface temperature distributions (Figs. 5a and 6a) are calculated by solving the steady-state heat conduction equation using the finite volume method, as implemented in the FiPy package[48]. At each depth interval, thermal properties are simulated using piecewise-linear functions that represent the natural variability of the thermal conductivity with depth. A fixed temperature at the surface (i.e., Dirichlet boundary condition), and a background heat flow at the base (i.e., Neumann boundary condition) are imposed (refer to Supplementary Tables 1 and 2). The previously identified volumes of partially serpentinized rocks are populated with the temperature distribution. Although ultramafic rocks are generally considered capable of generating hydrogen between 100 and 500 °C, only a narrower temperature interval is relevant here, as H_2 production is restricted to thermodynamically stable magnetite-forming reactions (refer to H_2 production database).

H_2 production database. Thermodynamic equilibrium calculations for hydrogen production were compiled for two representative ultramafic lithologies reacting with pure water: lherzolite (Al_2O_3 4.46, CaO 2.65, FeO 7.86, MgO 40.13, SiO_2 44.75 wt%) and harzburgite (Al_2O_3 3.13, CaO 1.25, FeO 8.69, MgO 43.53, SiO_2 43.33 wt%). Potential H_2 production reaches around 250 mmol kg rock^{-1} for lherzolite and 300 mmol kg rock^{-1} for harzburgite (Figs. 5b and 6b). These thermodynamic equilibrium results are taken directly from Ref.[49] and were used as input constraints in this study. The original calculations were performed using Gibbs free energy minimization with the Theriak-Domino software[50]. In these, the thermodynamic databases

JUN92d.bs[51] and TC-DS633[52,53] were evaluated. Both resulted in similar H2 production rates at equilibrium for the evaluated bulk compositions, and the calculations from JUN92d.bs were finally applied. However, none of these databases included recent solid solution models for Fe(III)-bearing serpentines[54], such that hydrogen generation is attributed only to Fe(III) iron allocated to magnetite formation. The water-to-rock ratio (W/R) considered in the database spans 0.0–0.17, beyond which no further metamorphic transformation occurs and hydrogen production remains unchanged.

Serpentinization framework. A correction was applied to account for the loss in reactive surface due to the progressive volume replacement of peridotite by serpentinite (Figs. 5c and 6c), assuming serpentinization progresses inward from the periphery of each block and accounting for unaltered olivine cores. As a result, the hydrogen generation potential of each block is reduced compared to an entirely unaltered peridotite volume, since only the remaining unaltered fraction is available for ongoing serpentinization reactions. The volumetric serpentinization degree is estimated by comparing the average density and magnetic susceptibility weighted by the proportions of altered and unaltered material to reference datasets[7–9,55,56]. To conservatively account for isolated or kinetically-inhibited volumes resistant to complete alteration at the core[57,58], a residual value of 2% is adopted in this study.

Fracture network and serpentinization front velocities. Reactive rock blocks with sub-meter to meter-scale dimensions are defined based on fracture spacing observed in outcrops[59–63]. To estimate the progression velocities of the serpentinization front across temperature ranges (Figs. 5d and 6d), we adopt a scaling approach based on hydrogen production rates. A reference temperature-pressure condition with a known front velocity (e.g., Ref.[44]) is first selected. The serpentinization front propagation velocity at temperature T and pressure P, is then estimated by scaling the reference velocity according to the relative hydrogen production rate

$$v_f(T,P) = v_{ref} \cdot \frac{R_{H2}(T,P)}{R_{H2}^{ref}(T_r,P_r)} \quad (1)$$

where v_{ref} is a reference velocity, $R_{H2}(T,P)$ is the hydrogen production rate at those conditions, $R_{H2}^{ref}(T_r,P_r)$ is the rate at reference conditions. These representative reactive blocks are then replicated throughout the modelled volume according to their temperature range, allowing locally-derived front velocities to be volumetrically integrated across the full three-dimensional reactive domain.

Estimation of H_2 solubility. Hydrogen solubility in pure water (Figs. 5e and 6e) was evaluated using the Krichevsky-Kasarnovsky (KK) relation with fugacity corrections to account for non-ideal gas behaviour. Solubility, expressed as molality m(mol kg^{-1} H_2O), is given by the equation

$$m(T,P) = m_0(T)\left(\frac{\phi(T,P)P}{P_0}\right)\exp\left[-\frac{V_\infty(P-P_0)}{RT}\right] \quad (2)$$

where T and P are temperature and pressure, P_0 =0.1 MPa is the reference pressure, R is the universal gas constant, $\phi(T,P)$ is the fugacity coefficient of H_2, and V_∞ is the partial molar volume of dissolved hydrogen at infinite dilution. A constant value V_∞ =1.7×10^{-5} m^3 mol^{-1} was adopted, consistent with experimentally-constrained values reported in Ref.[64]. Fugacity coefficients were computed using the Peng-Robinson equation of state for pure H_2, with critical properties T_c =33.19 K, P_c =1.293 MPa. Rather than prescribing an explicit temperature-dependent reference solubility, the reference molality $m_0(T)$ was inferred directly from the experimental dataset of Ref.[64]. For each isotherm, $m_0(T)$ was obtained by rearranging the KK expression for all available experimental data points (P,m), and a representative value was determined by averaging the inferred values at fixed temperature. The inferred values of $m_0(T)$

range between approximately 7×10^{-4} and 1×10^{-3} mol kg^{-1} over the investigated temperature range.

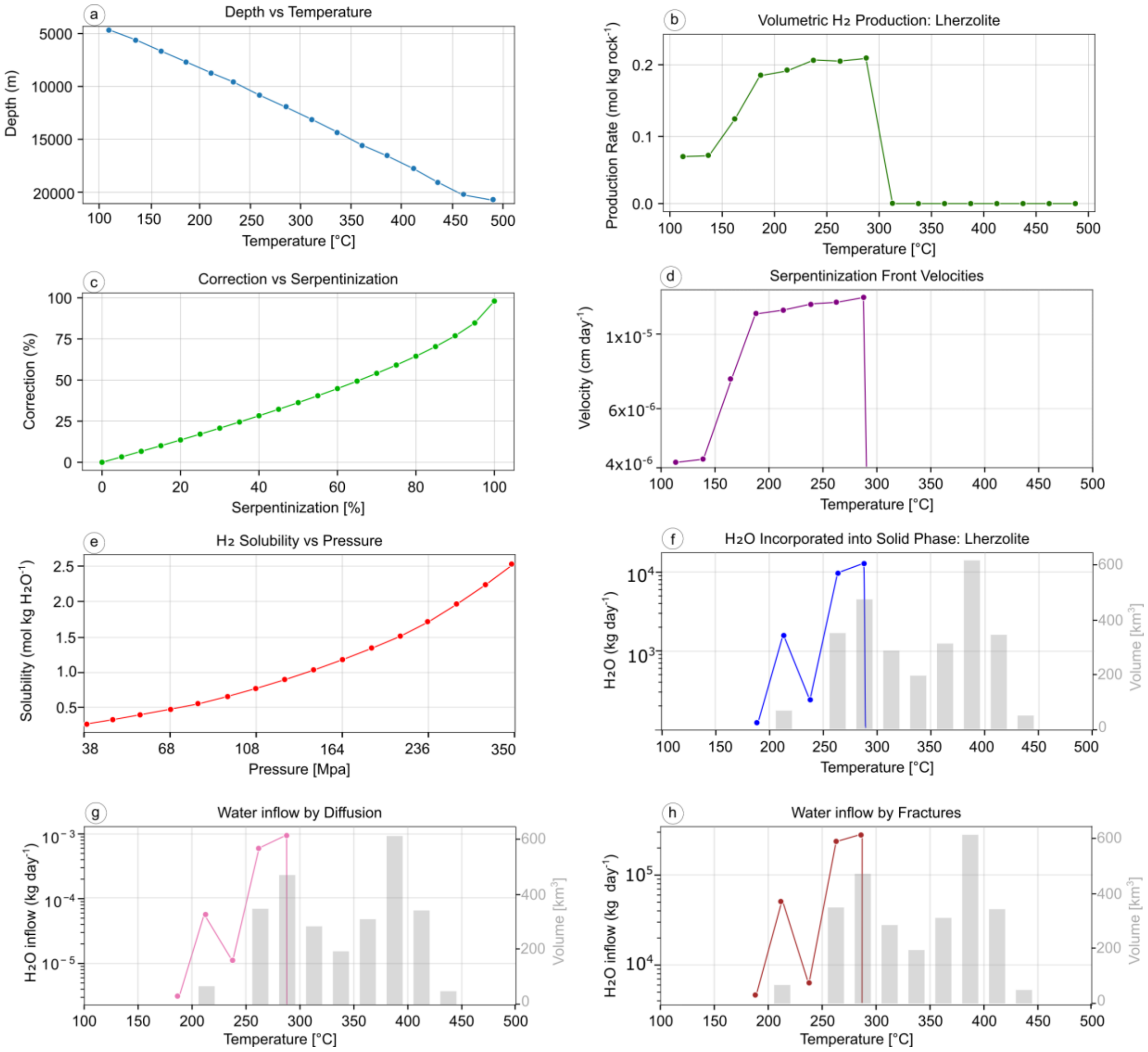

Fig. 5. Thermodynamic and kinetic parameters of serpentinization modelled for the studied pressure and temperature (P-T) conditions in the Western Pyrenees. a) Modelled temperature profile as a function of depth. b) Volumetric hydrogen production rates (mol H_2 kg rock^{-1}) estimated for each temperature interval based on thermodynamic modelling for lherzolite. c) Empirical correction factors as a function of serpentinization degree. d) Estimated serpentinization front velocities. Non-existent values reflect conditions where serpentinization is not active. e) Solubility of molecular hydrogen in water. f) Amount of water incorporated into the solid phase during serpentinization. g) Modelled water inflow rates by molecular diffusion across the serpentinized shell. h) Estimated water inflow rates across the serpentinized shell through fracture-controlled advection, based on fracture permeability models.

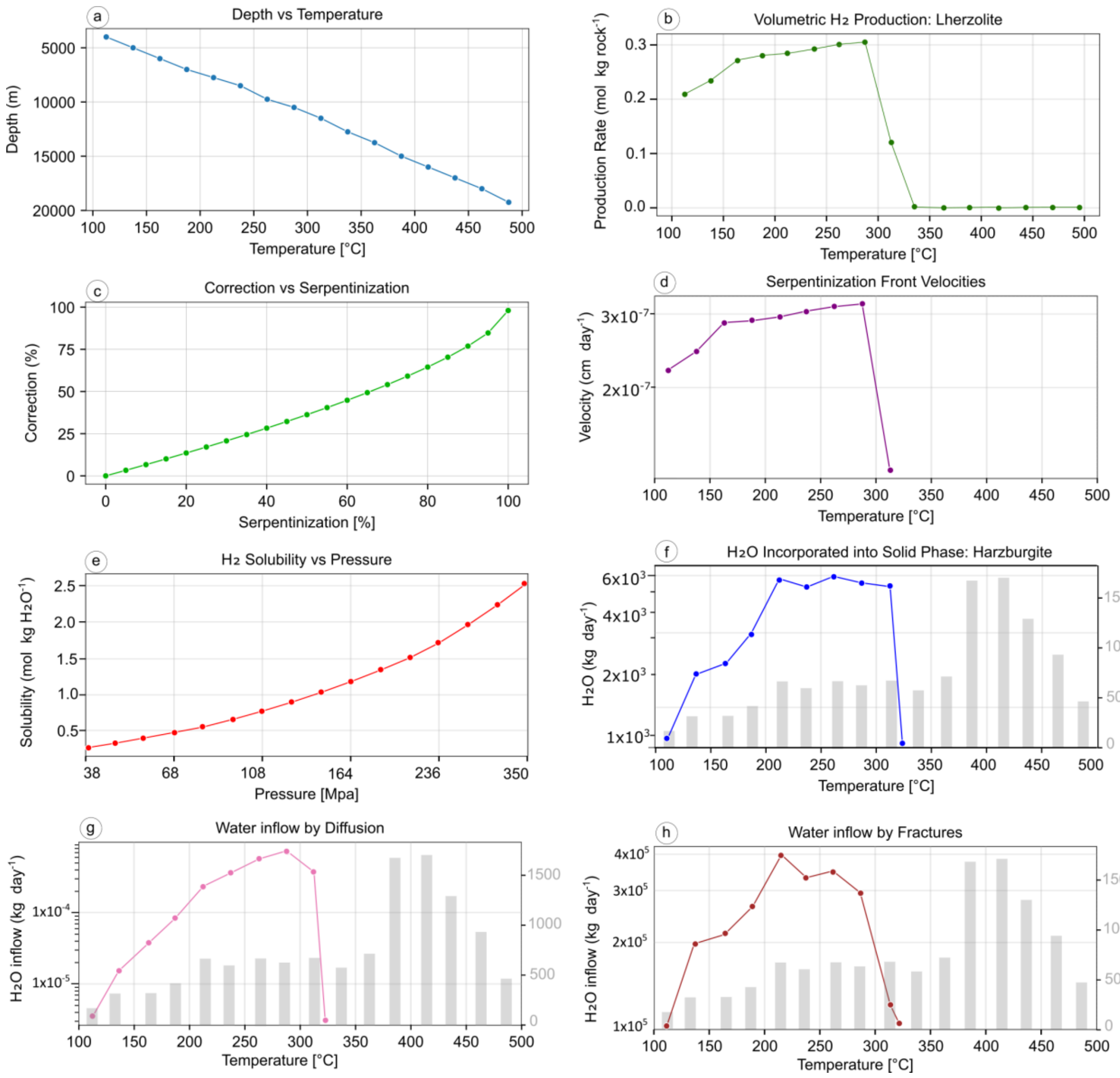

Fig. 6. Thermodynamic and kinetic parameters of serpentinization modelled for the studied pressure and temperature (P-T) conditions in Northern California. a) Modelled temperature profile as a function of depth. b) Volumetric hydrogen production rates (mol H_2 kg rock^{-1}) estimated for each temperature interval based on thermodynamic modelling for harzburgite. c) Empirical correction factors as a function of serpentinization degree. d) Estimated serpentinization front velocities. Non-existent values reflect conditions where serpentinization is not active. e) Solubility of molecular hydrogen in water. f) Amount of water incorporated into the solid phase during serpentinization. g) Modelled water inflow rates by molecular diffusion across the serpentinized shell. h) Estimated water inflow rates across the serpentinized shell through fracture-controlled advection, based on fracture permeability models.

Hydrogen production estimation. Hydrogen generation is first estimated under unconstrained conditions, where neither kinetic limitations nor hydrogen solubility and fluid saturation is taken into account (refer to Supplementary for a more detailed mathematical explanation). For each temperature and pressure condition the theoretical hydrogen generation is calculated as

$$n_{H2}^{unconstrained}(T,P) = \rho_{rock} \cdot Y_{H2}^{eq}(T,P) \cdot V_{front}(T,P) \quad (3)$$

where ρ_{rock} is the rock density and Y_{H2}^{eq} is the equilibrium H_2 yield (i.e., the maximum amount of H_2 that can be produced per unit mass of rock) derived from thermodynamic equilibrium calculations and V_{front} is the rate of advancement of the reactive volume. These values define the theoretical upper limit of the amount of hydrogen that can be produced, as determined by the intrinsic properties of the rock and the extent of serpentinization. The corresponding theoretical maximum production rate is obtained only when this equilibrium hydrogen yield is coupled to fluid flow, which controls renovation of the water and the rate at which reactive volumes are accessed.

In our models, serpentinization is associated with changes in porosity that locally enhance water inflow at the serpentinization front[65]. However, the net porosity evolution is uncertain, as fracture sealing by serpentine precipitation competes with fracture generation induced by volumetric expansion, and saturation effects progressively limit hydrogen release. A fixed daily water flow is imposed and distributed across temperature intervals in proportion to their reactive volume, ensuring preferential allocation to active zones (i.e., zones with higher fracture density and permeability). Complete mixing between incoming fluids and resident pore water is assumed, such that high inflow rates shorten fluid residence times in the pores and limit water-rock interaction. Temperature intervals that do not generate H_2 according to the thermodynamic calculations are treated as hydraulically closed and do not receive water, even if reactive rock is present. Within each interval, water is further partitioned between water that is incorporated into the rock, i.e. diffusive inflow and fracture-controlled flow (Figs. 5f-h; 6f-h). These contributions are governed by Fick's law for diffusive transport and by Darcy-type (cubic-law) flow for fracture-controlled transport. Hydrogen solubility, expressed as molality

m (mol kg^{-1} H_2O), is given by Eq. (2) and is hereafter denoted $S_{max}(T,P)$. The maximum amount of hydrogen that the available water can retain under in-situ pressure conditions is then

$$n_{H2}^{max}(\mathrm{T,P}) = S_{max}(T,P) \cdot M_{H2O}(T,P) \quad (4)$$

where $S_{max}(T,P)$ is the H_2 solubility in water and M_{H2O} includes both water filling newly formed porosity and inflow from diffusion and fractures. The model tracks the cumulative amount of hydrogen already dissolved in the water $n_{H2}^{dissolved}$. This is used to update the saturation ratio, which limits further production

$$S_{H2}^{eq}(\mathrm{T,P}) = \frac{n_{H2}^{dissolved}(\mathrm{T,P})}{n_{H2}^{max}(\mathrm{T,P})} \quad (5)$$

As saturation increases, less hydrogen can be retained in the aqueous phase. The effective hydrogen generation is thus scaled by the fraction of unsaturated capacity

$$n_{H2}^{cell}(\mathrm{T,P}) = n_{H2}^{unconstrained}(T,P) \cdot \left[1 - S_{H2}^{eq}(\mathrm{T,P})\right] \quad (6)$$

All of the above calculations are carried out for a single representative reactive block within each temperature interval (Fig. 7). The resulting hydrogen production is then extrapolated to the full reactive domain by multiplying by the number of such blocks present within the corresponding temperature range. The total hydrogen production is finally obtained by summing these block-scaled contributions over all temperature intervals and over the simulation period (typically one year).

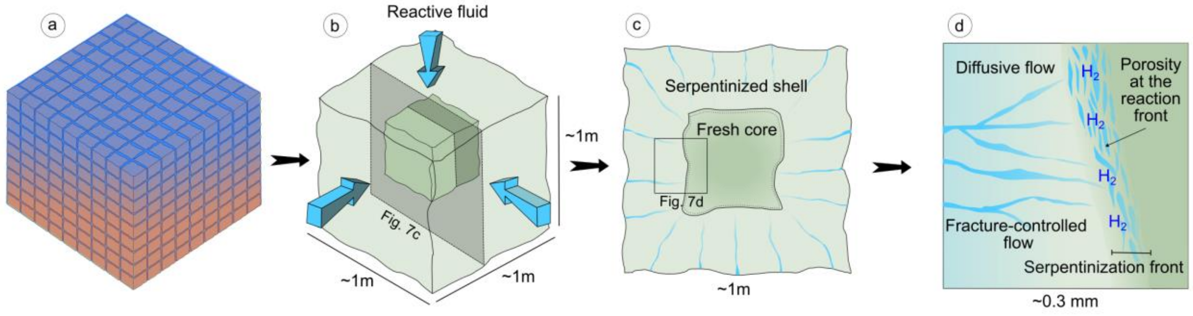

Fig. 7. Conceptual representation of the serpentinization-front architecture and associated fluid transport mechanisms. (a) The model domain is discretized into cubic elements for integration with the coupled thermodynamic and kinetic framework. (b) The reactive fluid enters each ~1 m block through external boundaries (fracture system), simulating infiltration across fracture-controlled pathways and diffusive gradients. (c) Progressive alteration results in the formation of a serpentinized shell enclosing a progressively-shrinking fresh ultramafic core. (d) At the serpentinization front, hydrogen (H_2) is generated as unaltered minerals react with the infiltrating fluid. Access of the reactive fluid to the alteration front is governed by two transport processes: (i) diffusive flow across the low-permeability serpentinized shell, parameterized as a function of temperature and pressure, and (ii) fracture-controlled flow, controlled by fracture network permeability and pressure gradients. The evolving porosity at the reaction front regulates both fluid accessibility and reaction progression.

Deep water flow capacity estimation. A Monte Carlo approach was used to test the deep-water flow potential under different fracture network configurations. Although the analysed faults have the capacity to transport the volumes of water used in our calculations, the potential source of this water has not been established. Flow is calculated as a function of permeability, fracture area, depth, pressure gradient, and fluid viscosity using Darcy's law. Fracture network geometries were generated by sampling these key parameters across physically plausible ranges. Maps of ultramafic bodies at depth were used to extract fault segments, while damage zone thickness was randomly sampled within the 1–1,000 m range to reflect the variability in fracture network widths[66]. In these zones, only a fraction of the fault length was assumed to be hydraulically active (5–30%), reflecting the strong along-strike heterogeneity of fault-zone architecture and permeability[67,68]. Fracture density was assumed to decrease with depth, consistent with increasing effective stress and reduced fracture connectivity at depth. The total fracture wall area available for flow was computed as a function of fracture count, fault length, and vertical extent. Effective permeability was sampled based on an empirical depth-dependent trend[69]. A hydraulic connection fraction of 10–25% was applied to simulate the partially connected nature of fracture networks within fault damage zones, consistent with structural models indicating heterogeneous and discontinuous fluid pathways[67,68]. This range was selected based on sensitivity testing. Pressure differentials driving flow were sampled between 60 and 140 MPa, representing lithostatic-hydrostatic contrasts at depths ranging from 1 to 12 km. Water viscosity was varied between 1×10^{-3} and 1×10^{-5} Pa·s, thus reflecting temperatures

from near-surface to ~600 °C. For each realization, permeability and all other variables were combined to estimate resulting flow rates. Success was defined as achieving or exceeding a pre-defined target flux in L day^{-1}, selected to represent a geologically plausible range. The simulations quantified success rates and identified flow rates sustainable in at least 70% of cases (Fig. 8). The geologically plausible range of deep-water fluxes considered in this study (green-shaded domains in Figs. 2 and 4) was explicitly tested using this Monte Carlo framework, and was selected to correspond to flow conditions that can be sustained in at least 70% of the realizations.

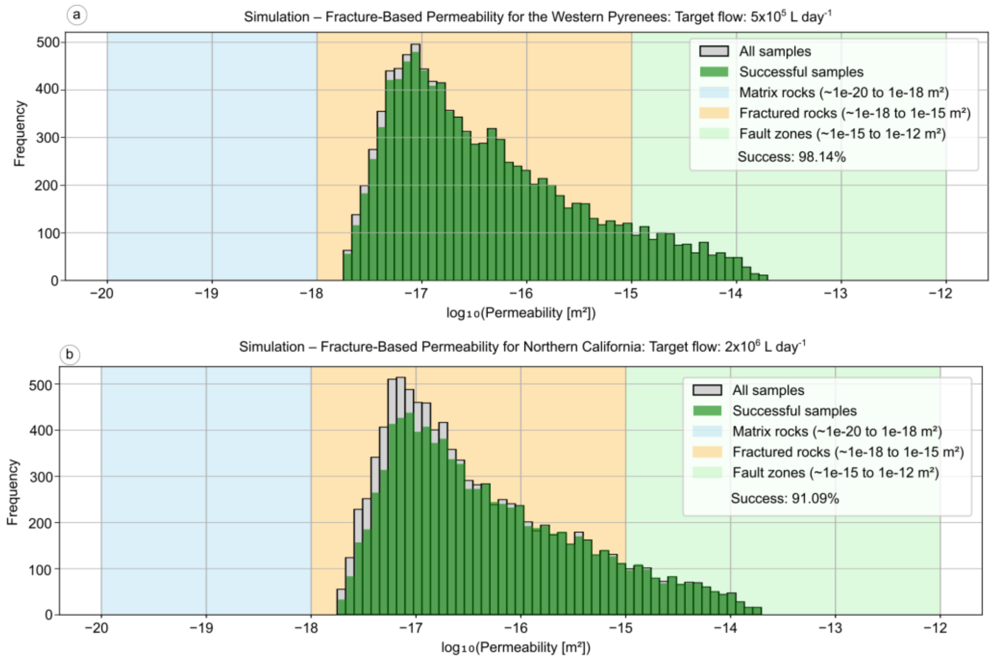

Fig. 8. Permeability distributions and hydraulic success for water delivery into serpentinizing mantle rocks. a) Western Pyrenees simulations for a target deep water flux of 5×10^5 L day^{-1}. b) Northern California simulations for a target deep water flux of 2×10^6 L day^{-1}. The histograms show the full ensemble of sampled fracture permeabilities (gray) and the subset of realizations that meet or exceed the target deep water flux (green). Shaded background regions indicate approximate permeability ranges for matrix rocks (blue: ~10^{-20}–10^{-18} m^2), fractured rocks (orange: ~10^{-18}–10^{-15} m^2), and fault zones (green: ~10^{-15}–10^{-12} m^2). Simulations consider stochastic variability in fracture network geometry, depth, fluid pressure, viscosity, and hydraulic connectivity.

Limiting-factor classification. For each temperature interval, the limiting factor of H_2 generation is diagnosed *a posteriori* from the simulated response. Intervals with zero H_2 production are classified as inactive, whereas non-zero but negligible production (>10 tonnes

year^{-1}) is treated as water-limited, reflecting insufficient fluid supply. For productive cases, dissolved H_2 accumulation relative to solubility is quantified by comparing two diagnostic timescales: the pore turnover time (τ_{turn}), defined as the time required to renew the pore-water volume, and the saturation time (τ_{sat}), defined as the characteristic time required for dissolved H_2 to accumulate to near-solubility concentrations. Hydrogen generation is classified as rate-limited when $\tau_{sat} > \tau_{turn}$ and as solubility-limited when $\tau_{sat} < \tau_{turn}$.

Uncertainty quantification and sensitivity analysis. Hydrogen production uncertainties were estimated in a Monte Carlo framework consistently for both unconstrained and constrained models. Scaling factors were applied to key parameters (such as pressure, hydrogen solubility, reaction rate, serpentinization-front velocity, reactive surface area and reactive volume) to represent model uncertainty. Each factor was sampled over a prescribed range about its reference value, representing natural variability for the physical, thermodynamic and geometric properties. The resulting ensembles were used to compute mean values and dispersion metrics (e.g., standard deviation) of total hydrogen generation under each modelling scenario. To further isolate the contribution of individual parameters to overall uncertainty, a univariate sensitivity analysis was performed. Each parameter was varied across the prescribed range while all other parameters were kept at reference values. At each sampling point, multiple Monte Carlo realizations were carried out to capture stochastic variability. A baseline simulation, with all scaling factors set to unity, was used as a reference state for normalization.

Data availability

All data supporting the findings of this study are available via Zenodo at https://doi.org/10.5281/zenodo.16847570.

Code availability

The PoNHy (Potential for Natural Hydrogen) source code used in this study is archived on Zenodo at at https://doi.org/10.5281/zenodo.16841618. The actively developed version, including the latest updates and improvements, is available on GitHub at https://github.com/RodolfoChristiansen/PoNHy.

References

1. Mayhew, L. E., Ellison, E. T., McCollom, T. M., Trainor, T. P. & Templeton, A. S. Hydrogen generation from low-temperature water–rock reactions. *Nature Geosci* **6**, 478–484 (2013).
2. McCollom, T. M. *et al.* Temperature trends for reaction rates, hydrogen generation, and partitioning of iron during experimental serpentinization of olivine. *Geochimica et Cosmochimica Acta* **181**, 175–200 (2016).
3. Deville, E. & Prinzhofer, A. The origin of N2-H2-CH4-rich natural gas seepages in ophiolitic context: A major and noble gases study of fluid seepages in New Caledonia. *Chemical Geology* **440**, 139–147 (2016).
4. Lamadrid, H. M. *et al.* Effect of water activity on rates of serpentinization of olivine. *Nat Commun* **8**, 16107 (2017).
5. Barbier, S. *et al.* A Review of H2, CH4, and Hydrocarbon Formation in Experimental Serpentinization Using Network Analysis. *Front. Earth Sci.* **8**, 209 (2020).
6. Macdonald, A. H. & Fyfe, W. S. Rate of serpentinization in seafloor environments. *Tectonophysics* **116 (1–2)**, 123–135 (1985).
7. Miller, D. J. & Christensen, N. I. *Seismic Velocities of Lower Crustal and Upper Mantle Rocks from the Slow-Spreading Mid-Atlantic Ridge, South of the Kane Transform Zone*

(MARK). Proceedings of the Ocean Drilling Program, 153 Scientific Results. vol. 153 (Ocean Drilling Program, 1997).

8. Bonnemains, D. *et al.* Magnetic signatures of serpentinization at ophiolite complexes. *Geochem Geophys Geosyst* **17**, 2969–2986 (2016).
9. Chibati, N., Géraud, Y. & Essa, K. S. Petrophysical characterization and thermal conductivity prediction of serpentinized peridotites. *Geophysical Journal International* **231**, 1786–1805 (2022).
10. Blay-Roger, R. *et al.* Natural hydrogen in the energy transition: Fundamentals, promise, and enigmas. *Renewable and Sustainable Energy Reviews* **189**, 113888 (2024).
11. Ballentine, C. J. *et al.* Natural hydrogen resource accumulation in the continental crust. *Nat Rev Earth Environ* **6**, 342–356 (2025).
12. Brunet, F. & Malvoisin, B. Can natural H2 be considered renewable? The reference case of a deep aquifer in an intracratonic sedimentary basin. *Advances in Geochemistry and Cosmochemistry* **1**, 738 (2025).
13. Truche, L. *et al.* A deep reservoir for hydrogen drives intense degassing in the Bulqizë ophiolite. *Science* **383**, 618–621 (2024).
14. Aquino, K. A. *et al.* High hydrogen outgassing from an ophiolite-hosted seep in Zambales, Philippines. *International Journal of Hydrogen Energy* **105**, 360–366 (2025).
15. Zwaan, F. *et al.* Rift-inversion orogens are potential hot spots for natural H2 generation. *Science Advances* **11(8)**, (2025).
16. Pajang, S., Mouthereau, F., Robert, A., Kumar, A. & Callot, J. A Petro-Physical Model for Serpentinized Mantle and Origin of Natural Hydrogen in the Pyrenees. *Geochem Geophys Geosyst* **26**, e2024GC011804 (2025).
17. Le Roux, V. *et al.* The Lherz spinel lherzolite: Refertilized rather than pristine mantle. *Earth and Planetary Science Letters* **259**, 599–612 (2007).

18. Tichadou, C. *et al.* Mineralogical and geochemical study of serpentinized peridotites from the North-Western Pyrenees: New insights on serpentinization along magma-poor continental passive margins. *Lithos* **406–407**, 106521 (2021).

19. Shervais, J. W. *et al.* Multi-Stage Origin of the Coast Range Ophiolite, California: Implications for the Life Cycle of Supra-Subduction Zone Ophiolites. *International Geology Review* **46**, 289–315 (2004).

20. Clerc, C., Lagabrielle, Y., Neumaier, M., Reynaud, J.-Y. & De Saint Blanquat, M. Exhumation of subcontinental mantle rocks: evidence from ultramafic-bearing clastic deposits nearby the Lherz peridotite body, French Pyrenees. *Bulletin de la Société Géologique de France* **183**, 443–459 (2012).

21. Mouthereau, F. *et al.* Placing limits to shortening evolution in the Pyrenees: Role of margin architecture and implications for the Iberia/Europe convergence: Plate convergence in the Pyrenees. *Tectonics* **33**, 2283–2314 (2014).

22. Chevrot, S. *et al.* The non-cylindrical crustal architecture of the Pyrenees. *Sci Rep* **8**, 9591 (2018).

23. Lefeuvre, N. *et al.* Native H_2 Exploration in the Western Pyrenean Foothills. *Geochem Geophys Geosyst* **22**, e2021GC009917 (2021).

24. Saspiturry, N. *et al.* Closure of a hyperextended system in an orogenic lithospheric pop-up, Western Pyrenees: The role of mantle buttressing and rift structural inheritance. *Terra Nova* **32**, 253–260 (2020).

25. Wang, Y. *et al.* The deep roots of the western Pyrenees revealed by full waveform inversion of teleseismic P waves. *Geology* **44**, 475–478 (2016).

26. Saspiturry, N., Allanic, C. & Peyrefitte, A. Serpentinization and Magmatic Distribution in a Hyperextended Rift Suture: Implication for Natural Hydrogen Exploration (Mauléon Basin, Pyrenees). *Tectonics* **43**, e2024TC008385 (2024).

27. Lehujeur, M. *et al.* Three-dimensional shear velocity structure of the Mauléon and Arzacq Basins (Western Pyrenees). *BSGF - Earth Sci. Bull.* **192**, 47 (2021).
28. Lescoutre, R. *et al.* The fingerprint of structural inheritances in the W-Pyrenees revealed by machine learning detection of seismicity. *Tectonophysics* **903**, 230685 (2025).
29. Lefeuvre, N. *et al.* Natural hydrogen migration along thrust faults in foothill basins: The North Pyrenean Frontal Thrust case study. *Applied Geochemistry* **145**, 105396 (2022).
30. Barnolas, A. *et al.* Mapa geológico de los Pirineos a escala 1: 400 000. (2008).
31. Le Maire, P. *et al.* New Magnetic compilation and interpretation of the Bay of Biscay and surrounding continental shelves. *BSGF - Earth Sci. Bull.* **192**, 58 (2021).
32. Godfrey, N. J., Beaudoin, B. C. & Klemperer, S. L. Ophiolitic basement to the Great Valley forearc basin, California, from seismic and gravity data: Implications for crustal growth at the North American continental margin. *Geological Society of America Bulletin* **109**, 1536–1562 (1997).
33. Arkula, C. *et al.* The forearc ophiolites of California formed during trench-parallel spreading: Kinematic reconstruction of the western USA Cordillera since the Jurassic. *Earth-Science Reviews* **237**, 104275 (2023).
34. Frei, L. S. & Blake, M. C. Remagnetization of the coast range ophiolite and lower part of the Great Valley Sequence in northern California and southwest Oregon. *J. Geophys. Res.* **92**, 3487–3499 (1987).
35. Smith, C. & Griggs, A. *Chromite Deposits near San Luis Obispo, San Luis Obispo County, California*. https://pubs.usgs.gov/publication/b945B (1944) doi:10.3133/b945B.
36. Constenius, K. N., Johnson, R. A., Dickinson, W. R. & Williams, T. A. Tectonic evolution of the Jurassic–Cretaceous Great Valley forearc, California: Implications for the Franciscan thrust-wedge hypothesis. *Geological Society of America Bulletin* **112**, 1703–1723 (2000).

37. McLaughlin, R. J., Moring, B. C., Hitchcock, C. S. & Valin, Z. C. *Framework Geologic Map and Structure Sections along the Bartlett Springs Fault Zone and Adjacent Area from Round Valley to Wilbur Springs, Northern Coast Ranges, California*. 60 https://doi.org/10.3133/sim3395 (2018) doi:10.3133/sim3395.

38. Langenheim, V. E., McLaughlin, R. J. & Melosh, B. L. Integrated geologic and geophysical modeling across the Bartlett Springs fault zone, northern California (USA): Implications for fault creep and regional structure. *Geosphere* **20**, 129–151 (2024).

39. Gelman, S. E., Hearon, J. S. & Ellis, G. S. Prospectivity Mapping for Geologic Hydrogen. *US Geological Survey* **1900**, (2025).

40. Morrill, P. L. *et al.* Geochemistry and geobiology of a present-day serpentinization site in California: The Cedars. *Geochimica et Cosmochimica Acta* **109**, 222–240 (2013).

41. Furlong, K. P., Villaseñor, A., Benz, H. M. & McKenzie, K. A. Formation and Evolution of the Pacific-North American (San Andreas) Plate Boundary: Constraints From the Crustal Architecture of Northern California. *Tectonics* **43**, e2023TC007963 (2024).

42. Abrajano, T. A., Pasteris, J. D. & Bacuta, G. C. Zambales ophiolite, Philippines I. Geology and petrology of the critical zone of the Acoje massif. *Tectonophysics* **168**, 65–100 (1989).

43. Farough, A., Moore, D. E., Lockner, D. A. & Lowell, R. P. Evolution of fracture permeability of ultramafic rocks undergoing serpentinization at hydrothermal conditions: An experimental study. *Geochem Geophys Geosyst* **17**, 44–55 (2016).

44. Malvoisin, B. & Brunet, F. Water diffusion-transport in a synthetic dunite: Consequences for oceanic peridotite serpentinization. *Earth and Planetary Science Letters* **403**, 263–272 (2014).

45. Everts, A. Jw., Bonnie, J. & Loosveld, R. Natural hydrogen development-potential and challenges. *International Journal of Hydrogen Energy* **142**, 26–39 (2025).

46. Astic, T., Heagy, L. J. & Oldenburg, D. W. Petrophysically and geologically guided multi-physics inversion using a dynamic Gaussian mixture model. *Geophysical Journal International* **224**, 40–68 (2020).

47. Astic, T. & Oldenburg, D. W. A framework for petrophysically and geologically guided geophysical inversion using a dynamic Gaussian mixture model prior. *Geophysical Journal International* **219**, 1989–2012 (2019).

48. Guyer, J. E., Wheeler, D. & Warren, J. A. FiPy: Partial Differential Equations with Python. *Comput. Sci. Eng.* **11**, 6–15 (2009).

49. García-Pintado, J. & Pérez-Gussinyé, M. Shallow peridotites at magma-poor rifted margins: occurrences, serpentinization, and H2 generation. Natural Hydrogen Systems: Properties, Ocurrences, Generation Mechanisms, Exploration, Storage and Transportation. *(R. Rezaee & B. Evans, Eds) De Gruyter Academic Publishing.* https://doi.org/10.1515/9783111437040-005 (2025) doi:https://doi.org/10.1515/9783111437040-005.

50. De Capitani, C. & Petrakakis, K. The computation of equilibrium assemblage diagrams with Theriak/Domino software. *American Mineralogist* **95**, 1006–1016 (2010).

51. Berman, R. G. Internally-Consistent Thermodynamic Data for Minerals in the System Na2O-K2O-CaO-MgO-FeO-Fe2O3-Al2O3-SiO2-TiO2-H2O-CO2. *Journal of Petrology* **29**, 445–522 (1988).

52. Holland, T. J. B. & Powell, R. An improved and extended internally consistent thermodynamic dataset for phases of petrological interest, involving a new equation of state for solids: THERMODYNAMIC DATASET FOR PHASES OF PETROLOGICAL INTEREST. *Journal of Metamorphic Geology* **29**, 333–383 (2011).

53. Holland, T. J. B. & Powell, R. An internally consistent thermodynamic data set for phases of petrological interest. *Journal Metamorphic Geology* **16**, 309–343 (1998).

54. Eberhard, L., Frost, D. J., McCammon, C. A., Dolejš, D. & Connolly, J. A. D. Experimental Constraints on the Ferric Fe Content and Oxygen Fugacity in Subducted Serpentinites. *Journal of Petrology* **64**, egad069 (2023).
55. Chen, H. *et al.* Induced Polarization and Magnetic Responses of Serpentinized Ultramafic Rocks From Mid-Ocean Ridges. *JGR Solid Earth* **126**, e2021JB022915 (2021).
56. Cutts, J. A. *et al.* Deducing Mineralogy of Serpentinized and Carbonated Ultramafic Rocks Using Physical Properties With Implications for Carbon Sequestration and Subduction Zone Dynamics. *Geochem Geophys Geosyst* **22**, e2021GC009989 (2021).
57. Janecky, D. R. Serpentinization Reaction Pathways: Implications for Modeling Approach. *Proceedings of the Workshop on Geochemical Modeling* 80 (1986).
58. Huang, R., Sun, W., Ding, X., Zhao, Y. & Song, M. Effect of pressure on the kinetics of peridotite serpentinization. *Phys Chem Minerals* **47**, 33 (2020).
59. Coleman, R. G. Petrologic and Geophysical Nature of Serpentinites. *Geol Soc America Bull* **82**, 897 (1971).
60. O'Hanley, D. S. Solution to the volume problem in serpentinization. *Geol* **20**, 705 (1992).
61. Plümper, O., Røyne, A., Magrasó, A. & Jamtveit, B. The interface-scale mechanism of reaction-induced fracturing during serpentinization. *Geology* **40**, 1103–1106 (2012).
62. Melosh, B. L. Fault Initiation in Serpentinite. *Geochem Geophys Geosyst* **20**, 2626–2646 (2019).
63. Uno, M. & Kirby, S. Evidence for multiple stages of serpentinization from the mantle through the crust in the Redwood City Serpentinite mélange along the San Andreas Fault in California. *Lithos* **336–337**, 276–292 (2019).
64. Longe, P. O., Davoodi, S., Mehrad, M. & Wood, D. A. Combined Deep Learning and Optimization for Hydrogen-Solubility Prediction in Aqueous Systems Appropriate for Underground Hydrogen Storage Reservoirs. *Energy Fuels* **38**, 22031–22049 (2024).

65. Chogani, A. & Plümper, O. Decoding the nanoscale porosity in serpentinites from multidimensional electron microscopy and discrete element modelling. *Contrib Mineral Petrol* **178**, 78 (2023).
66. Choi, J.-H., Edwards, P., Ko, K. & Kim, Y.-S. Definition and classification of fault damage zones: A review and a new methodological approach. *Earth-Science Reviews* **152**, 70–87 (2016).
67. Caine, J. S., Evans, J. P. & Forster, C. B. Fault zone architecture and permeability structure. *Geol* **24**, 1025 (1996).
68. Faulkner, D. R. *et al.* A review of recent developments concerning the structure, mechanics and fluid flow properties of fault zones. *Journal of Structural Geology* **32**, 1557–1575 (2010).
69. Manning, C. E. & Ingebritsen, S. E. Permeability of the continental crust: Implications of geothermal data and metamorphic systems. *Reviews of Geophysics* **37**, 127–150 (1999).

Acknowledgements

This work was developed within the framework of the HyAfrica project, part of the Long-term Europe-Africa Partnership on Renewable Energy (LEAP-RE) programme. LEAP-RE has received funding from the European Union's Horizon 2020 Research and Innovation Programme under Grant Agreement No. 963530. German partners of HyAfrica received funding from the German Ministry for Education and Research (BMBF), LIAG under grant 03SF0680B. Funding to J.G.-P. was provided by the German Research Foundation (DFG) under Germany's Excellence Strategy "The Ocean Floor - Earth's Uncharted Interface" EXC-2077, Project 390741603. We would like to thank Tom McCollom for his generous discussions regarding hydrogen production rates in open systems. We are grateful to David C. Tanner for his valuable support with the editing and improvement of the manuscript. We acknowledge the

SimPEG project for providing open-source geophysical inversion libraries, which greatly facilitated the development of our modelling framework. Any use of trade, firm, or product names is for descriptive purposes only and does not imply endorsement by the U.S. Government.

Author contributions

R.C. designed the methodology, developed and implemented the full modelling framework, wrote the source code, and led the writing of the manuscript. M.S. contributed to the development of the code, assisted with the geophysical inversion and writing of the manuscript. C.O.-H. provided key input on the methodology and serpentinization processes, contributed to the conceptual framework and writing of the manuscript. G.G. contributed to the tectonic interpretation and writing of the manuscript. N.S. and S.C. provided geological and geophysical data for the Western Pyrenees and contributed to the regional interpretation and scientific discussions. V.L. contributed geological data and regional interpretation for the Northern California case study, as well as writing of the manuscript and scientific discussions. J.G.-P. provided guidance on the thermodynamic modelling, contributed to the conceptual development of the study and scientific discussions. G. Gabriel supervised the project, contributed to discussions throughout and writing of the manuscript. All authors reviewed the manuscript and contributed to the interpretation of the results.

Competing interests

The authors declare no competing interests.

Corresponding author

Correspondence to Rodolfo Christiansen: rodo_christiansen@hotmail.com or Rodolfo.Christiansen@liag-institut.de

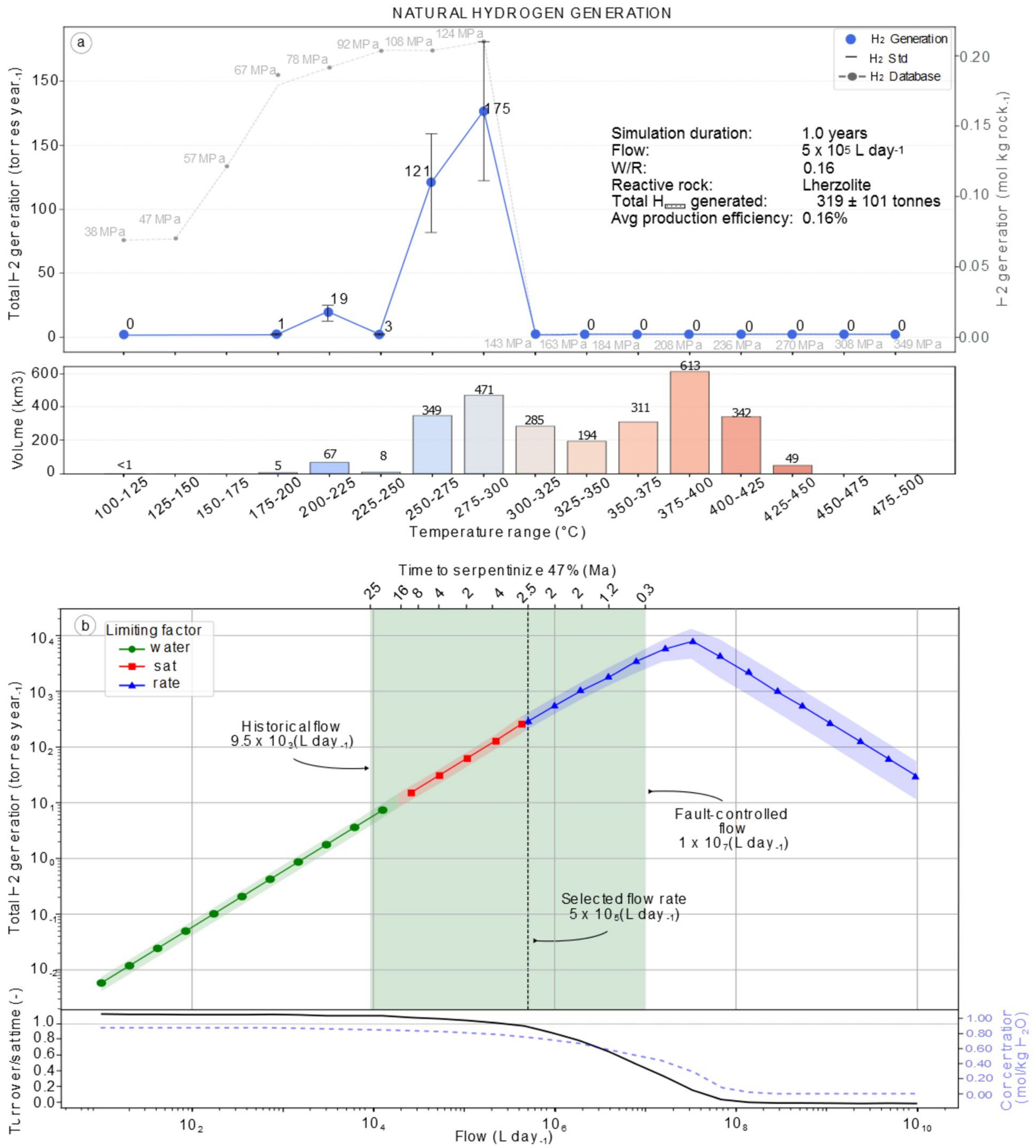

NATURAL HYDROGEN GENERATION
a
H2 Generation
H2 Std
H2 Database
Simulation duration: 1.0 years
Flow: 5 x 10^5 L day-1
W/R: 0.16
Reactive rock: Lherzolite
Total H2 generated: 319 ± 101 tonnes
Avg production efficiency: 0.16%
Total H2 generation (tonnes year-1)
H2 generation (mol kg rock-1)
Volume (km3)
Temperature range (°C)
100-125
125-150
150-175
175-200
200-225
225-250
250-275
275-300
300-325
325-350
350-375
375-400
400-425
425-450
450-475
475-500
b
Time to serpentinize 47% (Ma)
Limiting factor
water
sat
rate
Historical flow
9.5 x 10^3 (L day-1)
Fault-controlled flow
1 x 10^7 (L day-1)
Selected flow rate
5 x 10^5 (L day-1)
Total H2 generation (tonnes year-1)
Turnover/sat time (-)
Concentration (mol/kg H2O)
Flow (L day-1)

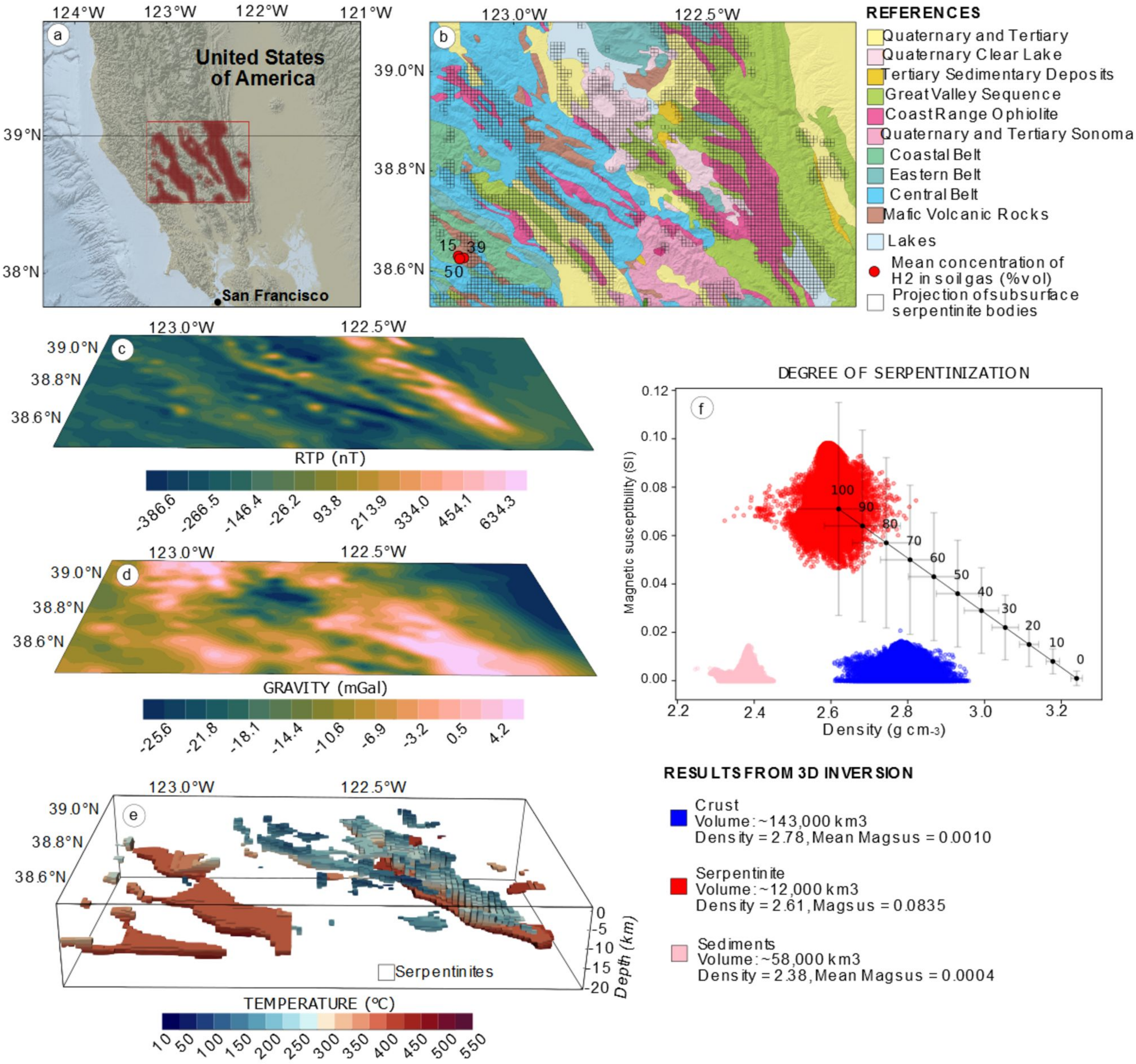

124°W 123°W 122°W 121°W
United States of America
39°N
38°N
San Francisco
123.0°W 122.5°W
39.0°N
38.8°N
38.6°N
15 39 50
REFERENCES
Quaternary and Tertiary
Quaternary Clear Lake
Tertiary Sedimentary Deposits
Great Valley Sequence
Coast Range Ophiolite
Quaternary and Tertiary Sonoma
Coastal Belt
Eastern Belt
Central Belt
Mafic Volcanic Rocks
Lakes
Mean concentration of H2 in soil gas (%vol)
Projection of subsurface serpentinite bodies
RTP (nT)
-386.6 -266.5 -146.4 -26.2 93.8 213.9 334.0 454.1 634.3
GRAVITY (mGal)
-25.6 -21.8 -18.1 -14.4 -10.6 -6.9 -3.2 0.5 4.2
Serpentinites
Depth (km)
0 -5 -10 -15 -20
TEMPERATURE (°C)
10 50 100 150 200 250 300 350 400 450 500 550
DEGREE OF SERPENTINIZATION
Magnetic susceptibility (SI)
Density (g cm-3)
100 90 80 70 60 50 40 30 20 10 0
RESULTS FROM 3D INVERSION
Crust
Volume: ~143,000 km3
Density = 2.78, Mean Magsus = 0.0010
Serpentinite
Volume: ~12,000 km3
Density = 2.61, Magsus = 0.0835
Sediments
Volume: ~58,000 km3
Density = 2.38, Mean Magsus = 0.0004

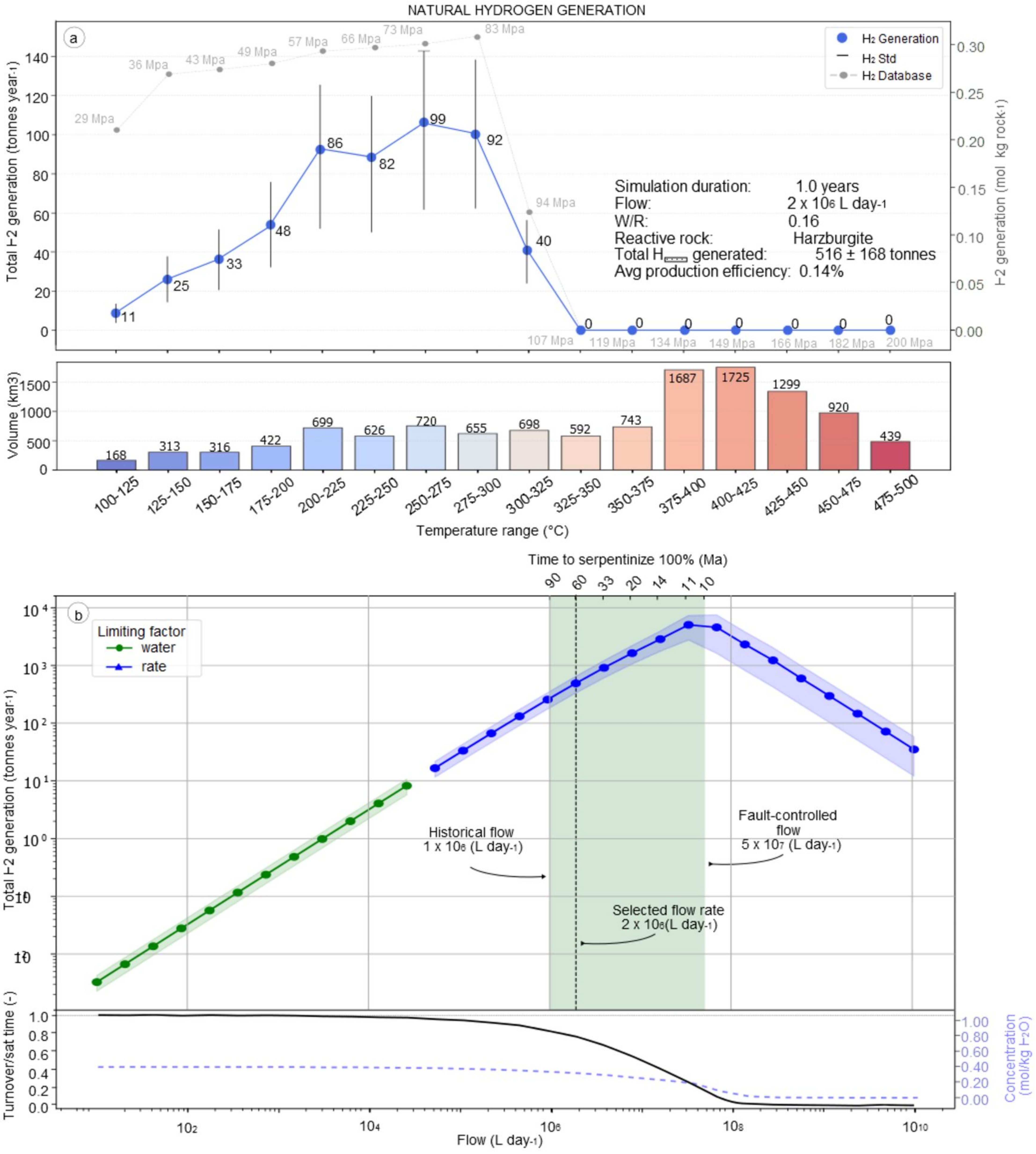

NATURAL HYDROGEN GENERATION
a
Total H2 generation (tonnes year-1)
H2 generation (mol kg rock-1)
H2 Generation
H2 Std
H2 Database
Simulation duration: 1.0 years
Flow: 2 x 10^6 L day-1
W/R: 0.16
Reactive rock: Harzburgite
Total H2 generated: 516 ± 168 tonnes
Avg production efficiency: 0.14%
Volume (km3)
Temperature range (°C)
b
Time to serpentinize 100% (Ma)
Limiting factor
water
rate
Historical flow
1 x 10^6 (L day-1)
Fault-controlled flow
5 x 10^7 (L day-1)
Selected flow rate
2 x 10^6 (L day-1)
Turnover/sat time (-)
Concentration (mol/kg H2O)
Flow (L day-1)

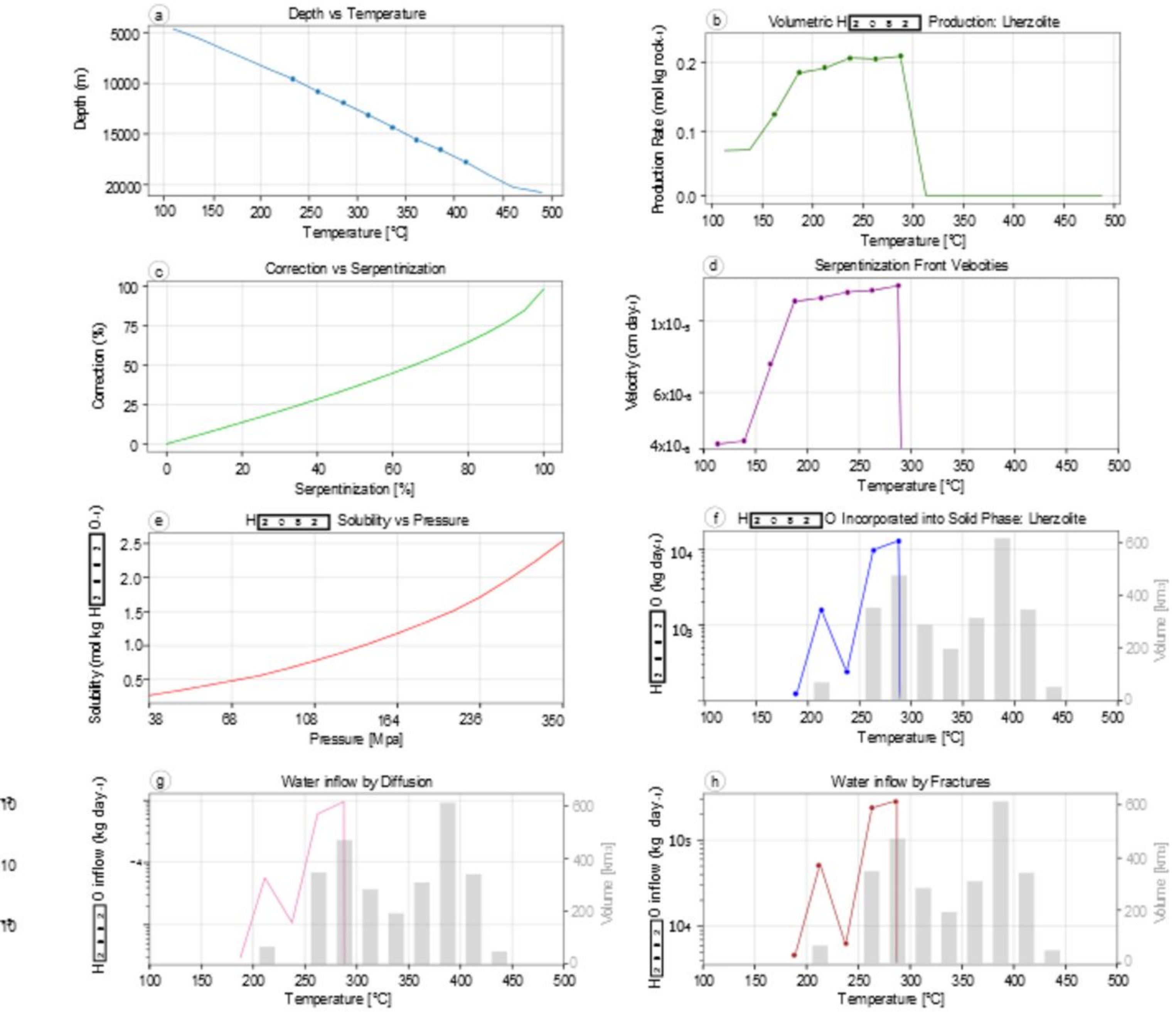

a
Depth vs Temperature
Depth (m)
Temperature [°C]
b
Volumetric H Production: Lherzolite
Production Rate (mol kg rock-1)
Temperature [°C]
c
Correction vs Serpentinization
Correction (%)
Serpentinization [%]
d
Serpentinization Front Velocities
Velocity (cm day-1)
Temperature [°C]
e
H Solubility vs Pressure
Solubility (mol kg H O-1)
Pressure [Mpa]
f
H O Incorporated into Solid Phase: Lherzolite
H O (kg day-1)
Volume [km3]
Temperature [°C]
g
Water inflow by Diffusion
H O inflow (kg day-1)
Volume [km3]
Temperature [°C]
h
Water inflow by Fractures
H O inflow (kg day-1)
Volume [km3]
Temperature [°C]

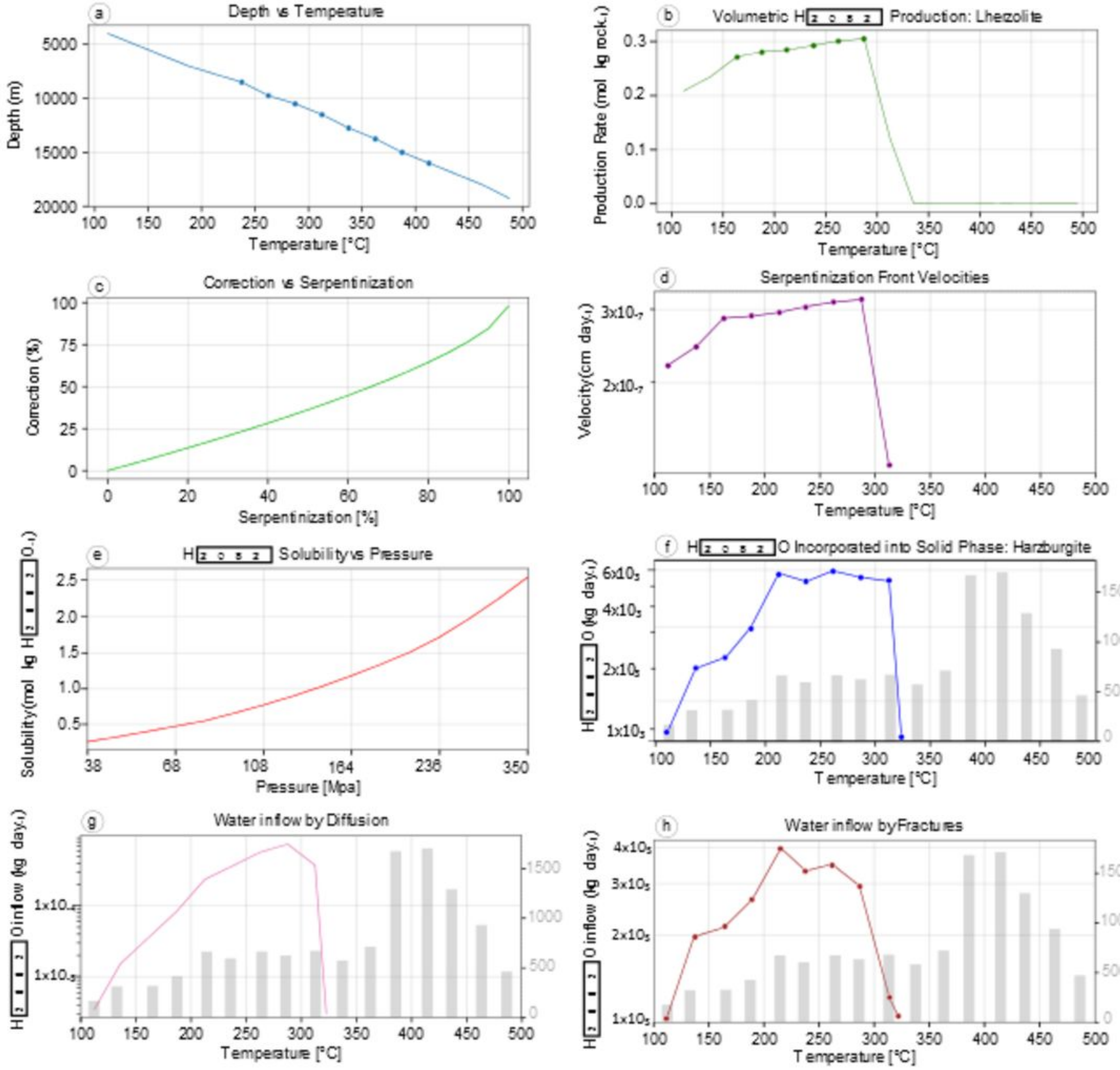

a
Depth vs Temperature
Depth (m)
Temperature [°C]
b
Volumetric H Production: Lherzolite
Production Rate (mol kg rock)
Temperature [°C]
c
Correction vs Serpentinization
Correction (%)
Serpentinization [%]
d
Serpentinization Front Velocities
Velocity(cm day)
Temperature [°C]
e
H Solubility vs Pressure
Solubility(mol kg H O)
Pressure [Mpa]
f
H O Incorporated into Solid Phase: Harzburgite
H O (kg day)
Temperature [°C]
g
Water inflow by Diffusion
H O inflow (kg day)
Temperature [°C]
h
Water inflow by Fractures
H O inflow (kg day)
Temperature [°C]

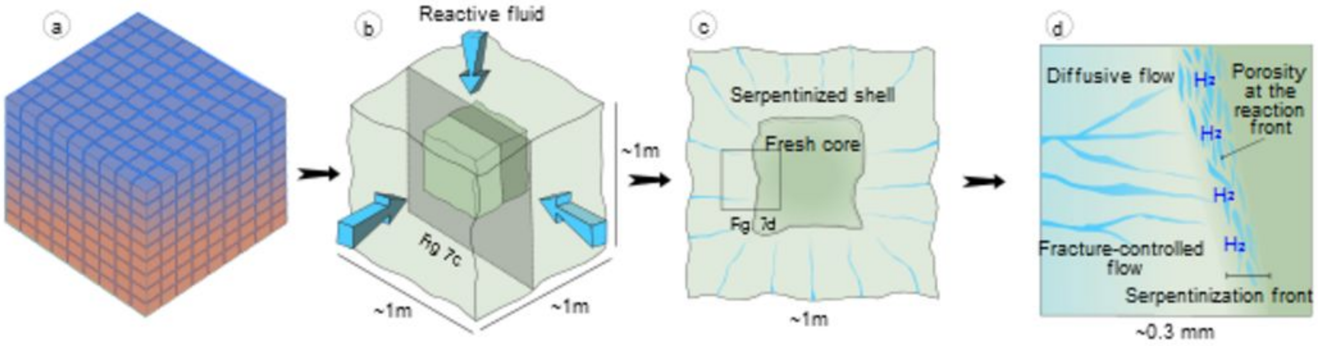
a
b
Reactive fluid
~1m
Fig 7c
~1m
~1m
c
Serpentinized shell
Fresh core
Fig 7d
~1m
d
Diffusive flow
Porosity at the reaction front
H2
H2
H2
H2
Fracture-controlled flow
Serpentinization front
~0.3 mm

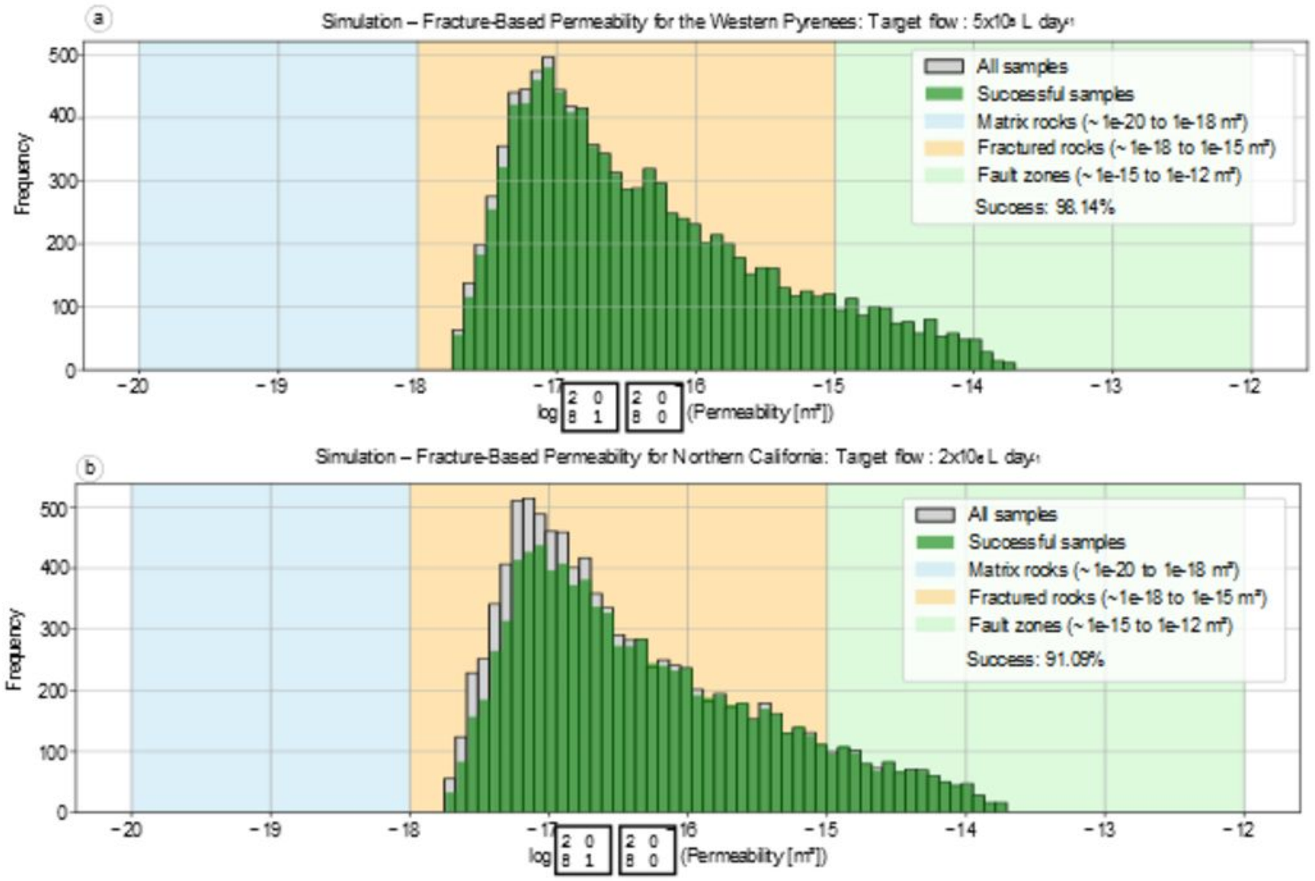
a
Simulation – Fracture-Based Permeability for the Western Pyrenees: Target flow : 5x10 L day
All samples
Successful samples
Matrix rocks (~ 1e-20 to 1e-18 m²)
Fractured rocks (~ 1e-18 to 1e-15 m²)
Fault zones (~ 1e-15 to 1e-12 m²)
Success: 98.14%
Frequency
log (Permeability [m²])
b
Simulation – Fracture-Based Permeability for Northern California: Target flow : 2x10 L day
All samples
Successful samples
Matrix rocks (~ 1e-20 to 1e-18 m²)
Fractured rocks (~1e-18 to 1e-15 m²)
Fault zones (~ 1e-15 to 1e-12 m²)
Success: 91.09%
Frequency
log (Permeability [m²])
